# Do beta-adrenergic blocking agents increase asthma exacerbation? A network meta-analysis of randomized controlled trials

**DOI:** 10.1038/s41598-020-79837-3

**Published:** 2021-01-11

**Authors:** Kuo-Yang Huang, Ping-Tao Tseng, Yi-Cheng Wu, Yu-Kang Tu, Brendon Stubbs, Kuan-Pin Su, Yutaka J. Matsuoka, Chih-Wei Hsu, Ching-Hsiung Lin, Yen-Wen Chen, Pao-Yen Lin

**Affiliations:** 1 Division of Chest Medicine, Department of Internal Medicine, Changhua Christian Hospital, Changhua, Taiwan; 2 Prospect Clinic for Otorhinolaryngology and Neurology, Kaohsiung, Taiwan; 3 WinShine Clinic in Specialty of Psychiatry, Kaohsiung, Taiwan; 4 Institute of Biomedical Sciences, National Sun Yat-Sen University, Kaohsiung, Taiwan; 5 Department of Psychology, College of Medical and Health Science, Asia University, Taichung, Taiwan; 6 Department of Sports Medicine, Landseed International Hospital, Taoyuan, Taiwan; 7 Institute of Epidemiology and Preventive Medicine, College of Public Health, National Taiwan University, Taipei, Taiwan; 8 Department of Dentistry, National Taiwan University Hospital, Taipei, Taiwan; 9 Department of Psychological Medicine, Institute of Psychiatry, Psychology and Neuroscience, King’s College London, London, UK; 10 Physiotherapy Department, South London and Maudsley NHS Foundation Trust, London, UK; 11 Positive Ageing Research Institute (PARI), Faculty of Health, Social Care, Medicine and Education, Anglia Ruskin University, Chelmsford, UK; 12 Department of Psychiatry and Mind–Body Interface Laboratory (MBI-Lab), China Medical University Hospital, Taichung, Taiwan; 13 College of Medicine, China Medical University, Taichung, Taiwan; 14 An-Nan Hospital, China Medical University, Tainan, Taiwan; 15 Division of Health Care Research, Center for Public Health Sciences, National Cancer Center Japan, Tokyo, Japan; 16 Department of Psychiatry, Kaohsiung Chang Gung Memorial Hospital, Chang Gung University College of Medicine, 123 Dapi Rd., Niaosong Dist., Kaohsiung, 833 Taiwan; 17 Institute for Translational Research in Biomedical Sciences, Kaohsiung Chang Gung Memorial Hospital, Kaohsiung, Taiwan

**Keywords:** Respiratory tract diseases, Disease prevention

## Abstract

Beta-adrenergic blocking agents (abbreviated as beta-blockers) have been used for treating various cardiovascular diseases. However, the potential for asthma exacerbation is one of the major adverse effects of beta-blockers. This study aimed to compare the level of risk for an asthma attack in patients receiving various beta-blockers. We searched for randomized controlled trials (RCTs) of either placebo-controlled or active-controlled design. The current network meta-analysis (NMA) was conducted under a frequentist model. The primary outcome was the incidence of asthmatic attack. A total of 24 RCTs were included. Overall NMA revealed that only oral timolol [risk ratio (RR) = 3.35 (95% confidence interval (CI) 1.04–10.85)] and infusion of propranolol [RR = 10.19 (95% CI 1.29–80.41)] were associated with significantly higher incidences of asthma attack than the placebo, whereas oral celiprolol [RR = 0.39 (95% CI 0.04–4.11)], oral celiprolol and propranolol [RR = 0.46 (95% CI 0.02–11.65)], oral bisoprolol [RR = 0.46 (95% CI 0.02–11.65)], oral atenolol [RR = 0.51 (95% CI 0.20–1.28)], infusion of practolol [RR = 0.80 (95% CI 0.03–25.14)], and infusion of sotalol [RR = 0.91 (95% CI 0.08–10.65)] were associated with relatively lower incidences of asthma attack than the placebo. In participants with a baseline asthma history, in addition to oral timolol and infusion of propranolol, oral labetalol, oxprenolol, propranolol, and metoprolol exhibited significantly higher incidences of asthma attack than did the placebo. In conclusion, oral timolol and infusion of propranolol were associated with a significantly higher risk of developing an asthma attack in patients, especially in those with a baseline asthma history, and should be avoided in patients who present a risk of asthma.

**Trial registration:** PROSPERO CRD42020190540.

## Introduction

Beta-adrenergic blocking agents (or beta-blockers) have been frequently used to treat various cardiovascular disorders such as hypertension, ischemic heart disease, cardiac arrhythmias, and congestive heart failure^1–4^. Clinicians often refrain from prescribing them for patients with an underlying disease of concern to adverse events, such as asthma, diabetes mellitus, and peripheral artery disease^5^. In fact, acute bronchoconstriction with leading asthma exacerbation is the most crucial side effect of beta-blockers, for which several review articles and practice guidelines have advised avoiding the use of beta-blockers in patients with asthma^6–9^. Furthermore, beta-blockers are one of the first-line treatment agents for thyrotoxicosis^10^ and essential tremor^11^, as well as for preventing variceal bleeding in patients with portal hypertension^12^ and aortic aneurysm in Marfan syndrome^13^. Although there is considerable evidence for the effectiveness and benefits of beta-blockers in treating these diseases, the associated adverse events such as an asthma attack create a dilemma for physicians considering treatment with beta-blockers for patients with asthma.

A large cohort study has demonstrated that the benefits outweigh the risks of cardioselective beta-blocker therapy in patients with asthma for long-term management of heart failure or decreased 1-year mortality rate after myocardial infarction^14^. Several randomized controlled trials (RCTs) or pairwise meta-analyses have reported the various influences of beta-blockers in pulmonary function, symptom changes, or asthma attack separately^6,7,15,16^. Salpeter et al. reported that patients with reactive airway disease who received a single dose of cardioselective beta-blockers presented a 7.46% decrease in forced expiratory volume in one second (FEV_1_)^7^. Another population-based nested case–control study demonstrated that nonselective beta-blockers were associated with a significantly increased risk of asthma exacerbation^6^. Moreover, the risk for asthma and adverse effects on pulmonary function with the use of non-cardioselective beta-blockers was found to be more prominent than that with the use of cardioselective beta-blockers^17^. Therefore, it is an important consideration in clinical practice to assess which beta-blockers have been shown to significantly increase the risk for serious asthma exacerbation and which have not. Nevertheless, the results could not be obtained by means of the traditional RCTs or meta-analysis studies in the past.

Network meta-analysis (NMA) of existing RCTs enables the estimation of the comparative efficacy or risk and the understanding of the relative merits of multiple interventions, which cannot be achieved in traditional pairwise meta-analysis^18^. Therefore, we conducted a comprehensive NMA to compare the risk of developing an adverse asthma attack in patients receiving treatment with various beta-blockers.

## Methods

The detailed description of method is listed in eTable 1. In brief, it follows the preferred reporting items for systematic reviews and meta-analyses extension guideline (eTable 2)^19^ and follows the previous NMAs^20–23^. The current frequentist model-based NMA, which included only RCTs, was conducted to investigate the incidence of asthma attack after beta-blocking agent treatment in patients with and without a baseline asthma history. To examine the risk of asthma attack after treatment with various beta-blocking agents, we searched for RCTs specifically designed to assess the risk among asthmatics by using keywords of “asthma”, “dyspnea”, “bronchoconstriction, “bronchial constriction”, “bronchial hyperreactivity”, “respiratory sound”, “wheeze”, or “wheezing” (eTable 3). Because decreased pulmonary function associated with beta-blocking agents would not always result in clinical symptoms, we did not select for changes in pulmonary function as our primary outcome^24^. The primary outcome was the incidence of asthma attacks after treatment with beta-blocking agents compared with control conditions in patients with or without baseline asthma history. The definition of an asthma attack could be deterioration in symptoms, increased use of rescue bronchodilators, emergency room visits for asthma, and requiring systemic corticosteroids^25^. We estimated the summary risk ratios (RRs) with 95% confidence intervals (CIs) for categorical variables and further applied a 0.5 zero-cell correction during the procedure of the meta-analysis. To minimize the potential bias caused by imputing 0.5 to zero-cells in the data, we conducted a sensitivity test by removing trials with zero or 100% events in their treatment arms. Heterogeneity among the included studies was evaluated using the tau value, which is the estimated standard deviation of the effect across the included studies. To provide additional clinical application, we calculated the surface under the cumulative ranking curve (SUCRA) among the preventive effects of all treatments for the target outcomes to rank the potential superiority among the investigated treatments. We also performed a subgroup analysis focusing on patients with a definite baseline asthma history. Finally, we evaluated the potential inconsistency using the loop-specific approach, the node-splitting method, and the design-by-treatment model.

## Results

**Figure 1:**
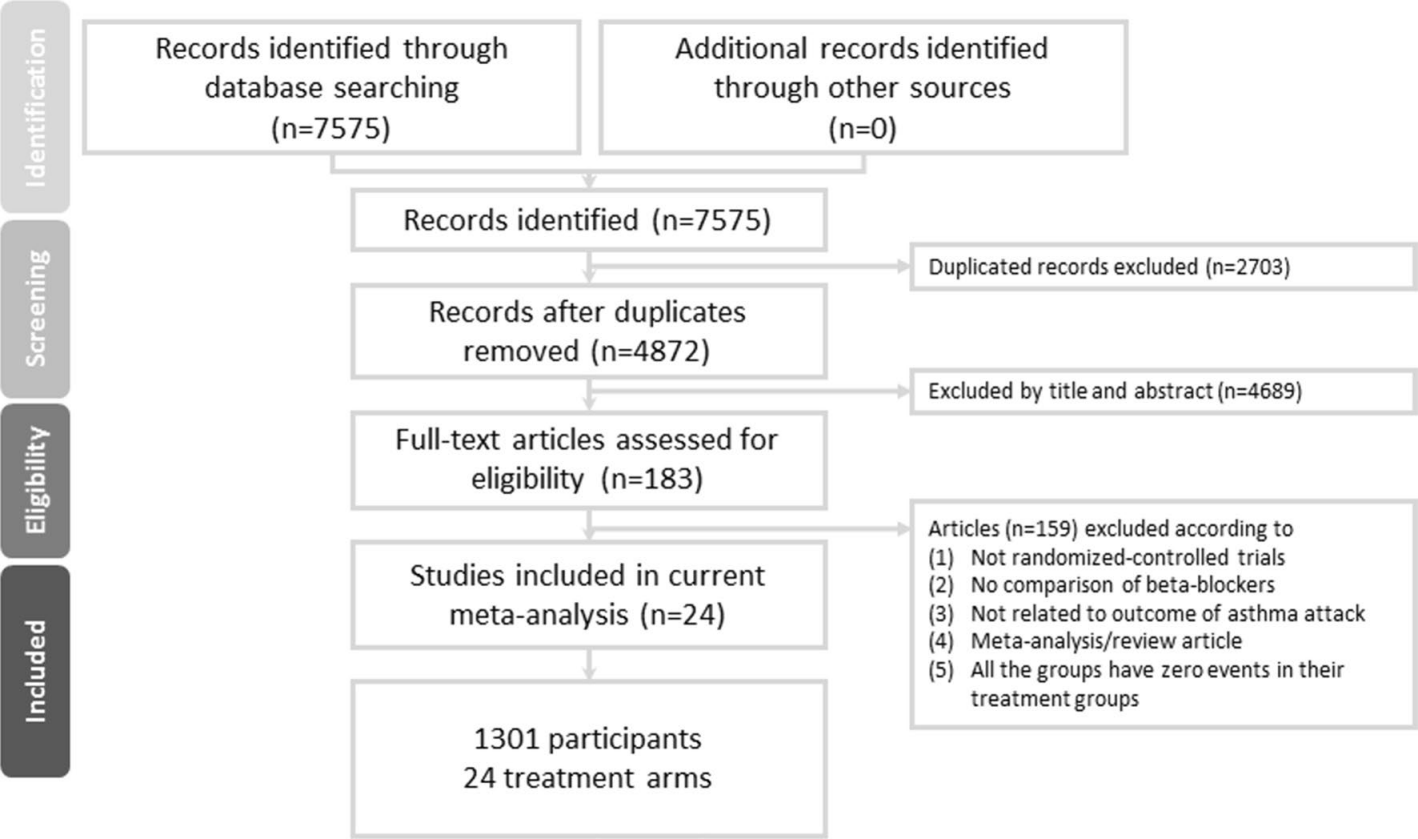
Flowchart of the current network meta-analysis.

**Figure 2:**
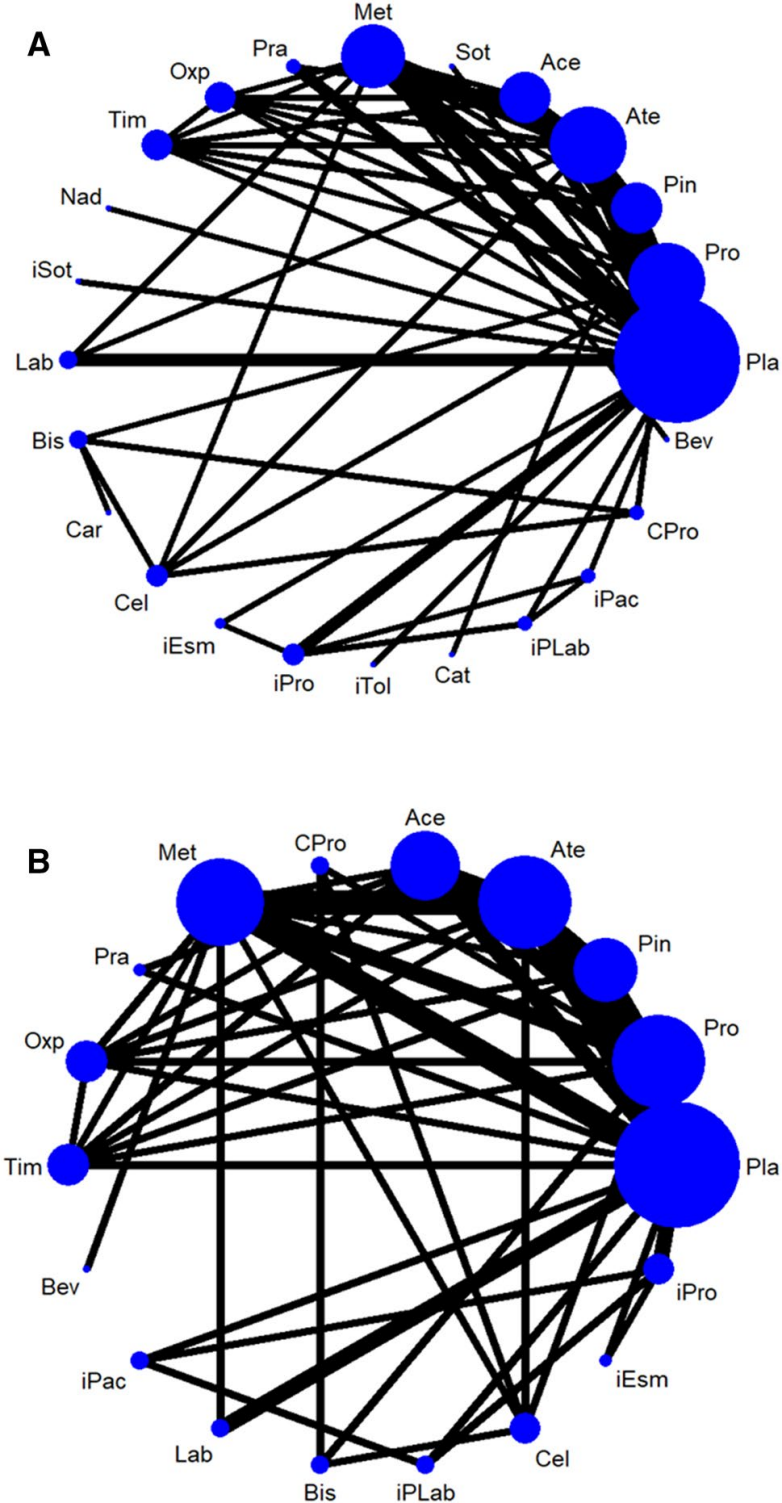
The network structure of (**A**) individual beta-blocking agents among the overall participants and (**B**) individual beta-blocking agents among participants with a baseline history of asthma. The lines between nodes represent direct comparisons in various trials, and the size of each circle is proportional to the size of the population involved in each specific treatment. The thickness of the lines is proportional to the number of trials connected to the network. *Ace* oral acebutolol, *Ate* oral atenolol, *Bev* oral bevantolol, *Bis* oral bisoprolol, *Car* oral carvedilol, *Cat* oral carteolol, *Cel* oral celiprolol, *CI* confidence interval, *CPro* oral celiprolol and propranolol, *iEsm* infusion of esmolol, *iPac* infusion of practolol, *iPLab* infusion of propranolol and labetalol, *iPro* infusion of propranolol, *iSot* infusion of sotalol, *iTol* infusion of tolamolol, *Lab* oral dilevalol or oral labetalol, *Met* oral metoprolol, *Nad* oral nadolol, *Oxp* oral oxprenolol, *Pin* oral pindolol, *Pla* Placebo/control, *Pra* oral practolol, *Pro* oral propranolol, *Sot* oral sotalol, *Tim* oral timolol.

After the initial screening procedure, a total of 183 articles were considered for full-text review (Fig. 1). However, 159 were excluded for various reasons (see Fig. 1 and eTable 4). Finally, 24 articles were included in the current study (eTable 5), among which 13 provided evidence related to patients with a definite baseline asthma history. Figure 2A depicts the entire geometric distribution of the treatment arms.

### Characteristics of the included studies

A total of 1301 participants were included. The mean age of the participants was 54.5 years (range 22.0–77.3 years, 25–75% interquartile = 39.6 and 61.0 years), and the mean female proportion was 22.6% (range 0.0–60.0%, 25–75% interquartile = 10.3% and 35.1%). The baseline characteristics of the included participants are listed in eTable 5.

The duration of beta-blocking agent prescription ranged from only once before evaluation through 14 weeks.

### Overall incidence of asthma attack after receiving beta-blocking agents

A total of 24 articles with 24 treatment arms were investigated in the current NMA, including placebo or control, oral propranolol, oral pindolol, oral atenolol, oral acebutolol, oral sotalol, oral metoprolol, oral practolol, oral oxprenolol, oral timolol, oral nadolol, infusion of sotalol, oral labetalol, oral bisoprolol, oral carvedilol, oral celiprolol, infusion of esmolol, infusion of propranolol, infusion of tolamolol, oral carteolol, infusion of propranolol and labetalol, infusion of practolol, oral celiprolol and propranolol, and oral bevantolol.

**Figure 3:**
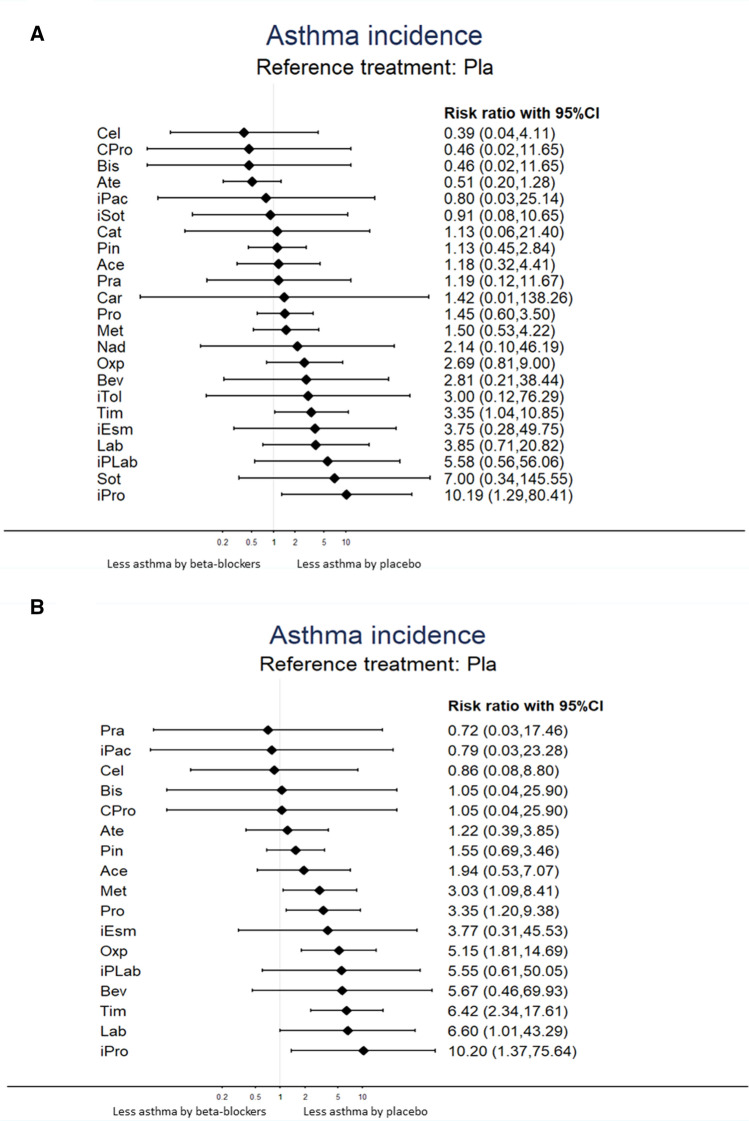
Forest plot of the incidence of asthma attack by (**A**) individual beta-blocking agents among the overall participants and (**B**) individual beta-blocking agents among participants with a baseline history of asthma. An effect size (presented as risk ratio) of < 1 corresponds to a lower incidence of asthma attack by specified beta-blocking agent compared with that by the placebo or control group. *Ace* oral acebutolol, *Ate* oral atenolol, *Bev* oral bevantolol, *Bis* oral bisoprolol, *Car* oral carvedilol, *Cat* oral carteolol, *Cel* oral celiprolol, *CI* confidence interval, *CPro* oral celiprolol and propranolol, *iEsm* infusion of esmolol, *iPac* infusion of practolol, *iPLab* infusion of propranolol and labetalol, *iPro* infusion of propranolol, *iSot* infusion of sotalol, *iTol* infusion of tolamolol, *Lab* oral dilevalol or oral labetalol, *Met* oral metoprolol, *Nad* oral nadolol, *Oxp* oral oxprenolol, *Pin* oral pindolol, *Pla* Placebo/control, *Pra* oral practolol, *Pro* oral propranolol, *Sot* oral sotalol, *Tim* oral timolol.

The NMA revealed that only oral timolol [RR = 3.35 (95% CI 1.04–10.85)] and infusion of propranolol [RR = 10.19 (95% CI 1.29–80.41)] were associated with a significantly higher incidence of asthma attack than the placebo or control groups. In contrast, oral celiprolol [RR = 0.39 (95% CI 0.04–4.11)], oral celiprolol and propranolol [RR = 0.46 (95% CI 0.02–11.65)], oral bisoprolol [RR = 0.46 (95% CI 0.02–11.65)], oral atenolol [RR = 0.51 (95% CI 0.20–1.28)], infusion of practolol [RR = 0.80 (95% CI 0.03–25.14)], and infusion of sotalol [RR = 0.91 (95% CI 0.08–10.65)] were associated with a relatively lower incidence of asthma attack than the placebo or control groups. However, the current evidence could not rule out the null hypothesis (Table 1 and Fig. 3A). The association of the beta-blocking agents and the incidence of asthma attack were ranked according to the SUCRA. In brief, oral atenolol was associated with the least risk of incidence of asthma attack after receiving beta-blocking agents, followed by oral celiprolol and oral bisoprolol (eTable 6A). In general, there was no detected significant heterogeneity (eTable 7). A meta-regression using restricted maximum likelihood estimators was performed to examine the potential effect of age and gender distribution on the incidence of asthma attack. The results of this meta-regression did not reveal a significant effect on the incidence of asthma attack when using a moderating variable, including age and gender distribution.

**Table 1:** League table of association between asthma exacerbation and beta-blocking agent prescription: overall.

Ate	1.00 (0.02,47.85)			0.77 (0.18,3.29)			0.25 (0.03,2.23)	0.25 (0.03,2.23)				0.36 (0.05,2.71)	*0.38 (0.15,0.98)				*0.06 (0.00,0.97)		0.33 (0.02,7.25)	*0.05 (0.00,0.77)			
1.29 (0.13,12.95)	Cel	1.00 (0.02,46.05)	1.00 (0.02,46.05)									0.33 (0.02,7.32)	0.20 (0.01,3.90)										
1.11 (0.04,28.19)	0.86 (0.03,28.40)	Bis	1.00 (0.02,46.05)								0.32 (0.01,7.63)	0.33 (0.02,7.32)											
1.11 (0.04,28.20)	0.86 (0.03,28.41)	1.00 (0.02,49.14)	CPro									0.333 0.015 7.323											
0.51 (0.20,1.28)	0.39 (0.04,4.11)	0.46 (0.02,11.65)	0.46 (0.02,11.65)	Pla	1.00 (0.02,47.18)	0.91 (0.09,9.60)	0.67 (0.27,1.61)	0.24 (0.03,2.16)		0.51 (0.05,5.81)		0.56 (0.11,2.72)	*0.14 (0.02,0.82)	0.47 (0.02,9.26)		0.33 (0.01,7.81)	*0.06 (0.00,0.97)	0.33 (0.02,7.30)	0.18 (0.02,1.49)	*0.05 (0.00,0.77)	0.14 (0.01,2.53)	0.14 (0.01,2.73)	*0.10 (0.01,0.73)
0.64 (0.02,22.67)	0.49 (0.01,31.93)	0.57 (0.01,64.96)	0.57 (0.01,64.96)	1.25 (0.04,39.52)	iPac																0.14 (0.01,2.53)		0.08 (0.01,1.25)
0.56 (0.04,7.81)	0.43 (0.01,13.05)	0.51 (0.01,29.52)	0.51 (0.01,29.52)	1.10 (0.09,12.97)	0.88 (0.01,61.08)	iSot																	
0.45 (0.15,1.38)	0.35 (0.03,3.80)	0.41 (0.02,10.65)	0.41 (0.02,10.65)	0.88 (0.35,2.22)	0.71 (0.02,25.03)	0.80 (0.06,11.12)	Pin	1.00 (0.24,4.24)	1.00 (0.07,14.90)			0.54 (0.17,1.74)	0.56 (0.12,2.60)				0.35 (0.08,1.47)			0.28 (0.07,1.15)			
0.43 (0.11,1.62)	0.33 (0.03,3.88)	0.39 (0.01,10.72)	0.39 (0.01,10.72)	0.85 (0.23,3.16)	0.67 (0.02,27.03)	0.77 (0.05,12.53)	0.96 (0.25,3.66)	Ace		0.33 (0.02,7.58)		0.54 (0.17,1.74)	0.56 (0.12,2.60)				0.35 (0.08,1.47)			0.28 (0.07,1.15)			
0.45 (0.02,9.14)	0.35 (0.01,13.74)	0.41 (0.01,29.85)	0.41 (0.01,29.85)	0.88 (0.05,16.73)	0.71 (0.01,65.56)	0.80 (0.02,37.14)	1.00 (0.06,16.33)	1.04 (0.05,23.15)	Cat														
0.43 (0.04,4.69)	0.33 (0.01,8.12)	0.39 (0.01,19.03)	0.39 (0.01,19.03)	0.84 (0.09,8.24)	0.67 (0.01,41.93)	0.76 (0.03,21.91)	0.95 (0.09,10.46)	0.99 (0.09,10.58)	0.95 (0.02,37.73)	Pra													
0.36 (0.00,34.97)	0.28 (0.00,32.72)	0.32 (0.01,8.27)	0.32 (0.00,51.31)	0.70 (0.01,68.71)	0.56 (0.00,173.48)	0.64 (0.00,115.86)	0.80 (0.01,79.57)	0.83 (0.01,86.16)	0.80 (0.00,173.76)	0.84 (0.01,133.70)	Car												
*0.35 (0.14,0.88)	0.27 (0.03,2.50)	0.32 (0.01,7.19)	0.32 (0.01,7.19)	0.69 (0.29,1.68)	0.55 (0.02,19.38)	0.63 (0.05,8.60)	0.78 (0.29,2.12)	0.82 (0.26,2.58)	0.78 (0.04,15.19)	0.82 (0.08,8.64)	0.98 (0.01,88.32)	Pro	1.20 (0.55,2.61)				0.69 (0.34,1.42)			0.56 (0.29,1.07)			
*0.34 (0.14,0.82)	0.26 (0.03,2.42)	0.31 (0.01,7.45)	0.31 (0.01,7.45)	0.67 (0.24,1.87)	0.53 (0.01,19.42)	0.60 (0.04,8.74)	0.75 (0.25,2.31)	0.79 (0.23,2.74)	0.75 (0.04,15.27)	0.79 (0.07,8.79)	0.95 (0.01,89.54)	0.96 (0.44,2.10)	Met		0.53 (0.05,5.29)		0.62 (0.28,1.38)		0.33 (0.01,7.63)	0.50 (0.24,1.05)			
0.24 (0.01,5.87)	0.18 (0.00,8.76)	0.21 (0.00,18.51)	0.21 (0.00,18.51)	0.47 (0.02,10.06)	0.37 (0.00,37.74)	0.42 (0.01,21.68)	0.53 (0.02,13.02)	0.55 (0.02,15.57)	0.53 (0.01,37.06)	0.56 (0.01,25.49)	0.66 (0.00,164.21)	0.67 (0.03,16.47)	0.70 (0.03,17.88)	Nad									
0.18 (0.01,2.34)	0.14 (0.01,3.68)	0.16 (0.00,8.87)	0.16 (0.00,8.87)	0.36 (0.03,4.86)	0.28 (0.00,21.45)	0.32 (0.01,11.70)	0.40 (0.03,5.68)	0.42 (0.03,6.28)	0.40 (0.01,18.88)	0.42 (0.01,12.67)	0.50 (0.00,86.57)	0.51 (0.04,6.42)	0.53 (0.05,5.89)	0.76 (0.01,42.98)	Bev								
0.17 (0.01,4.91)	0.13 (0.00,7.14)	0.15 (0.00,14.84)	0.15 (0.00,14.84)	0.33 (0.01,8.48)	0.27 (0.00,30.13)	0.30 (0.01,17.64)	0.38 (0.01,10.90)	0.39 (0.01,12.96)	0.38 (0.00,29.87)	0.40 (0.01,20.82)	0.47 (0.00,128.82)	0.48 (0.02,13.80)	0.50 (0.02,14.94)	0.71 (0.01,61.84)	0.94 (0.01,60.10)	iTol							
*0.19 (0.06,0.59)	0.15 (0.01,1.51)	0.17 (0.01,4.34)	0.17 (0.01,4.34)	0.37 (0.11,1.24)	0.30 (0.01,11.42)	0.34 (0.02,5.23)	0.42 (0.12,1.41)	0.44 (0.12,1.58)	0.42 (0.02,8.83)	0.44 (0.04,5.18)	0.53 (0.01,51.47)	0.54 (0.22,1.32)	0.56 (0.22,1.44)	0.80 (0.03,21.57)	1.05 (0.08,13.82)	1.11 (0.04,35.24)	Oxp			0.80 (0.49,1.32)			
0.14 (0.01,2.12)	0.10 (0.00,3.45)	0.12 (0.00,7.70)	0.12 (0.00,7.70)	0.27 (0.02,3.53)	0.21 (0.01,6.57)	0.24 (0.01,8.59)	0.30 (0.02,4.69)	0.31 (0.02,5.75)	0.30 (0.01,15.14)	0.32 (0.01,9.99)	0.38 (0.00,72.84)	0.39 (0.02,5.94)	0.40 (0.02,6.50)	0.57 (0.01,31.62)	0.75 (0.02,29.73)	0.80 (0.01,50.30)	0.72 (0.04,12.50)	iEsm					0.38 (0.06,2.28)
*0.13 (0.02,0.75)	0.10 (0.01,1.65)	0.12 (0.00,4.27)	0.12 (0.00,4.28)	0.26 (0.05,1.41)	0.21 (0.00,9.65)	0.24 (0.01,4.67)	0.29 (0.05,1.88)	0.31 (0.04,2.33)	0.29 (0.01,8.41)	0.31 (0.02,5.12)	0.37 (0.00,46.14)	0.38 (0.06,2.23)	0.39 (0.07,2.32)	0.56 (0.02,18.52)	0.73 (0.04,14.55)	0.78 (0.02,30.00)	0.70 (0.10,4.79)	0.98 (0.04,21.41)	Lab				
*0.15 (0.05,0.46)	0.12 (0.01,1.19)	0.14 (0.01,3.44)	0.14 (0.01,3.44)	*0.30 (0.09,0.96)	0.24 (0.01,9.06)	0.27 (0.02,4.14)	0.34 (0.10,1.10)	0.35 (0.10,1.23)	0.34 (0.02,6.99)	0.35 (0.03,4.09)	0.42 (0.00,40.95)	0.43 (0.18,1.01)	0.45 (0.18,1.11)	0.64 (0.02,17.10)	0.84 (0.06,10.93)	0.89 (0.03,27.96)	0.80 (0.34,1.91)	1.12 (0.07,19.21)	1.15 (0.17,7.70)	Tim			
0.09 (0.01,1.09)	0.07 (0.00,1.89)	0.08 (0.00,4.35)	0.08 (0.00,4.35)	0.18 (0.02,1.80)	0.14 (0.01,2.76)	0.16 (0.01,4.75)	0.20 (0.02,2.42)	0.21 (0.01,3.00)	0.20 (0.00,8.49)	0.21 (0.01,5.47)	0.25 (0.00,42.75)	0.26 (0.02,3.05)	0.27 (0.02,3.35)	0.38 (0.01,17.87)	0.50 (0.02,16.41)	0.54 (0.01,28.59)	0.48 (0.04,6.48)	0.67 (0.07,6.55)	0.69 (0.04,12.00)	0.60 (0.05,7.95)	iPLab		0.54 (0.18,1.59)
0.07 (0.00,1.74)	0.06 (0.00,2.61)	0.07 (0.00,5.53)	0.07 (0.00,5.53)	0.14 (0.01,2.97)	0.11 (0.00,11.28)	0.13 (0.00,6.45)	0.16 (0.01,3.85)	0.17 (0.01,4.61)	0.16 (0.00,11.05)	0.17 (0.00,7.58)	0.20 (0.00,49.27)	0.21 (0.01,4.87)	0.21 (0.01,5.29)	0.31 (0.00,22.95)	0.40 (0.01,22.07)	0.43 (0.01,36.20)	0.38 (0.01,10.07)	0.54 (0.01,28.86)	0.55 (0.02,17.71)	0.48 (0.02,12.40)	0.80 (0.02,36.07)	Sot	
*0.05 (0.01,0.48)	*0.04 (0.00,0.88)	0.04 (0.00,2.09)	0.04 (0.00,2.09)	*0.10 (0.01,0.77)	0.08 (0.00,1.38)	0.09 (0.00,2.22)	0.11 (0.01,1.07)	0.12 (0.01,1.34)	0.11 (0.00,4.04)	0.12 (0.01,2.54)	0.14 (0.00,21.16)	0.14 (0.01,1.34)	0.15 (0.01,1.48)	0.21 (0.01,8.51)	0.28 (0.01,7.73)	0.29 (0.01,13.69)	0.26 (0.02,2.89)	0.37 (0.06,2.46)	0.38 (0.03,5.44)	0.33 (0.03,3.54)	0.55 (0.15,1.99)	0.69 (0.02,26.99)	iPro

Pairwise (upper-right portion) and network (lower-left portion) meta-analysis results are presented as estimate effect sizes for the outcome of asthma exacerbation incidence rate. Interventions are reported in order of mean ranking of incidence of asthma exacerbation, and outcomes are expressed as odds ratio (OR) (95% confidence intervals). For the pairwise meta-analyses, OR of less than 1 indicate that the treatment specified in the row got less incidence of asthma exacerbation than that specified in the column. For the network meta-analysis (NMA), OR of less than 1 indicate that the treatment specified in the column got less incidence of asthma exacerbation than that specified in the row. Bold results marked with * indicate statistical significance.

*Ace* oral acebutolol, *Ate* oral atenolol, *Bev* oral bevantolol, *Bis* oral bisoprolol, *Car* oral carvedilol, *Cat* oral carteolol, *Cel* oral celiprolol, *CI* confidence interval, *CPro* oral celiprolol and propranolol, *ES* effect size, *iEsm* infusion of esmolol, *iPac* infusion of practolol, *iPLab* infusion of propranolol and labetalol, *iPro* infusion of propranolol, *iSot* infusion of sotalol, *iTol* infusion of tolamolol, *Lab* oral dilevalol or oral labetalol, *Met* oral metoprolol, *Nad* oral nadolol, *NMA* network meta-analysis, *OR* odds ratio, *Oxp* oral oxprenolol, *Pin* oral pindolol, *Pla* Placebo/control, *Pra* oral practolol, *Pro* oral propranolol, *Sot* oral sotalol, *SUCRA* surface under the cumulative ranking curve,* Tim* oral timolol.

#### Sensitivity test

After the removal of trials with zero event in their treatment arms, there were 11 remaining studies for the NMA (eFigure 1), which compared 10 treatments, including placebo, oral propranolol, oral pindolol, oral atenolol, oral acebutolol, oral bevantolol, oral metoprolol, oral carteolol, oral oxprenolol, and infusion of sotalol. The primary results of the NMA remained largely unchanged, except that oral atenolol was associated with a significantly lower incidence of asthma attack [RR = 0.33 (95% CI 0.14–0.74)] than the placebo or control groups (Table 2, eFigure 2 and eTable 6C).

**Table 2:** League table of association between asthma exacerbation and beta-blocking agent prescription: sensitivity test of removal of zero event.

Ate		0.46 (0.15,1.48)					*0.31 (0.12,0.82)		
0.74 (0.15,3.71)	Ace	0.45 (0.07,3.00)	0.44 (0.10,1.85)			1.00 (0.16,6.14)			0.27 (0.04,1.61)
0.43 (0.17,1.04)	0.58 (0.13,2.57)	Met	0.82 (0.37,1.83)			0.45 (0.07,3.00)		0.53 (0.05,5.29)	0.59 (0.26,1.36)
*0.37 (0.15,0.90)	0.50 (0.12,2.03)	0.86 (0.42,1.76)	Pro			0.44 (0.10,1.85)	0.77 (0.41,1.45)		0.67 (0.32,1.39)
0.36 (0.02,6.60)	0.48 (0.02,10.96)	0.84 (0.05,15.14)	0.97 (0.06,16.68)	Cat		1.00 (0.07,14.90)			
0.36 (0.03,4.39)	0.49 (0.03,7.91)	0.85 (0.07,10.28)	0.98 (0.09,11.14)	1.01 (0.03,39.75)	iSot		0.91 (0.09,9.60)		
0.36 (0.12,1.07)	0.48 (0.10,2.31)	0.84 (0.30,2.37)	0.97 (0.41,2.34)	1.00 (0.07,14.90)	0.99 (0.08,11.94)	Pin	1.33 (0.53,3.36)		0.27 (0.04,1.61)
*0.33 (0.14,0.74)	0.44 (0.10,1.94)	0.77 (0.34,1.74)	0.89 (0.51,1.57)	0.91 (0.05,15.27)	0.91 (0.09,9.60)	0.91 (0.41,2.02)	Pla		
0.23 (0.02,2.66)	0.31 (0.02,4.75)	0.53 (0.05,5.29)	0.62 (0.06,6.87)	0.64 (0.02,25.59)	0.63 (0.02,18.74)	0.64 (0.05,7.90)	0.70 (0.06,7.95)	Bev	
*0.23 (0.08,0.63)	0.31 (0.07,1.31)	0.54 (0.26,1.14)	0.63 (0.32,1.22)	0.64 (0.04,11.52)	0.64 (0.05,7.75)	0.64 (0.23,1.77)	0.70 (0.31,1.59)	1.01 (0.09,11.28)	Oxp

Pairwise (upper-right portion) and network (lower-left portion) meta-analysis results are presented as estimate effect sizes for the outcome of asthma exacerbation incidence rate. Interventions are reported in order of mean ranking of incidence of asthma exacerbation, and outcomes are expressed as odds ratio (OR) (95% confidence intervals). For the pairwise meta-analyses, OR of less than 1 indicate that the treatment specified in the row got less incidence of asthma exacerbation than that specified in the column. For the network meta-analysis (NMA), OR of less than 1 indicate that the treatment specified in the column got less incidence of asthma exacerbation than that specified in the row. Bold results marked with * indicate statistical significance.

*Ace* oral acebutolol, *Ate* oral atenolol, *Bev* oral bevantolol, *Bis* oral bisoprolol, *Car* oral carvedilol, *Cat* oral carteolol, *Cel* oral celiprolol, *CI* confidence interval, *CPro* oral celiprolol and propranolol, *ES* effect size, *iEsm* infusion of esmolol, *iPac* infusion of practolol, *iPLab* infusion of propranolol and labetalol, *iPro* infusion of propranolol, *iSot* infusion of sotalol, *iTol* infusion of tolamolol, *Lab* oral dilevalol or oral labetalol, *Met* oral metoprolol, *Nad* oral nadolol, *NMA* network meta-analysis, *OR* odds ratio, *Oxp* oral oxprenolol, *Pin* oral pindolol, *Pla* Placebo/control, *Pra* oral practolol, *Pro* oral propranolol, *Sot* oral sotalol, *SUCRA* surface under the cumulative ranking curve,* Tim* oral timolol.

### Subgroup analysis of participants with a definite baseline history of asthma

A total of 13 RCTs provided evidence related to patients with a baseline asthma history and 18 treatment arms, including placebo or control, oral propranolol, oral pindolol, oral atenolol, oral acebutolol, oral metoprolol, oral practolol, oral oxprenolol, oral timolol, oral labetalol, oral bisoprolol, oral celiprolol, infusion of esmolol, infusion of propranolol, infusion of propranolol and labetalol, infusion of practolol, oral celiprolol and propranolol, and oral bevantolol (Fig. 2B and Table 3).

**Table 3:** League table of association between asthma exacerbation and beta-blocking agent prescription: patients with baseline asthma.

Pla		1.00 (0.02,47.38)	1.00 (0.02,47.18)	0.57 (0.12,2.68)			0.67 (0.27,1.61)	0.24 (0.03,2.16)	*0.14 (0.02,0.82)	0.49 (0.02,14.93)	0.33 (0.02,7.30)	0.14 (0.01,2.53)		*0.06 (0.00,0.97)	0.18 (0.02,1.49)	*0.05 (0.00,0.77)	*0.10 (0.01,0.73)
1.17 (0.11,12.02)	Cel			1.00 (0.02,47.85)	1.00 (0.02,46.05)	1.00 (0.02,46.05)			0.20 (0.01,3.90)	0.33 (0.02,7.32)							
1.39 (0.06,33.84)	1.19 (0.03,53.95)	Pra						0.33 (0.02,7.58)									
1.26 (0.04,37.06)	1.08 (0.02,65.52)	0.91 (0.01,94.63)	iPac									0.14 (0.01,2.53)					0.08 (0.00,1.25)
0.82 (0.26,2.58)	0.70 (0.07,6.78)	0.59 (0.02,15.24)	0.65 (0.02,23.05)	Ate			0.25 (0.03,2.23)	0.25 (0.03,2.23)	*0.38 (0.15,0.98)	0.36 (0.05,2.71)				*0.06 (0.00,0.97)		*0.05 (0.00,0.77)	
0.95 (0.04,23.51)	0.82 (0.03,25.23)	0.68 (0.01,55.57)	0.75 (0.01,79.63)	1.16 (0.05,28.16)	Bis	1.00 (0.02,46.05)				0.33 (0.02,7.32)							
0.95 (0.04,23.51)	0.82 (0.03,25.23)	0.68 (0.01,55.57)	0.75 (0.01,79.63)	1.16 (0.05,28.16)	1.00 (0.02,46.05)	CPro				0.33 (0.02,7.32)							
0.65 (0.29,1.45)	0.55 (0.05,5.63)	0.46 (0.02,11.46)	0.51 (0.02,16.54)	0.79 (0.24,2.55)	0.68 (0.03,16.52)	0.68 (0.03,16.52)	Pin	1.00 (0.24,4.24)	0.56 (0.12,2.60)	0.54 (0.17,1.74)				0.35 (0.08,1.47)		0.28 (0.07,1.15)	
0.51 (0.14,1.87)	0.44 (0.04,4.78)	0.37 (0.02,7.81)	0.41 (0.01,15.20)	0.63 (0.16,2.39)	0.54 (0.02,13.73)	0.54 (0.02,13.73)	0.80 (0.23,2.81)	Ace	0.56 (0.12,2.60)	0.54 (0.17,1.74)				0.35 (0.08,1.47)		0.28 (0.07,1.15)	
*0.33 (0.12,0.92)	0.28 (0.03,2.45)	0.24 (0.01,5.79)	0.26 (0.01,8.95)	*0.40 (0.17,0.95)	0.35 (0.02,7.77)	0.35 (0.02,7.77)	0.51 (0.19,1.41)	0.64 (0.20,2.07)	Met	0.83 (0.38,1.80)			0.53 (0.05,5.29)	0.62 (0.28,1.38)	0.33 (0.01,7.63)	0.50 (0.24,1.05)	
*0.30 (0.11,0.84)	0.26 (0.03,2.21)	0.21 (0.01,5.15)	0.24 (0.01,8.10)	*0.36 (0.13,0.98)	0.31 (0.01,6.60)	0.31 (0.01,6.60)	0.46 (0.17,1.23)	0.58 (0.19,1.76)	0.90 (0.45,1.83)	Pro				0.69 (0.34,1.42)		0.56 (0.29,1.07)	
0.26 (0.02,3.20)	0.23 (0.01,6.87)	0.19 (0.00,10.90)	0.21 (0.01,5.66)	0.32 (0.02,5.02)	0.28 (0.00,16.11)	0.28 (0.00,16.11)	0.41 (0.03,5.62)	0.52 (0.03,8.51)	0.80 (0.05,11.83)	0.89 (0.06,13.14)	iEsm						0.38 (0.06,2.28)
0.18 (0.02,1.63)	0.15 (0.01,3.80)	0.13 (0.00,6.24)	0.14 (0.01,2.53)	0.22 (0.02,2.63)	0.19 (0.00,9.24)	0.19 (0.00,9.24)	0.28 (0.03,2.90)	0.35 (0.03,4.49)	0.55 (0.05,6.17)	0.60 (0.05,6.85)	0.68 (0.09,5.37)	iPLab					0.54 (0.18,1.59)
0.18 (0.01,2.17)	0.15 (0.01,3.52)	0.13 (0.00,6.46)	0.14 (0.00,9.42)	0.22 (0.02,2.49)	0.19 (0.00,8.82)	0.19 (0.00,8.82)	0.27 (0.02,3.35)	0.34 (0.03,4.50)	0.53 (0.05,5.29)	0.59 (0.05,6.51)	0.67 (0.02,22.87)	0.98 (0.03,27.58)	Bev				
*0.19 (0.07,0.55)	0.17 (0.02,1.51)	0.14 (0.01,3.37)	0.15 (0.00,5.30)	*0.24 (0.09,0.65)	0.20 (0.01,4.57)	0.20 (0.01,4.57)	*0.30 (0.11,0.82)	0.38 (0.12,1.16)	0.59 (0.29,1.19)	0.65 (0.34,1.26)	0.73 (0.05,10.92)	1.08 (0.09,12.31)	1.10 (0.10,12.16)	Oxp		0.80 (0.49,1.32)	
*0.15 (0.02,0.99)	0.13 (0.01,2.37)	0.11 (0.00,4.25)	0.12 (0.00,5.75)	0.19 (0.02,1.50)	0.16 (0.00,6.14)	0.16 (0.00,6.14)	0.23 (0.03,1.73)	0.29 (0.03,2.65)	0.46 (0.06,3.38)	0.51 (0.07,3.88)	0.57 (0.03,12.97)	0.84 (0.05,15.20)	0.86 (0.04,18.01)	0.78 (0.10,6.02)	Lab		
*0.16 (0.06,0.43)	0.13 (0.01,1.19)	0.11 (0.00,2.67)	0.12 (0.00,4.20)	*0.19 (0.07,0.50)	0.16 (0.01,3.62)	0.16 (0.01,3.62)	*0.24 (0.09,0.63)	*0.30 (0.10,0.90)	*0.47 (0.25,0.90)	*0.52 (0.29,0.95)	0.59 (0.04,8.64)	0.86 (0.08,9.72)	0.88 (0.08,9.60)	0.80 (0.49,1.32)	1.03 (0.14,7.77)	Tim	
*0.10 (0.01,0.73)	0.08 (0.00,1.81)	0.07 (0.00,3.05)	0.08 (0.00,1.26)	0.12 (0.01,1.21)	0.10 (0.00,4.51)	0.10 (0.00,4.51)	0.15 (0.02,1.32)	0.19 (0.02,2.07)	0.30 (0.03,2.81)	0.33 (0.03,3.13)	0.37 (0.06,2.18)	0.54 (0.18,1.60)	0.56 (0.02,13.83)	0.51 (0.05,4.85)	0.65 (0.04,10.11)	0.63 (0.07,5.94)	iPro

Pairwise (upper-right portion) and network (lower-left portion) meta-analysis results are presented as estimate effect sizes for the outcome of asthma exacerbation incidence rate. Interventions are reported in order of mean ranking of incidence of asthma exacerbation, and outcomes are expressed as odds ratio (OR) (95% confidence intervals). For the pairwise meta-analyses, OR of less than 1 indicate that the treatment specified in the row got less incidence of asthma exacerbation than that specified in the column. For the network meta-analysis (NMA), OR of less than 1 indicate that the treatment specified in the column got less incidence of asthma exacerbation than that specified in the row. Bold results marked with * indicate statistical significance.

*Ace* oral acebutolol, *Ate* oral atenolol, *Bev* oral bevantolol, *Bis* oral bisoprolol, *Car* oral carvedilol, *Cat* oral carteolol, *Cel* oral celiprolol, *CI* confidence interval, *CPro* oral celiprolol and propranolol, *ES* effect size, *iEsm* infusion of esmolol, *iPac* infusion of practolol, *iPLab* infusion of propranolol and labetalol, *iPro* infusion of propranolol, *iSot* infusion of sotalol, *iTol* infusion of tolamolol, *Lab* oral dilevalol or oral labetalol, *Met* oral metoprolol, *Nad* oral nadolol, *NMA* network meta-analysis, *OR* odds ratio, *Oxp* oral oxprenolol, *Pin* oral pindolol, *Pla* Placebo/control, *Pra* oral practolol, *Pro* oral propranolol, *Sot* oral sotalol, *SUCRA* surface under the cumulative ranking curve,* Tim* oral timolol.

In the subgroup NMA of patients with a baseline asthma history, besides oral timolol [RR = 6.42 (95% CI 2.34–17.61)] and infusion of propranolol [RR = 10.20 (95% CI 1.37–75.64)], there were additional beta-blockers that were associated with a significantly higher incidence of asthma attack than the placebo or control groups, including oral labetalol [RR = 6.60 (95% CI 1.01–43.29)], oral oxprenolol [RR = 5.15 (95% CI 1.81–14.69)], oral propranolol [RR = 3.35 (95% CI 1.20–9.38)], and oral metoprolol [RR = 3.03 (95% CI 1.09–8.41)]. In contrast, only oral practolol, infusion of practolol, and oral celiprolol retained their association with a relatively lower incidence of asthma attack than did the placebo or control groups, although not reaching statistical significance. The relative safety of oral bisoprolol, oral atenolol, oral celiprolol and propranolol, and infusion of sotalol did not persist in patients with a definite baseline asthma history. However, the current evidence could not rule out the null hypothesis (Table 3 and Fig. 3B). The association with the beta-blocking agents and the incidence of asthma attack in patients with a definite baseline history of asthma were ranked according to the SUCRA. In brief, the placebo or control group was associated with the least risk of incidence of asthma attack, followed by oral celiprolol and oral practolol (eTable 6B). A meta-regression was performed using restricted maximum likelihood estimators to analyze the potential effect of age and gender distribution on the incidence of asthma attack. The results of this meta-regression did not demonstrate a significant effect on the incidence of asthma attack when using a moderating variable, including age and gender distribution.

### Risk of bias and publication bias

We found that 43.4% (76/175 items), 49.7% (87/175 items), and 6.9% (12/175 items) of the included studies had an overall low, unclear, and high risk of bias, respectively. The ambigious results of randomization procedures or blindness of the studies further contributed to the potential bias (eFigures 3A,B).

Funnel plots of publication bias across the included studies (eFigures 4A–F) revealed a general symmetry, and the results of Egger’s test indicated no significant publication bias among the articles included in the NMA. In general, NMAs did not demonstrate inconsistency, in terms of either local inconsistency, as assessed using the loop-specific approach and the node-splitting method, or global inconsistency, as determined using the design-by-treatment method (eTables 8–9). In brief, the overall quality of evidence of the NMA, direct evidence, and indirect evidence were low to medium according to GRADE ratings (eTable 10).

## Discussion

To the best of our knowledge, this is the first NMA addressing the risk of asthma attack in conjunction with different beta-blocker treatments in the general and asthma population. Our findings suggest that across the entire sample, only oral timolol and infusion of propranolol were associated with a significantly higher risk of asthma attack than placebo, whereas the other beta-blockers such as oral celiprolol, oral celiprolol and propranolol, oral bisoprolol, oral atenolol, infusion of practolol, and infusion of sotalol exhibited a relative lower risk of asthma exacerbation than did placebo with no statistically significant differences. When focusing on participants with a baseline diagnosis of asthma, in addition to oral timolol and infusion of propranolol, oral labetalol, oxprenolol, propranolol, and metoprolol were also associated with a significantly higher incidence of asthma attack than placebo.

The major finding of the current NMA was that only oral timolol and infusion of propranolol were associated with a significantly higher risk of asthma attack than placebo, especially in participants with a baseline asthma diagnosis. The result that oral timolol [RR = 3.35 (95% CI 1.04–10.85)] had a substantially increased risk of developing an asthma attack was primarily reported in only one single-blind, randomized, crossover study that was included in our NMA^26^. The subjects showed a reduction in FEV_1_ of 53.3% from baseline after 2 h of 10 mg oral timolol. The serious adverse reaction of topical administration, ophthalmic timolol in glaucoma, has been discussed previously^27,28^. Oral propranolol is one nonselective beta-blocker, extensively used in the treatment of hypertension and ischemic heart disease due to its negative inotropic and chronotropic effects. Although chronic oral propranolol use in patients with asthma had no significant effect on airway hyper-responsiveness or no change in asthma control questionnaire (ACQ)^29^, intravenous infusion of propranolol resulted in marked symptomatic bronchoconstriction even at the lowest dose (1 mg)^30^. Therefore, oral timolol and infusion of propranolol definitively increase the risk of developing an asthma attack and are contraindicated for use in patients with asthma.

Although management of comorbidity in the primary care setting is the norm in modern medicine, clinical uncertainty still exists around whether to prescribe beta-blockers to people with asthma and cardiovascular disease. Timothy et al.^5^ reviewed seven studies and advised against the routine use of beta-blockers in patients with asthma and hypertension because of the increased adverse events of decline in FEV_1_ or asthma exacerbation. A retrospective cohort study using Veterans Administration databases in Iowa and Nebraska demonstrated that the hazard ratio of hospital admission for asthma was comparable for patients taking or not taking beta-blockers and that there was no difference between selective and nonselective beta-blockers^31^. Despite these observations, one population-based nested case–control study conducted in the UK demonstrated that nonselective beta-blockers were associated with a significantly increased risk of asthma exacerbation and that, in contrast, cardioselective beta-blocker exposure was not^15^. In our study, oral timolol and infusion of propranolol, both of which are nonselective beta-blockers, demonstrated a statistically significant risk for asthma exacerbation. Furthermore, oral labetalol, oxprenolol and propranolol, all nonselective, demonstrated a significantly higher incidence of asthma attack in patients with underlying asthma history. Therefore, these findings reveal that additional respiratory adverse events may be observed more consistently with nonselective beta-blocker use.

The effect of cardioselective beta-blockers on respiratory function was evaluated in two meta-analyses^6,7^. Patients with reactive airway disease who received one single dose of cardioselective beta-blockers had a 7.46% decrease in FEV_1_, an effect of 4.63% that was reversed by treatment with a beta-agonist inhaler. Patients who received continuous cardioselective beta-blockers experienced no significant reduction in FEV_1_, and no new symptoms developed. In addition, there were differences in the adverse effect on FEV_1_ with acute exposure of each of the other beta-blockers. Compared with placebo, celiprolol did not cause a change in FEV_1_, whereas metoprolol and atenolol did. In our study results, oral celiprolol, oral bisoprolol, oral atenolol and infusion of practolol showed a relatively lower risk of asthma exacerbation than did placebo without statistically significant differences. Moreover, the incidence of asthma attack was found to differ with cardioselective or nonselective beta-blockers according to the SUCRA in the current study. It is possible that the selectivity of beta_1_-adrenoceptor (calculated by beta 1-/beta 2-affinity ratios) varies, for example, from 13.5 for bisoprolol, 4.7 for atenolol, to 2.3 for metoprolol^32,33^. Celiprolol is a beta-blocker with a partial agonist activity and a greater selectivity than atenolol and bisoprolol. In our NMA, the sensitivity test revealed that oral atenolol demonstrated a significantly lower risk of asthma attack than did placebo, as studies on bisoprolol and celiprolol were removed due to no event being observed in their trials.

Although the Global Initiative for Asthma (GINA)^34^ guideline does not mention beta-blocker use in patients with asthma, other clinical guidelines for the treatment of asthma around the world provide various recommendations. The British Thoracic Society’s guideline recommends that all beta-blockers, including eye drops, be contraindicated^35^. However, the guideline of the National Heart, Lung, and Blood Institute in the USA recommends avoiding nonselective beta-blocker use in patients with asthma^36^. Correspondingly, guidelines from Australia^37^ and Japan^38^ suggest choosing cardioselective beta-blockers when possible. Our data support the additional recommendation that the use of the nonselective beta-blockers oral timolol and infusion of propranolol should be avoided. Furthermore, the cardioselective beta-blockers atenolol, bisoprolol, and celiprolol could be considered for use in patients with asthma and cardiovascular diseases.

There are several limitations that must be considered while interpreting our results. First, some of the analyses in this study were limited by underpowered statistics and small sample sizes, including heterogeneity in the characteristics of the participants (i.e., age, underlying diseases, initial severity of asthma, and trial duration) and the small numbers for some treatment arms. Also, the most included RCTs were published between the 1980s and 2000s. Second, differences in the dosing schemes and the route of administration (i.e., oral versus intravenous) of medications across the included studies may limit the comparability of outcomes in the present NMA. Third, variability in the definition of acute asthma attack of the included studies, including symptoms of wheezing, dyspnea, and symptomatic bronchospasm, may also limit the comparability of outcomes in our study. Fourth, the wide range of treatment durations among the investigated medications may limit the interpretation of the current study results. Fifth, the connection of the overall network structure was weak, so some of the interventions did not have additional direct evidence to support the primary result of the current NMA (i.e., infusion of sotalol, infusion of tolamolol, oral bevantolol, oral carteolol, oral carvedilol, and oral nadolol). In addition, although the superiority of oral celiprolol and oral bisoprolol in terms of preventing asthma exacerbation was ranked as 2^nd^ and 3^rd^, respectively, according to SUCRA, the primary evidence was based only on a limited number of RCTs (i.e., two RCTs for oral celiprolol^39,40^ and another two RCTs for oral bisoprolol)^39,41^. Therefore, clinicians should be careful when applying the results of the current NMA in their clinical practice. Finally, although we tried to include as many RCTs as possible by including early RCTs in 1976, it is possible that some RCTs were missed because of the use of the keyword “asthma” in our search strategy.

## Conclusion

This study showed that oral timolol and infusion of propranolol were associated with a significant risk of developing asthma attacks in patients with or without asthma history. Alternatively, oral celiprolol, oral celiprolol and propranolol, oral bisoprolol, oral atenolol, infusion of practolol, and infusion of sotalol might be associated with a lower incidence of asthma exacerbation. However, as some of the intervention comparisons were based only on a limited number of RCTs, clinicians should select specific treatments with caution and avoid the “one-size-fits-all” treatment for all clinical conditions.

## Supplementary Information

Supplementary Information 1.

Supplementary Information 2.
