# Supplementary material for: Do beta-adrenergic blocking agents increase asthma exacerbation? A network meta-analysis of randomized controlled trials
Source: Sci Rep. 2021 Jan 11;11:452. doi: 10.1038/s41598-020-79837-3 (PMC7801657; doi:10.1038/s41598-020-79837-3)
Supplement: Supplementary file 1 — Supplementary Information 1. [file 41598_2020_79837_MOESM1_ESM.pptx]

## Slide 1
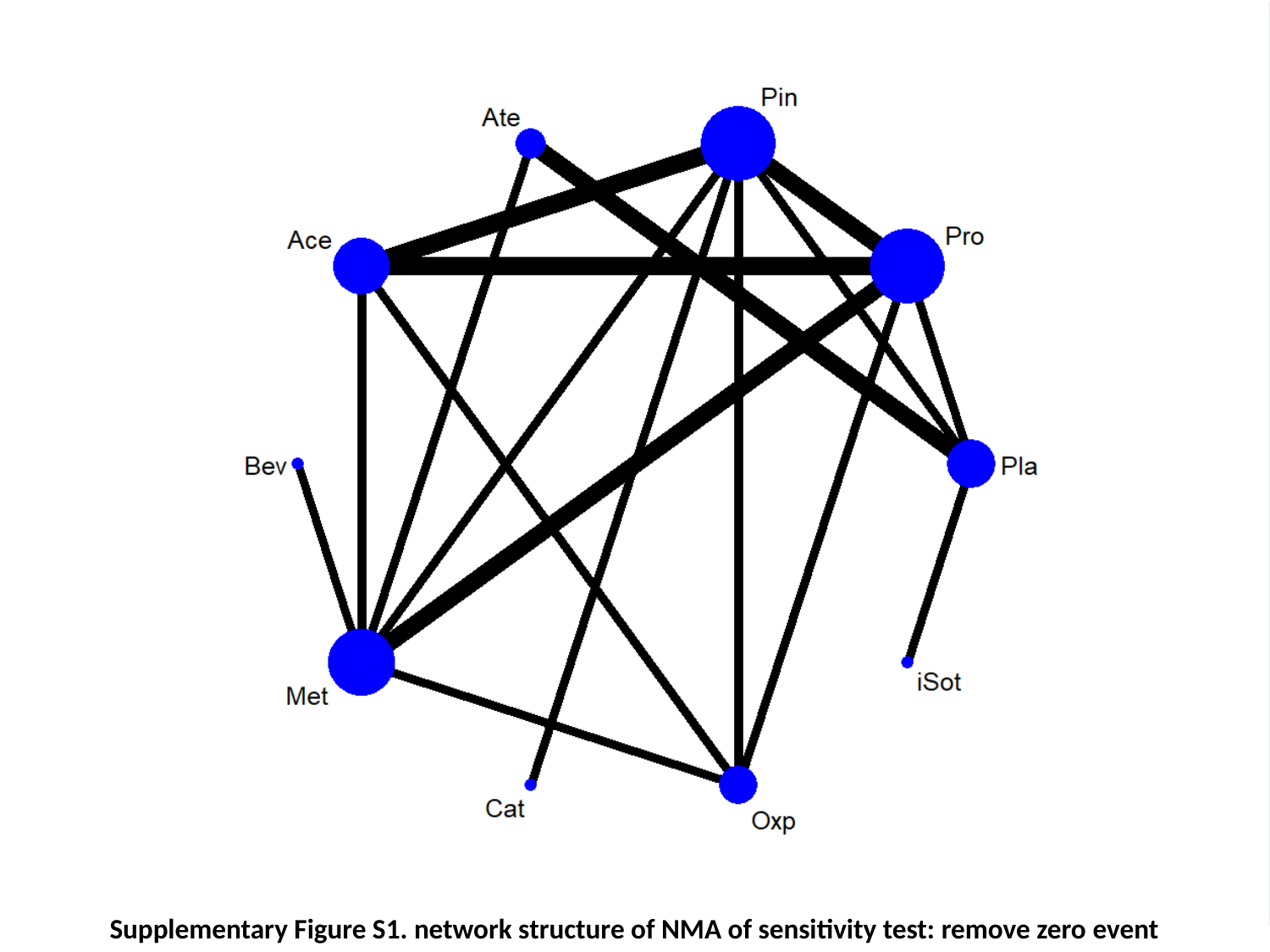

Supplementary Figure S1. network structure of NMA of sensitivity test: remove zero event

## Slide 2
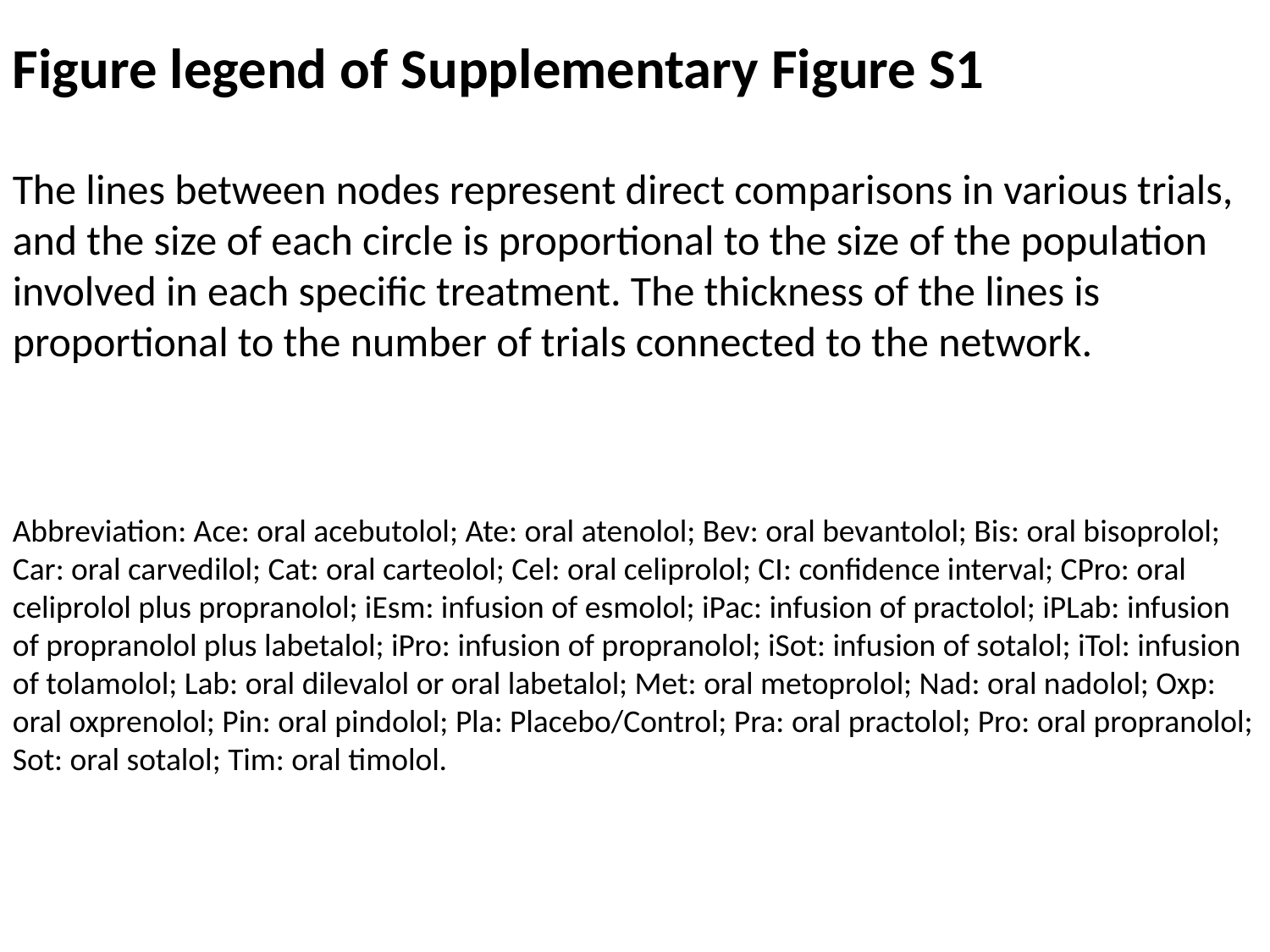

Figure legend of Supplementary Figure S1
The lines between nodes represent direct comparisons in various trials, and the size of each circle is proportional to the size of the population involved in each specific treatment. The thickness of the lines is proportional to the number of trials connected to the network.
Abbreviation: Ace: oral acebutolol; Ate: oral atenolol; Bev: oral bevantolol; Bis: oral bisoprolol; Car: oral carvedilol; Cat: oral carteolol; Cel: oral celiprolol; CI: confidence interval; CPro: oral celiprolol plus propranolol; iEsm: infusion of esmolol; iPac: infusion of practolol; iPLab: infusion of propranolol plus labetalol; iPro: infusion of propranolol; iSot: infusion of sotalol; iTol: infusion of tolamolol; Lab: oral dilevalol or oral labetalol; Met: oral metoprolol; Nad: oral nadolol; Oxp: oral oxprenolol; Pin: oral pindolol; Pla: Placebo/Control; Pra: oral practolol; Pro: oral propranolol; Sot: oral sotalol; Tim: oral timolol.

## Slide 3
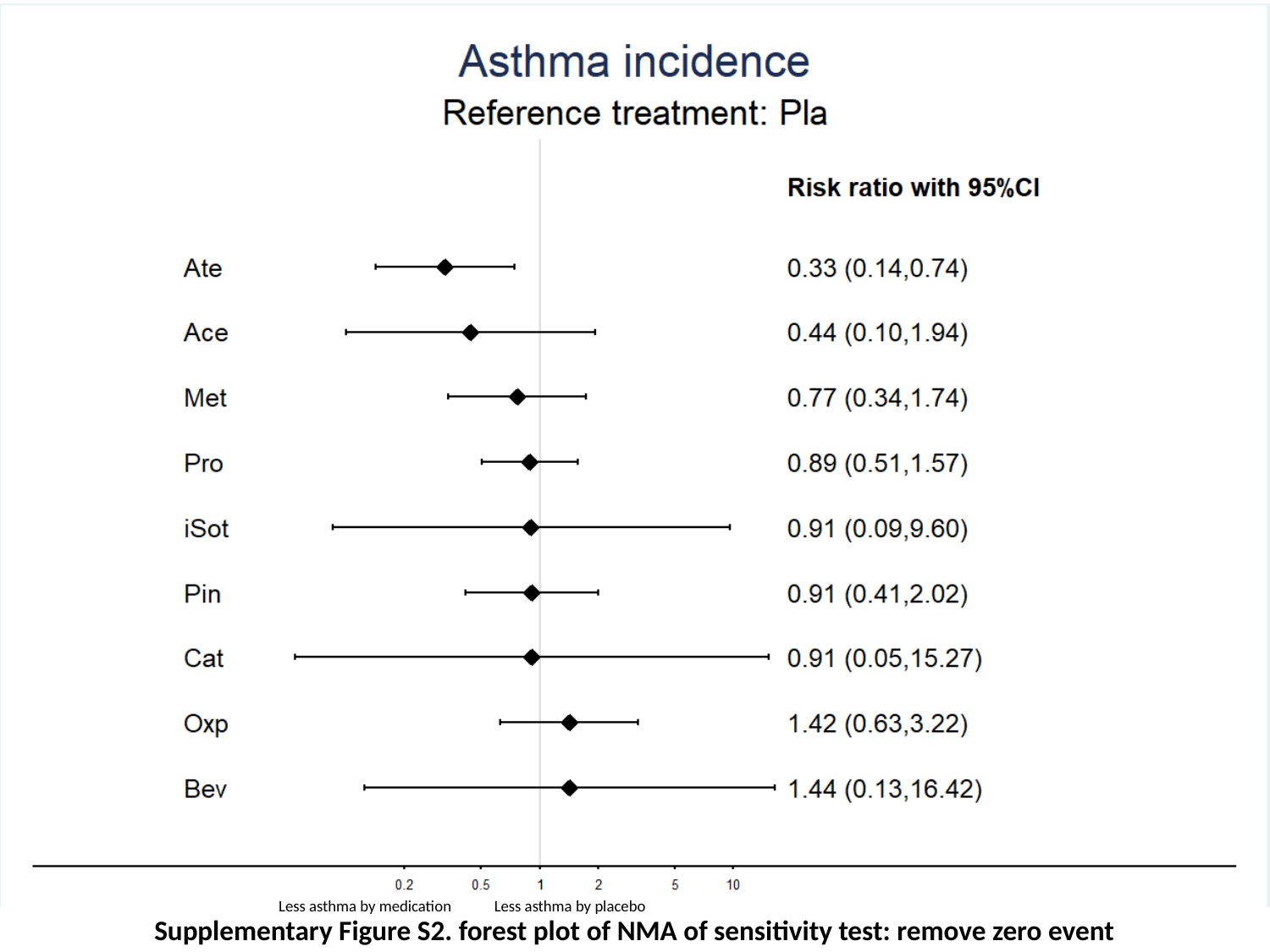

Less asthma by medication
Less asthma by placebo
Supplementary Figure S2. forest plot of NMA of sensitivity test: remove zero event

## Slide 4
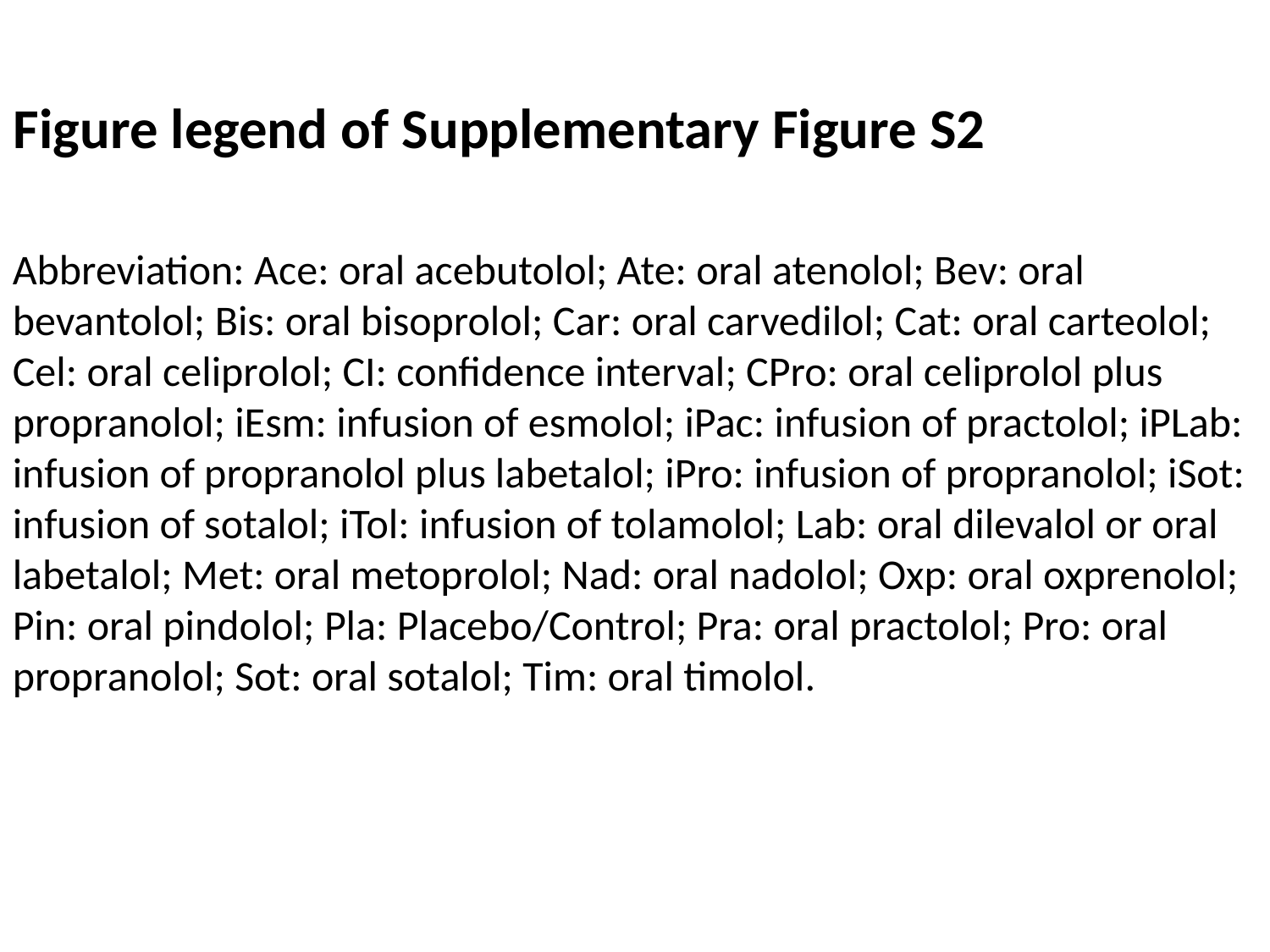

Figure legend of Supplementary Figure S2
Abbreviation: Ace: oral acebutolol; Ate: oral atenolol; Bev: oral bevantolol; Bis: oral bisoprolol; Car: oral carvedilol; Cat: oral carteolol; Cel: oral celiprolol; CI: confidence interval; CPro: oral celiprolol plus propranolol; iEsm: infusion of esmolol; iPac: infusion of practolol; iPLab: infusion of propranolol plus labetalol; iPro: infusion of propranolol; iSot: infusion of sotalol; iTol: infusion of tolamolol; Lab: oral dilevalol or oral labetalol; Met: oral metoprolol; Nad: oral nadolol; Oxp: oral oxprenolol; Pin: oral pindolol; Pla: Placebo/Control; Pra: oral practolol; Pro: oral propranolol; Sot: oral sotalol; Tim: oral timolol.

## Slide 5
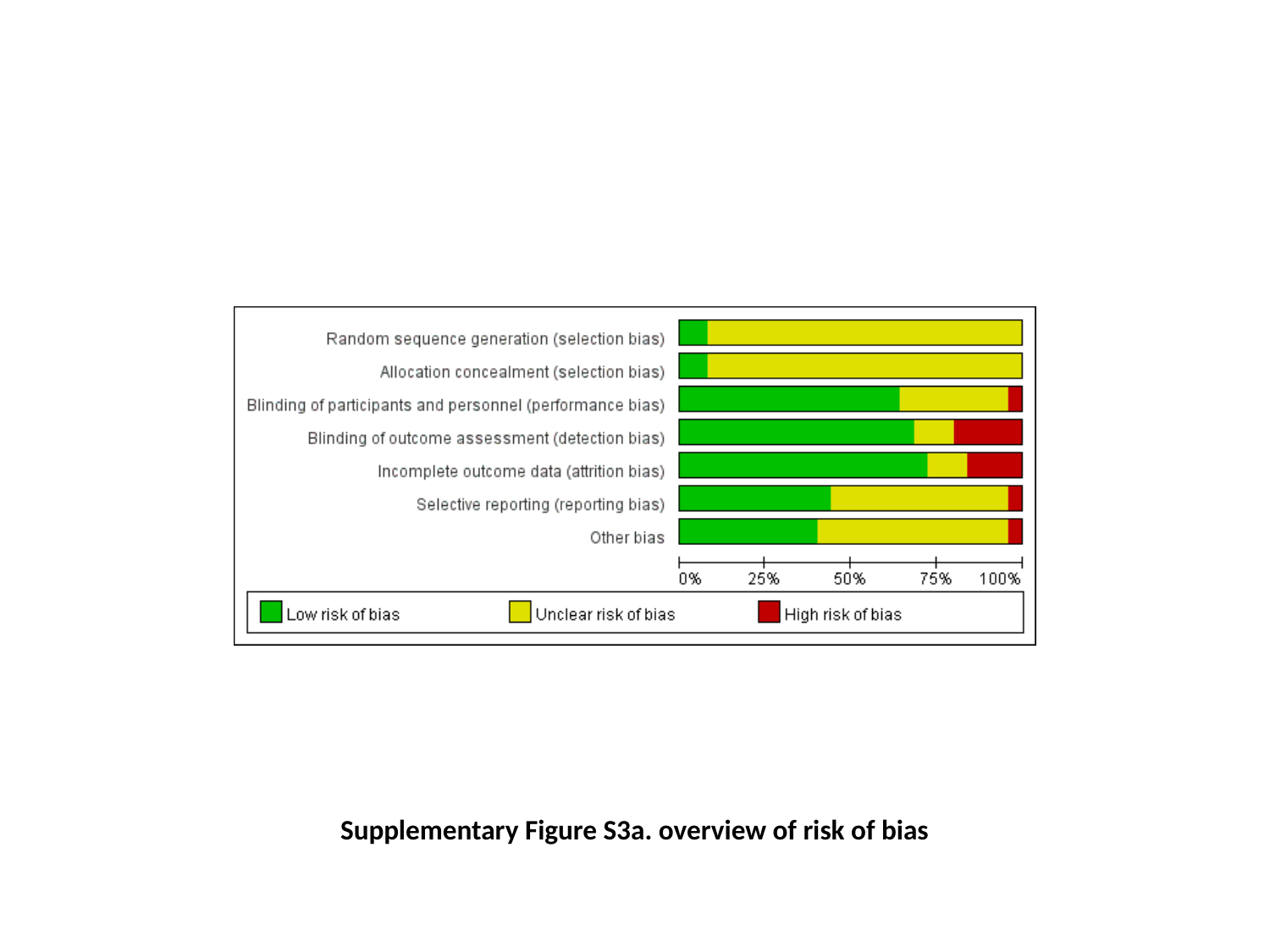

Supplementary Figure S3a. overview of risk of bias

## Slide 6
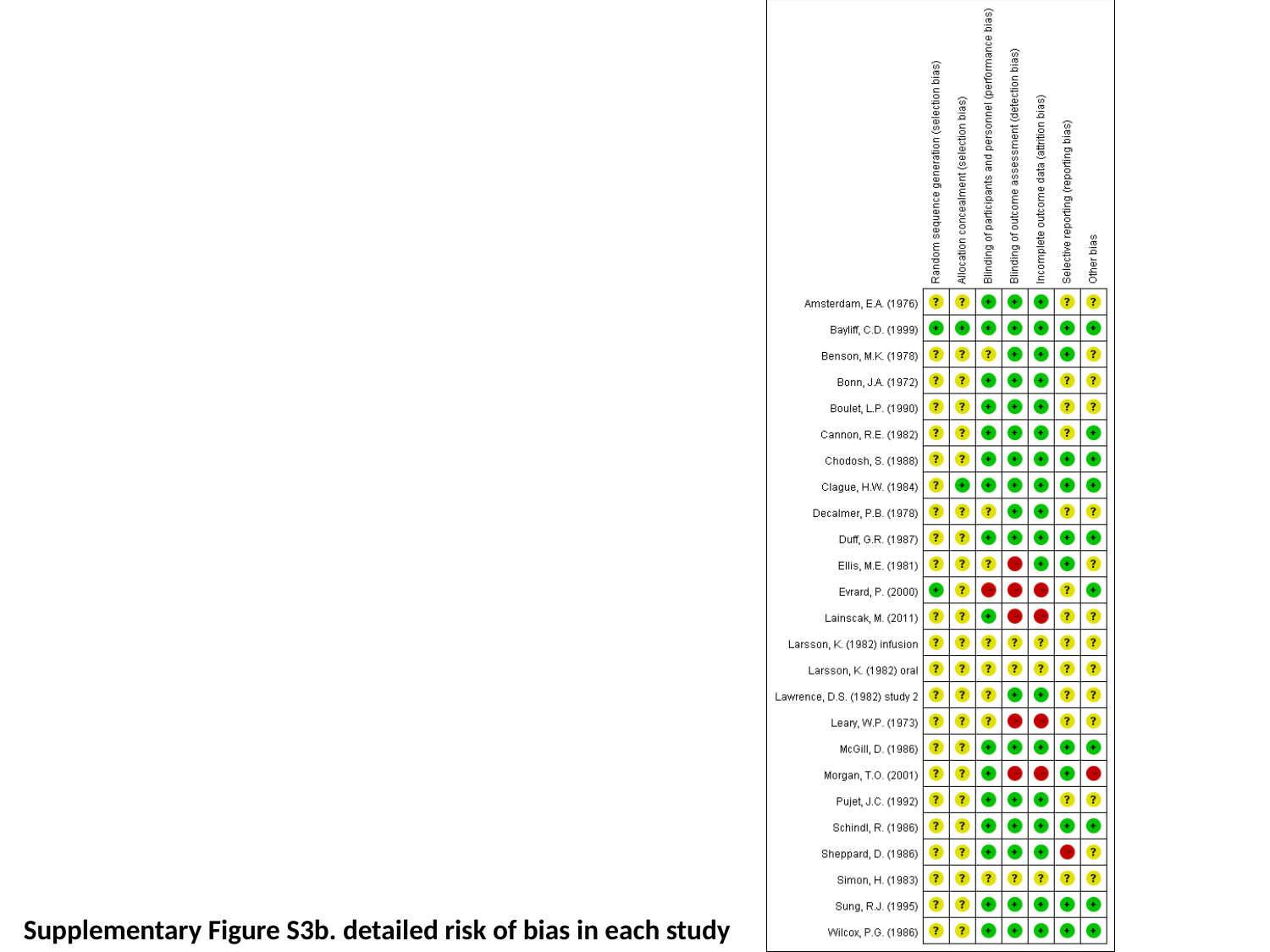

Supplementary Figure S3b. detailed risk of bias in each study

## Slide 7
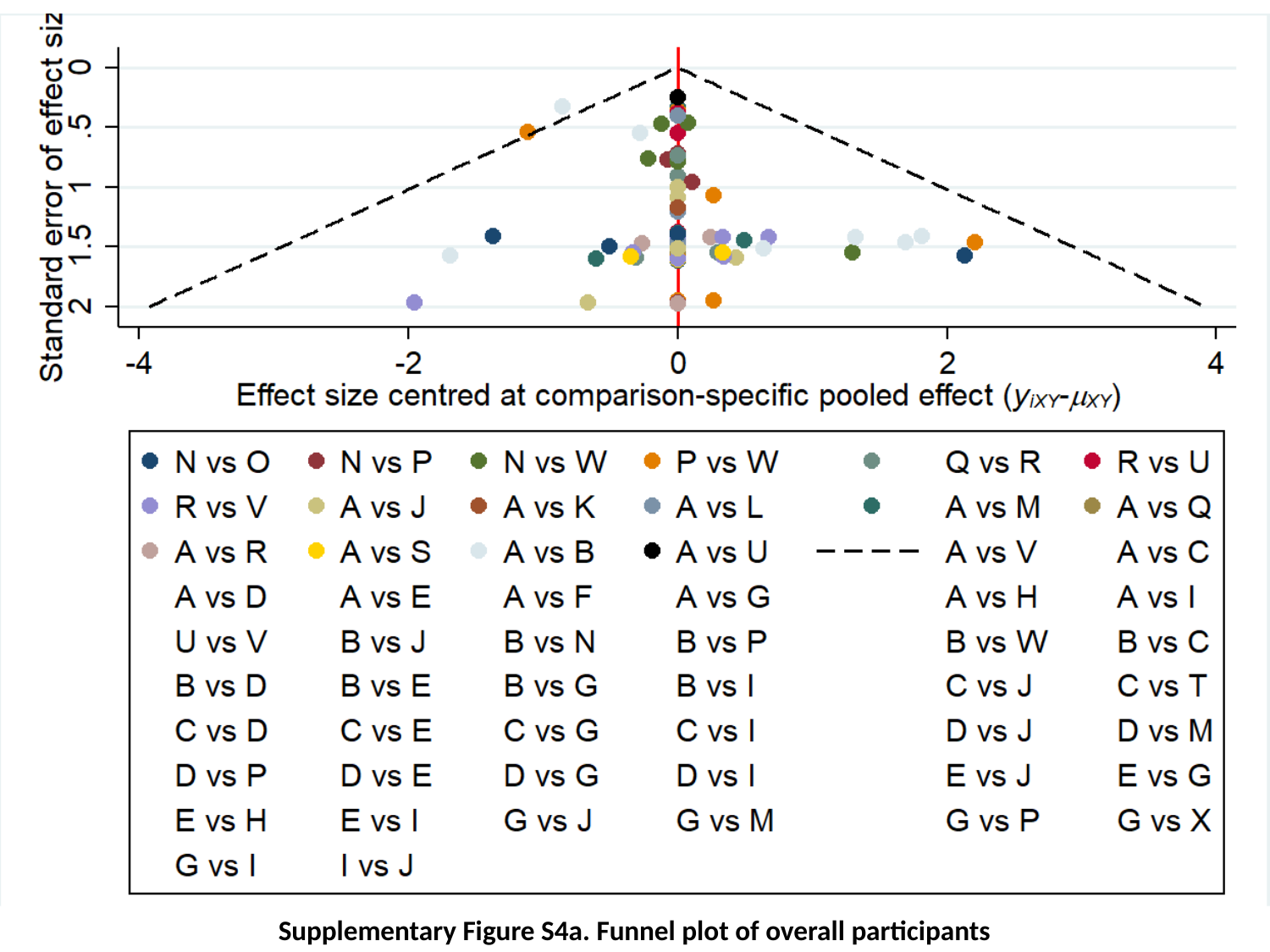

Supplementary Figure S4a. Funnel plot of overall participants

## Slide 8
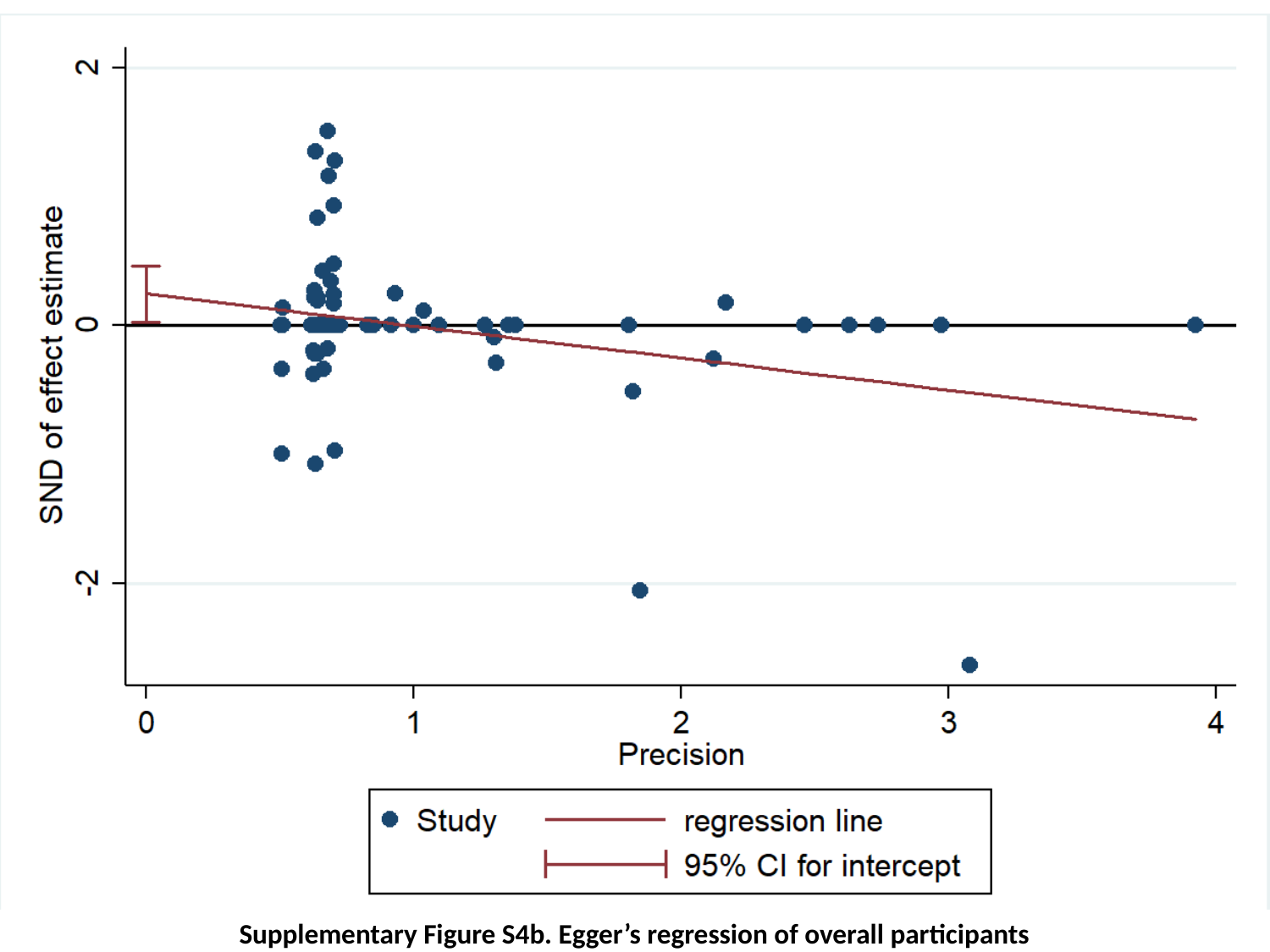

Supplementary Figure S4b. Egger’s regression of overall participants

## Slide 9
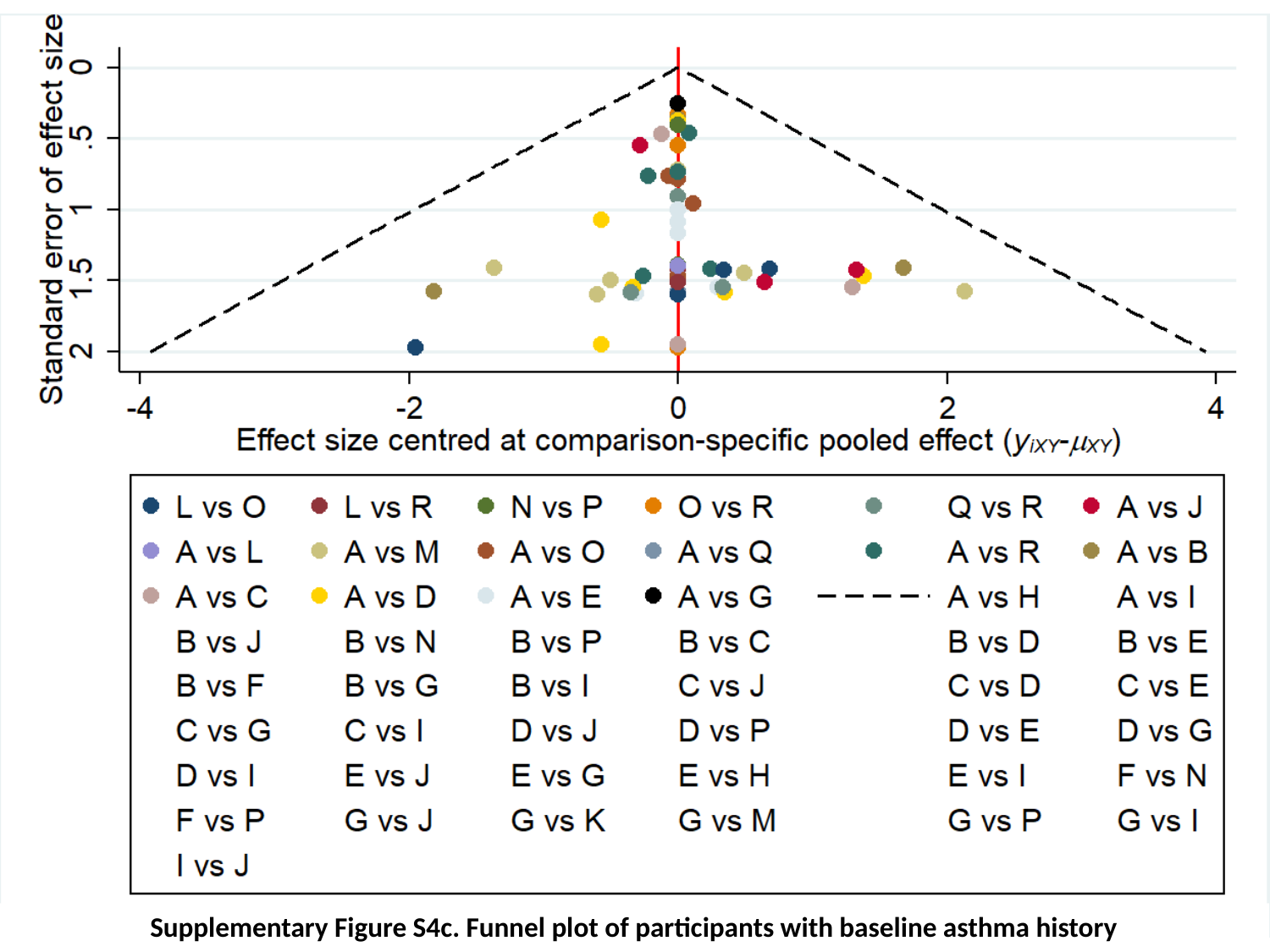

Supplementary Figure S4c. Funnel plot of participants with baseline asthma history

## Slide 10
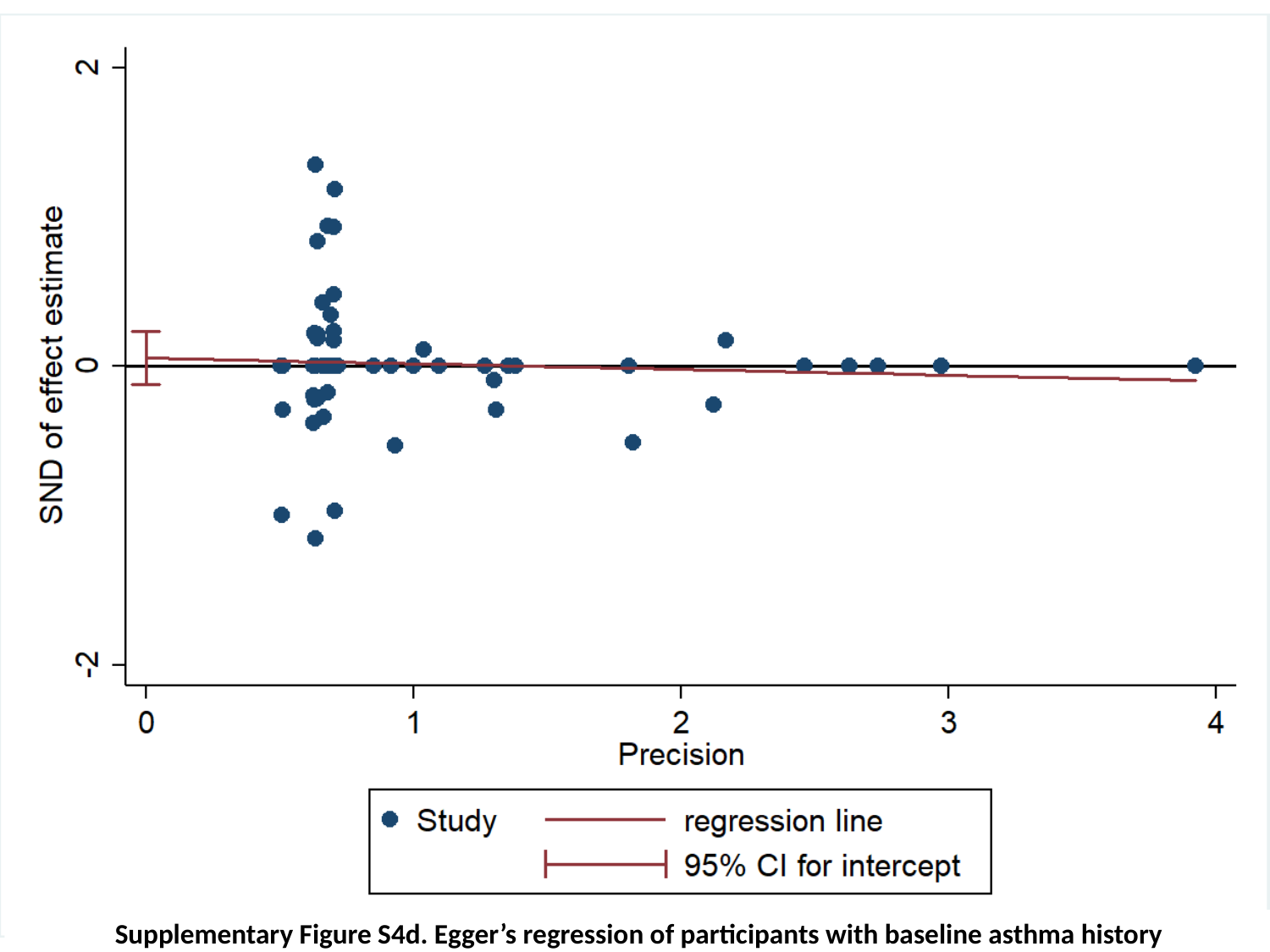

Supplementary Figure S4d. Egger’s regression of participants with baseline asthma history

## Slide 11
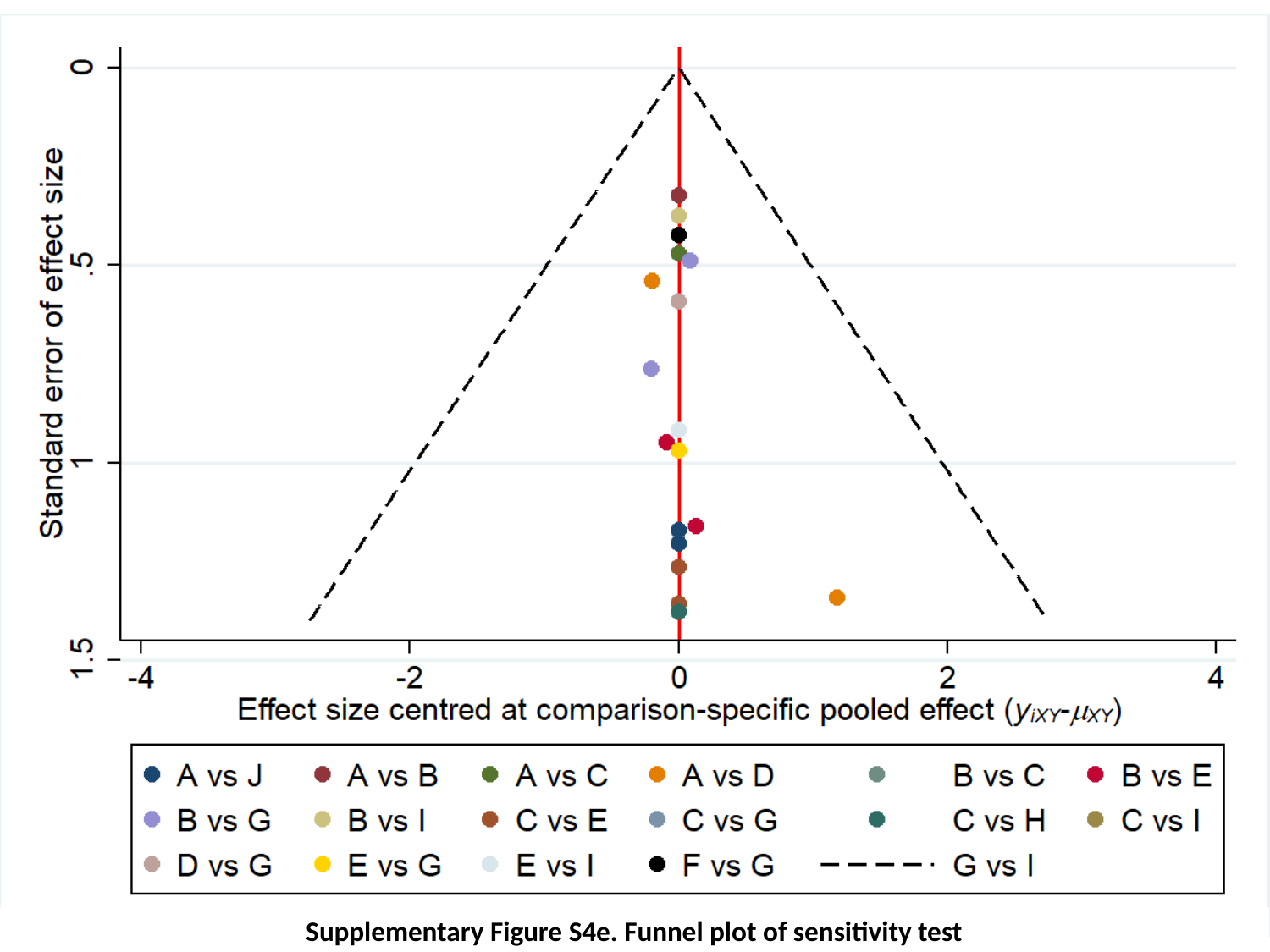

Supplementary Figure S4e. Funnel plot of sensitivity test

## Slide 12
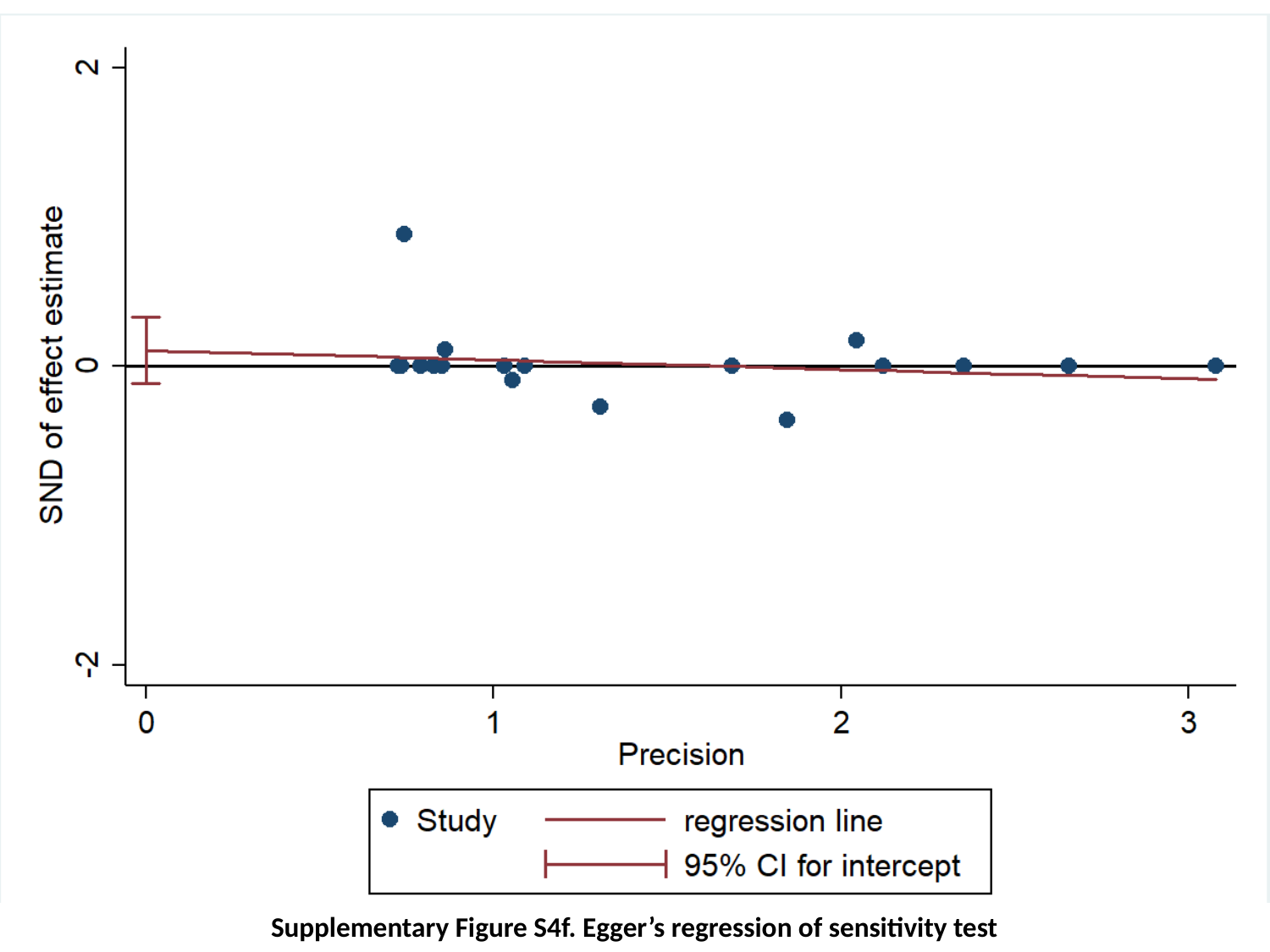

Supplementary Figure S4f. Egger’s regression of sensitivity test
