# Supplementary material for: Do beta-adrenergic blocking agents increase asthma exacerbation? A network meta-analysis of randomized controlled trials
Source: Sci Rep. 2021 Jan 11;11:452. doi: 10.1038/s41598-020-79837-3 (PMC7801657; doi:10.1038/s41598-020-79837-3)
Supplement: Supplementary file 2 — Supplementary Information 2. [file 41598_2020_79837_MOESM2_ESM.docx]

**Do beta-adrenergic blocking agents increase asthma exacerbation? A network meta-analysis of randomized controlled trials**

Kuo-Yang Huang, Ping-Tao Tseng, Yi-Cheng Wu, Yu-Kang Tu, Brendon Stubbs, Kuan-Pin Su, Yutaka J. Matsuoka, Chih-Wei Hsu, Ching‑Hsiung Lin, Yen-Wen Chen, Pao-Yen Lin

**eTable 1: eMethods**

*General guideline applied in the current study*

The current NMA was done according to the preferred reporting items for systematic reviews and meta-analyses extension guideline [1].

*Search strategy and selection criteria*

We conducted a systematic publication review using the ClinicalKey, ClinicalTrials.gov, Cochrane CENTRAL, Embase, ProQuest, PubMed, ScienceDirect, and Web of Science databases from inception to final update search on June 5^th^, 2020. No language restriction was put in place. Manual searches were also conducted for those potentially eligible articles selected from the reference lists of review articles or pairwise meta-analyses [2, 3].

*Inclusion and exclusion criteria*

We included only RCTs, with either placebo-controlled or active-controlled design, conducted in human beings with published articles. The targets of comparison arms were set to be the beta-blocking agents used in patients with or without baseline asthma history.

The exclusion criteria were (1) not clinical trials, (2) not RCTs, (3) not reporting the adverse event of asthma exacerbation, (4) not related to beta-blocking agents, or (5) not comparing beta-blocking agents or placebo. In cases of duplicated usage of data (i.e., different articles based on the same sample sources), we included only the article with the most informative and the largest sample source.

*Data extraction*

Two authors (KY Huang and PT Tseng) independently screened for the studies, extracted the relevant information from the manuscripts, and evaluated the risk of bias among the included studies. In cases of discrepancy, the corresponding author (PY Lin) was involved. If there was a lack of available data from the manuscripts, the corresponding authors or coauthors were contacted to obtain the original data. We followed the flowchart according to previous NMAs [4-7].

*Outcomes*

Because a decreased pulmonary function associated with beta-blocking agents would not always result in clinical symptoms, we did not choose the changes in pulmonary function as our primary outcome [8]. The primary outcome was the incidence of asthma attacks after treatment with beta-blocking agents compared with control conditions in patients with or without baseline asthma history. The definition of an asthma attack could be deterioration in symptoms, increased use of rescue bronchodilators, emergency room visits for asthma, and requiring systemic corticosteroids [9].

*Cochrane risk-of-bias tool*

Two independent authors (KY Huang and PT Tseng) evaluated the risk of bias (interrater reliability, 0.85) for each domain described in the Cochrane risk-of-bias tool [10]. Studies were then further classified into the overall risk-of-bias category.

*Statistical analysis*

The NMA was performed on the STATA version 16.0 (StataCorp LLC, College Station, TX, USA). We estimated the summary risk ratios (RRs) with 95% confidence intervals (CIs) for categorical variables and further applied a 0.5 zero-cell correction during the procedure of the meta-analysis. However, in case, in one study, there were zeroes in both the intervention and control arms, we did not apply such a correction procedure because of the risk of increasing the bias; instead, we removed such studies from our analysis [11, 12]. Furthermore, to minimize the potential bias caused by imputing 0.5 to zero-cells in the data, we conducted a sensitivity test by removing trials with zero event or 100% event in their treatment arms. We used the frequentist model of NMA to compare the effect sizes (ES) between studies with similar interventions. All comparisons were done using a two-tailed test, and a *p* value cutoff point of 0.05 was considered to be statistically significant. Heterogeneity among the included studies was evaluated using the tau value, which is the estimated standard deviation of the effect across the included studies.

Regarding the procedure of the meta-analysis applied in the present study, we used a mixed comparison with generalized linear mixed models to analyze the direct and indirect comparisons among the NMA [13]. To be specific, indirect comparisons could be conducted by transitivity, which indicated that the differences between treatments A and B could be calculated from their comparisons with the third treatment, C. To calculate the comparisons of multiple treatment arms, we combined the direct and indirect evidence from the included studies [14]. The direct evidence between two treatment arms (i.e., treatment A and treatment B) indicated that there had been a direct comparison between treatment A and treatment B in at least one of the included studies. On the other hand, the indirect evidence between two treatment arms (i.e., treatment A and treatment C) indicated that we had obtained the effect sizes between the comparison pairs of treatment A and treatment C by combining the effect sizes between the comparison pairs of treatment A and treatment B and the effect sizes between the comparison pairs of treatment B and treatment C. When we did not have direct comparisons between treatment A and treatment C in the included studies, for example, as shown in Figure 2A, there was no direct comparison between the placebo or control group and the bisoprolol group in the included studies, we obtained the indirect evidence between the placebo or control group and the bisoprolol group by comparing with the propranolol group. The STATA program was used in our NMA with the mvmeta command and self-programmed STATA [15]. The restricted maximum likelihood method was used to evaluate the between-study variance [16].

To provide additional clinical application, we calculated the relative ranking probabilities between the preventive effects of all treatments for the target outcomes. In brief, the surface under the cumulative ranking curve (SUCRA) indicates the percentage of the mean rank of each medication relative to an imaginary intervention that is the best without uncertainty [17]. When the area under the curve was larger, the treatment deserved a higher rank of an asthma attack incidence.

We conducted meta-regression to determine the relationship between the incidence of asthma and the characteristics of participants, such as the mean age and gender distribution. To evaluate the effect of potential factors, we selected studies to perform a subgroup analysis. To be specific, we arranged the subgroup analysis focusing on patients with a definite baseline asthma history.

Finally, we evaluated the potential inconsistency between the direct and indirect evidence within the network using the loop-specific approach and the local inconsistency using the node-splitting method. In addition, we used the design-by-treatment model to evaluate the global inconsistency among the entire NMA [18]. We evaluated the certainty of the evidence, including direct evidence, indirect evidence, and network meta-analysis evidence, according to the GRADE framework [19, 20].

**References:**

1. Hutton B, Salanti G, Caldwell DM, Chaimani A, Schmid CH, Cameron C, Ioannidis JP, Straus S, Thorlund K, Jansen JP *et al*: The PRISMA extension statement for reporting of systematic reviews incorporating network meta-analyses of health care interventions: checklist and explanations. Ann Intern Med. 2015; 162(11):777-84.

2. Morales DR, Jackson C, Lipworth BJ, Donnan PT, Guthrie B: Adverse respiratory effect of acute beta-blocker exposure in asthma: a systematic review and meta-analysis of randomized controlled trials. Chest. 2014; 145(4):779-86.

3. Salpeter S, Ormiston T, Salpeter E: Cardioselective beta-blockers for reversible airway disease. The Cochrane database of systematic reviews. 2002(4):CD002992.

4. Cipriani A, Furukawa TA, Salanti G, Chaimani A, Atkinson LZ, Ogawa Y, Leucht S, Ruhe HG, Turner EH, Higgins JPT *et al*: Comparative efficacy and acceptability of 21 antidepressant drugs for the acute treatment of adults with major depressive disorder: a systematic review and network meta-analysis. Lancet. 2018; 391(10128):1357-66.

5. Lai CC, Tu YK, Wang TG, Huang YT, Chien KL: Effects of resistance training, endurance training and whole-body vibration on lean body mass, muscle strength and physical performance in older people: a systematic review and network meta-analysis. Age and ageing. 2018; 47(3):367-73.

6. Salanti G, Del Giovane C, Chaimani A, Caldwell DM, Higgins JP: Evaluating the quality of evidence from a network meta-analysis. PloS one. 2014; 9(7):e99682.

7. Tu YK, Faggion CM, Jr.: A primer on network meta-analysis for dental research. ISRN Dent. 2012; 2012:276520.

8. Osborne ML, Vollmer WM, Pedula KL, Wilkins J, Buist AS, O'Hollaren M: Lack of correlation of symptoms with specialist-assessed long-term asthma severity. Chest. 1999; 115(1):85-91.

9. Reddel HK, Taylor DR, Bateman ED, Boulet LP, Boushey HA, Busse WW, Casale TB, Chanez P, Enright PL, Gibson PG *et al*: An official American Thoracic Society/European Respiratory Society statement: asthma control and exacerbations: standardizing endpoints for clinical asthma trials and clinical practice. Am J Respir Crit Care Med. 2009; 180(1):59-99.

10. Higgins J, Green S: Cochrane Handbook for Systematic Reviews of Interventions Version 5.0.2: The Cochrane Collaboration; 2009.

11. Cheng J, Pullenayegum E, Marshall JK, Iorio A, Thabane L: Impact of including or excluding both-armed zero-event studies on using standard meta-analysis methods for rare event outcome: a simulation study. BMJ Open. 2016; 6(8):e010983.

12. Brockhaus AC, Bender R, Skipka G: The Peto odds ratio viewed as a new effect measure. Stat Med. 2014; 33(28):4861-74.

13. Tu YK: Use of generalized linear mixed models for network meta-analysis. Medical decision making : an international journal of the Society for Medical Decision Making. 2014; 34(7):911-8.

14. Lu G, Ades AE: Combination of direct and indirect evidence in mixed treatment comparisons. Statistics in medicine. 2004; 23(20):3105-24.

15. White IR: Network meta-analysis. Stata Journal. 2015; 15(4):951-85.

16. Kontopantelis E, Springate DA, Reeves D: A re-analysis of the Cochrane Library data: the dangers of unobserved heterogeneity in meta-analyses. PloS one. 2013; 8(7):e69930.

17. Salanti G, Ades AE, Ioannidis JP: Graphical methods and numerical summaries for presenting results from multiple-treatment meta-analysis: an overview and tutorial. J Clin Epidemiol. 2011; 64(2):163-71.

18. Higgins JP, Del Giovane C, Chaimani A, Caldwell DM, Salanti G: Evaluating the Quality of Evidence from a Network Meta-Analysis. Value Health. 2014; 17(7):A324.

19. Puhan MA, Schunemann HJ, Murad MH, Li T, Brignardello-Petersen R, Singh JA, Kessels AG, Guyatt GH, Group GW: A GRADE Working Group approach for rating the quality of treatment effect estimates from network meta-analysis. Bmj. 2014; 349:g5630.

20. Schunemann H, Brozek J, Guyatt G, Oxman A: Handbook for grading the quality of evidence and the strength of recommendations using the GRADE approach: The Cochrane Collaboration GRADE Working Group; 2013.

**eTable 2: PRISMA checklist of current meta-analysis**

| **Section/Topic** | **#** | **Checklist Item** | **Reported on Page #** |
| --- | --- | --- | --- |
| **TITLE** | | | |
| Title | 1 | Identify the report as a systematic review incorporating a network meta-analysis (or related form of meta-analysis) | 1 |
| **ABSTRACT** | | | |
| Summary | 2 | Provide a structured summary including, as applicable: Background: main objectives Methods: data sources; study eligibility criteria, participants, and interventions; study appraisal; and synthesis methods, such as network meta-analysis. Results: number of studies and participants identified; summary estimates with corresponding confidence/credible intervals; treatment rankings may also be discussed. Authors may choose to summarize pairwise comparisons against a chosen treatment included in their analyses for brevity. Discussion/Conclusions: limitations; conclusions and implications of findings. Other: primary source of funding; systematic review registration number with registry name | 4 |
| **INTRODUCTION** | | | |
| Rationale | 3 | Describe the rationale for the review in the context of what is already known, including mention of why a network meta-analysis has been conducted | 5-6 |
| Objectives | 4 | Provide an explicit statement of questions being addressed, with reference to participants, interventions, comparisons, outcomes, and study design (PICOS) | 5-6 |
| **METHODS** | | | |
| Protocol and registration | 5 | Indicate whether a review protocol exists and if and where it can be accessed (e.g., Web address); and, if available, provide registration information, including registration number. | 7, eTable 1 |
| Eligibility criteria | 6 | Specify study characteristics (e.g., PICOS, length of follow-up) and report characteristics (e.g., years considered, language, publication status) used as criteria for eligibility, giving rationale. Clearly describe eligible treatments included in the treatment network, and note whether any have been clustered or merged into the same node (with justification). | 7, eTable 1 |
| Information sources | 7 | Describe all information sources (e.g., databases with dates of coverage, contact with study authors to identify additional studies) in the search and date last searched. | 7, eTable 1 |
| Search | 8 | Present full electronic search strategy for at least one database, including any limits used, such that it could be repeated | 7, eTable 1 |
| Study selection | 9 | State the process for selecting studies (i.e., screening, eligibility, included in systematic review, and, if applicable, included in the meta-analysis). | 7, eTable 1 |
| Data collection process | 10 | Describe method of data extraction from reports (e.g., piloted forms, independently, in duplicate) and any processes for obtaining and confirming data from investigators | 7, eTable 1 |
| Data items | 11 | List and define all variables for which data were sought (e.g., PICOS, funding sources) and any assumptions and simplifications made | 7, eTable 1 |
| Geometry of the network | S1 | Describe methods used to explore the geometry of the treatment network under study and potential biases related to it. This should include how the evidence base has been graphically summarized for presentation, and what characteristics were compiled and used to describe the evidence base to readers. | 7, eTable 1 |
| Risk of bias in individual studies | 12 | Describe methods used for assessing risk of bias of individual studies (including specification of whether this was done at the study or outcome level), and how this information is to be used in any data synthesis. | 7, eTable 1 |
| Summary measures | 13 | State the principal summary measures (e.g., risk ratio, difference in means). Also describe the use of additional summary measures assessed, such as treatment rankings and surface under the cumulative ranking curve (SUCRA) values, as well as modified approaches used to present summary findings from meta-analyses. | 7, eTable 1 |
| Planned methods of analysis | 14 | Describe the methods of handling data and combining results of studies for each network meta-analysis. This should include, but not be limited to: Handling of multigroup trials; Selection of variance structure; Selection of prior distributions in Bayesian analyses; and Assessment of model fit | 7, eTable 1 |
| Assessment of inconsistency | S2 | Describe the statistical methods used to evaluate the agreement of direct and indirect evidence in the treatment network(s) studied. Describe efforts taken to address its presence when found. | 7, eTable 1 |
| Risk of bias across studies | 15 | Specify any assessment of risk of bias that may affect the cumulative evidence (e.g., publication bias, selective reporting within studies). | 7, eTable 1 |
| Additional analyses | 16 | Describe methods of additional analyses if done, indicating which were prespecified. This may include, but not be limited to, the following: Sensitivity or subgroup analyses; Meta-regression analyses; Alternative formulations of the treatment network; and Use of alternative prior distributions for Bayesian analyses (if applicable). | 7, eTable 1 |
| **RESULTS** | | | |
| Study selection | 17 | Give numbers of studies screened, assessed for eligibility, and included in the review, with reasons for exclusions at each stage, ideally with a flow diagram | 8-9 |
| Presentation of network structure | S3 | Provide a network graph of the included studies to enable visualization of the geometry of the treatment network. | Figure 2A-B, eFigure 1 |
| Summary of network geometry | S4 | Provide a brief overview of characteristics of the treatment network. This may include commentary on the abundance of trials and randomized patients for the different interventions and pairwise comparisons in the network, gaps of evidence in the treatment network, and potential biases reflected by the network structure | 8-9, Figure 2A-B, eFigure 1 |
| Study characteristics | 18 | For each study, present characteristics for which data were extracted (e.g., study size, PICOS, follow-up period) and provide the citations | 8-9, eTable 5 |
| Risk of bias within studies | 19 | Present data on risk of bias of each study and, if available, any outcome level assessment | 8-10, eFigure 3A-B |
| Results of individual studies | 20 | For all outcomes considered (benefits or harms), present, for each study: 1) simple summary data for each intervention group, and 2) effect estimates and confidence intervals. Modified approaches may be needed to deal with information from larger networks | 8-10, eTable 5 |
| Synthesis of results | 21 | Present results of each meta-analysis done, including confidence/credible intervals. In larger networks, authors may focus on comparisons versus a particular comparator (e.g., placebo or standard care), with full findings presented in an appendix. League tables and forest plots may be considered to summarize pairwise comparisons. If additional summary measures were explored (such as treatment rankings), these should also be presented. | 8-10, Figure 3A-B, eFigure 2 |
| Exploration for inconsistency | S5 | Describe results from investigations of inconsistency. This may include such information as measures of model fit to compare consistency and inconsistency models, P values from statistical tests, or summary of inconsistency estimates from different parts of the treatment network. | 9-11, eTable 7-8 |
| Risk of bias across studies | 22 | Present results of any assessment of risk of bias across studies for the evidence base being studied | 9-11, eTable 7-8 |
| Additional analysis | 23 | Give results of additional analyses, if done (e.g., sensitivity or subgroup analyses, meta-regression analyses, alternative network geometries studied, alternative choice of prior distributions for Bayesian analyses, and so forth). | 9-11 |
| **DISCUSSION** | | | |
| Summary of evidence | 24 | Summarize the main findings, including the strength of evidence for each main outcome; consider their relevance to key groups (e.g., health care providers, researchers, and policymakers). | 12-14 |
| Limitations | 25 | Discuss limitations at study and outcome level (e.g., risk of bias), and at review level (e.g., incomplete retrieval of identified research, reporting bias). Comment on the validity of the assumptions, such as transitivity and consistency. Comment on any concerns regarding network geometry (e.g., avoidance of certain comparisons). | 14-15 |
| Conclusions | 26 | Provide a general interpretation of the results in the context of other evidence, and implications for future research. | 16 |
| **FUNDING** | | | |
| Funding | 27 | Describe sources of funding for the systematic review and other support (e.g., supply of data); role of funders for the systematic review. This should also include information regarding whether funding has been received from manufacturers of treatments in the network and/or whether some of the authors are content experts with professional conflicts of interest that could affect use of treatments in the network. | 17 |

*From:* Hutton B, Salanti G, Caldwell DM, et al. The PRISMA extension statement for reporting of systematic reviews incorporating network meta-analyses of health care interventions: checklist and explanations. *Ann Intern Med.* 2015;162(11):777-784.

**eTable 3: Keyword applied in each database**

| Database | Keyword | Filter | Date | Result |
| --- | --- | --- | --- | --- |
| PubMed | (beta blocker OR adrenergic beta-antagonist OR beta-adrenergic blockade OR β-blocker OR acebutolol OR adaprolol OR adimolol OR afurolol OR alprenolol OR amosulalol OR ancarolol OR arnolol OR arotinolol OR atenolol OR befunolol OR betaxolol OR bevantolol OR bisoprolol OR bometolol OR bopindolol OR bornaprolol OR brefonalol OR bromoacetylalprenololmenthane OR bucindolol OR bucumolol OR bufetolol OR bufuralol OR bunitrolol OR bunolol OR bupranolol OR butoﬁlolol OR butoxamine OR carazolol OR carpindolol OR carteolol OR carvedilol OR celiprolol OR cetamolol OR cicloprolol OR cinamolol OR cloranolol OR dexpropranolol OR diacetolol OR draquinolol OR ecastolol OR epanolol OR ericolol OR esatenolol OR esmolol OR exaprolol OR falintolol OR ﬂestolol OR ﬂusoxolol OR idropranolol OR indenolol OR indopanolol OR iprocrolol OR isoxaprolol OR labetalol OR landiolol OR levobetaxolol OR levobunolol OR levomoprolol OR medroxalol OR mepindolol OR metipranolol OR metoprolol OR moprolol OR nadolol OR nadoxolol OR nebivolol OR nifenalol OR nipradilol OR oxprenolol OR pacrinolol OR pafenolol OR pamatolol OR pargolol OR penbutolol OR penirolol OR pindolol OR pirepolol OR practolol OR primidolol OR prizidilol OR procinolol OR propranolol OR ridazolol OR ronactolol OR soquinolol OR sotalol OR spirendolol OR talinolol OR tazolol OR tertatolol OR tienoxolol OR tilisolol OR timolol OR tiprenolol OR tolamolol OR tribendilol OR trigevolol OR xibenolol OR xipranolol) AND (asthma OR dyspnea OR bronchoconstriction OR bronchial constriction OR bronchial hyperreactivity OR respiratory sound OR wheeze OR wheezing) | Randomized controlled trials | 2020/06/05 | 175 |
| ClinicalKey | (beta blocker OR adrenergic beta-antagonist OR beta-adrenergic blockade OR β-blocker) AND (asthma OR bronchoconstriction OR wheeze OR wheezing) | n/a | 2020/06/05 | 18 |
| Cochrane CENTRAL | (beta blocker OR adrenergic beta-antagonist OR beta-adrenergic blockade OR β-blocker OR acebutolol OR adaprolol OR adimolol OR afurolol OR alprenolol OR amosulalol OR ancarolol OR arnolol OR arotinolol OR atenolol OR befunolol OR betaxolol OR bevantolol OR bisoprolol OR bometolol OR bopindolol OR bornaprolol OR brefonalol OR bromoacetylalprenololmenthane OR bucindolol OR bucumolol OR bufetolol OR bufuralol OR bunitrolol OR bunolol OR bupranolol OR butoﬁlolol OR butoxamine OR carazolol OR carpindolol OR carteolol OR carvedilol OR celiprolol OR cetamolol OR cicloprolol OR cinamolol OR cloranolol OR dexpropranolol OR diacetolol OR draquinolol OR ecastolol OR epanolol OR ericolol OR esatenolol OR esmolol OR exaprolol OR falintolol OR ﬂestolol OR ﬂusoxolol OR idropranolol OR indenolol OR indopanolol OR iprocrolol OR isoxaprolol OR labetalol OR landiolol OR levobetaxolol OR levobunolol OR levomoprolol OR medroxalol OR mepindolol OR metipranolol OR metoprolol OR moprolol OR nadolol OR nadoxolol OR nebivolol OR nifenalol OR nipradilol OR oxprenolol OR pacrinolol OR pafenolol OR pamatolol OR pargolol OR penbutolol OR penirolol OR pindolol OR pirepolol OR practolol OR primidolol OR prizidilol OR procinolol OR propranolol OR ridazolol OR ronactolol OR soquinolol OR sotalol OR spirendolol OR talinolol OR tazolol OR tertatolol OR tienoxolol OR tilisolol OR timolol OR tiprenolol OR tolamolol OR tribendilol OR trigevolol OR xibenolol OR xipranolol) AND (asthma OR dyspnea OR bronchoconstriction OR bronchial constriction OR bronchial hyperreactivity OR respiratory sound OR wheeze OR wheezing) | n/a | 2020/06/05 | 883 |
| Embase | (beta blocker OR adrenergic beta-antagonist OR beta-adrenergic blockade OR β-blocker OR acebutolol OR adaprolol OR adimolol OR afurolol OR alprenolol OR amosulalol OR ancarolol OR arnolol OR arotinolol OR atenolol OR befunolol OR betaxolol OR bevantolol OR bisoprolol OR bometolol OR bopindolol OR bornaprolol OR brefonalol OR bromoacetylalprenololmenthane OR bucindolol OR bucumolol OR bufetolol OR bufuralol OR bunitrolol OR bunolol OR bupranolol OR butoﬁlolol OR butoxamine OR carazolol OR carpindolol OR carteolol OR carvedilol OR celiprolol OR cetamolol OR cicloprolol OR cinamolol OR cloranolol OR dexpropranolol OR diacetolol OR draquinolol OR ecastolol OR epanolol OR ericolol OR esatenolol OR esmolol OR exaprolol OR falintolol OR ﬂestolol OR ﬂusoxolol OR idropranolol OR indenolol OR indopanolol OR iprocrolol OR isoxaprolol OR labetalol OR landiolol OR levobetaxolol OR levobunolol OR levomoprolol OR medroxalol OR mepindolol OR metipranolol OR metoprolol OR moprolol OR nadolol OR nadoxolol OR nebivolol OR nifenalol OR nipradilol OR oxprenolol OR pacrinolol OR pafenolol OR pamatolol OR pargolol OR penbutolol OR penirolol OR pindolol OR pirepolol OR practolol OR primidolol OR prizidilol OR procinolol OR propranolol OR ridazolol OR ronactolol OR soquinolol OR sotalol OR spirendolol OR talinolol OR tazolol OR tertatolol OR tienoxolol OR tilisolol OR timolol OR tiprenolol OR tolamolol OR tribendilol OR trigevolol OR xibenolol OR xipranolol) AND (asthma OR dyspnea OR bronchoconstriction OR bronchial constriction OR bronchial hyperreactivity OR respiratory sound OR wheeze OR wheezing) | n/a | 2020/06/05 | 582 |
| ProQuest | (beta blocker OR adrenergic beta-antagonist OR beta-adrenergic blockade OR β-blocker) AND (asthma OR bronchoconstriction OR wheeze OR wheezing) AND (randomized control trial) | n/a | 2020/06/05 | 342 |
| ScienceDirect | (beta blocker OR adrenergic beta-antagonist OR beta-adrenergic blockade OR β-blocker) AND (asthma OR bronchoconstriction OR wheeze OR wheezing) AND (randomized control trial) | research article | 2020/06/05 | 4358 |
| Web of Science | (beta blocker OR adrenergic beta-antagonist OR beta-adrenergic blockade OR β-blocker OR acebutolol OR adaprolol OR adimolol OR afurolol OR alprenolol OR amosulalol OR ancarolol OR arnolol OR arotinolol OR atenolol OR befunolol OR betaxolol OR bevantolol OR bisoprolol OR bometolol OR bopindolol OR bornaprolol OR brefonalol OR bromoacetylalprenololmenthane OR bucindolol OR bucumolol OR bufetolol OR bufuralol OR bunitrolol OR bunolol OR bupranolol OR butoﬁlolol OR butoxamine OR carazolol OR carpindolol OR carteolol OR carvedilol OR celiprolol OR cetamolol OR cicloprolol OR cinamolol OR cloranolol OR dexpropranolol OR diacetolol OR draquinolol OR ecastolol OR epanolol OR ericolol OR esatenolol OR esmolol OR exaprolol OR falintolol OR ﬂestolol OR ﬂusoxolol OR idropranolol OR indenolol OR indopanolol OR iprocrolol OR isoxaprolol OR labetalol OR landiolol OR levobetaxolol OR levobunolol OR levomoprolol OR medroxalol OR mepindolol OR metipranolol OR metoprolol OR moprolol OR nadolol OR nadoxolol OR nebivolol OR nifenalol OR nipradilol OR oxprenolol OR pacrinolol OR pafenolol OR pamatolol OR pargolol OR penbutolol OR penirolol OR pindolol OR pirepolol OR practolol OR primidolol OR prizidilol OR procinolol OR propranolol OR ridazolol OR ronactolol OR soquinolol OR sotalol OR spirendolol OR talinolol OR tazolol OR tertatolol OR tienoxolol OR tilisolol OR timolol OR tiprenolol OR tolamolol OR tribendilol OR trigevolol OR xibenolol OR xipranolol) AND (asthma OR dyspnea OR bronchoconstriction OR bronchial constriction OR bronchial hyperreactivity OR respiratory sound OR wheeze OR wheezing) | n/a | 2020/06/05 | 1217 |

Abbreviation: n/a: not apply

**eTable 4: Excluded studies and reason**

| Reason | Numbers | References |
| --- | --- | --- |
| All the groups had 0 patients with asthma exacerbation | 13 | [1-13] |
| Also concomitant used other beta-blocker which was the initially randomized beta-blocker | 1 | [14] |
| Could not form the connection | 1 | [15] |
| Excluded subjects without asthmatic exacerbation by propranolol challenge | 1 | [16] |
| Lack of sufficient data | 6 | [17-22] |
| Meta-analysis | 2 | [23, 24] |
| Not compared to beta-blocker prescription or placebo-control | 32 | [25-56] |
| Nor randomized according to beta-blocker allocation | 1 | [57] |
| Not randomized controlled trial | 10 | [58-67] |
| Not related to investigation of beta-blocker | 47 | [68-114] |
| Not reported adverse event of asthma attack | 42 | [115-156] |
| Rating scale validation but not report of study result | 1 | [157] |
| Review article | 2 | [158, 159] |

**References:**

1. Brooks AM, Burden JG, Gillies WE: The significance of reactions to betaxolol reported by patients. Aust N Z J Ophthalmol. 1989; 17(4):353-5.

2. Dorow P, Bethge H, Tonnesmann U: Effects of single oral doses of bisoprolol and atenolol on airway function in nonasthmatic chronic obstructive lung disease and angina pectoris. Eur J Clin Pharmacol. 1986; 31(2):143-7.

3. Philip-Joet F, Saadjian A, Bruguerolle B, Arnaud A: Comparative study of the respiratory effects of two beta 1-selective blocking agents atenolol and bevantolol in asthmatic patients. Eur J Clin Pharmacol. 1986; 30(1):13-6.

4. Chatterjee SS: The cardioselective and hypotensive effects of bisoprolol in hypertensive asthmatics. J Cardiovasc Pharmacol. 1986; 8 Suppl 11:S74-7.

5. Matthys H, Doshan HD, Ruhle KH, Applin WJ, Braig H, Pohl M: Bronchosparing properties of celiprolol, a new beta 1, alpha 2-blocker, in propranolol-sensitive asthmatic patients. J Cardiovasc Pharmacol. 1986; 8 Suppl 4:S40-2.

6. Matthys H, Doshan HD, Ruhle KH, Braig H, Pohl M, Applin WJ, Caruso FS, Neiss ES: The bronchosparing effect of celiprolol, a new beta 1- alpha 2-receptor antagonist on pulmonary function of propranolol-sensitive asthmatics. Journal of clinical pharmacology. 1985; 25(5):354-9.

7. Patakas D, Argiropoulou V, Louridas G, Tsara V: Beta-blockers in bronchial asthma: effect of propranolol and pindolol on large and small airways. Thorax. 1983; 38(2):108-12.

8. Ranchod A, Keeton GR, Benatar SR: The effect of beta-blockers on ventilatory function in chronic bronchitis. S Afr Med J. 1982; 61(12):423-4.

9. Formgren H: The effect of metoprolol and practolol on lung function and blood pressure in hypertensive asthmatics. Br J Clin Pharmacol. 1976; 3(6):1007-14.

10. Connolly CK, Batten JC: Comparison of the effect of alprenolol and propranolol on specific airway conductance in asthmatic subjects. British medical journal. 1970; 2(5708):515-6.

11. Yamakage M, Iwasaki S, Jeong SW, Satoh J, Namiki A: Beta-1 selective adrenergic antagonist landiolol and esmolol can be safely used in patients with airway hyperreactivity. Heart Lung. 2009; 38(1):48-55.

12. Maclean D, Mitchell ET, Lewis R, Irvine N, McLay JS, McEwen J, Coulson RR, Slater ND, Fitzsimons TJ, McDevitt DG: Comparison of once daily atenolol, nitrendipine and their combination in mild to moderate essential hypertension. Br J Clin Pharmacol. 1990; 29(4):455-63.

13. von Wichert P, Boes J: [Beta-receptor blocking drugs and the bronchial system. A comparative study of pindolol and metoprolol (author's transl)]. Med Klin. 1979; 74(42):1544-7.

14. Herlitz J, Bengtson A, Wiklund I, Hjalmarson A: Morbidity and quality of life 5 years after early intervention with metoprolol in suspected acute myocardial infarction. Cardiology. 1988; 75(5):357-64.

15. Seamone C, LeBlanc R, Saheb N, Novack G: Efficacy of twice-daily levobunolol in the treatment of elevated intraocular pressure. Can J Ophthalmol. 1988; 23(4):168-70.

16. Myers JD, Higham MA, Shakur BH, Wickremasinghe M, Ind PW: Attenuation of propranolol-induced bronchoconstriction by frusemide. Thorax. 1997; 52(10):861-5.

17. Lindholt JS, Henneberg EW, Juul S, Fasting H: Impaired results of a randomised double blinded clinical trial of propranolol versus placebo on the expansion rate of small abdominal aortic aneurysms. Int Angiol. 1999; 18(1):52-7.

18. Hellstrom PE, Salonen T: [Therapeutic differences between atenolol and pindolol in asthmatic patients]. Duodecim. 1979; 95(6):287-91.

19. Von Graffenried B: [Competitive effects of pindolol and salbutamol on airway resistance in asthmatics]. Nouv Presse Med. 1978; 7(31):2717-20.

20. Vilsvik JS, Schaanning J: Effect of atenolol on cardiac and ventilatory function in patients with chronic asthma. Postgraduate medical journal. 1977; 53 Suppl 3:142.

21. Nestel PJ: Evaluation of propranolol ("Inderal") in the treatment of angina pectoris. Med J Aust. 1966; 2(27):1274-6.

22. Repsher LH, Vincent ME, Medakovic M: The effect of single doses of labetalol, metoprolol and placebo on ventilatory function in patients with bronchial asthma. Journal of Hypertension. 1986; 4(suppl 5):S510-S2.

23. Morales DR, Jackson C, Lipworth BJ, Donnan PT, Guthrie B: Adverse respiratory effect of acute beta-blocker exposure in asthma: a systematic review and meta-analysis of randomized controlled trials. Chest. 2014; 145(4):779-86.

24. Salpeter S, Ormiston T, Salpeter E: Cardioselective beta-blockers for reversible airway disease. The Cochrane database of systematic reviews. 2002(4):CD002992.

25. Anderson WJ, Short PM, Williamson PA, Manoharan A, Lipworth BJ: The inverse agonist propranolol confers no corticosteroid-sparing activity in mild-to-moderate persistent asthma. Clin Sci (Lond). 2014; 127(11):635-43.

26. Buhl R, Dunn LJ, Disdier C, Lassen C, Amos C, Henley M, Kramer B, investigators Is: Blinded 12-week comparison of once-daily indacaterol and tiotropium in COPD. Eur Respir J. 2011; 38(4):797-803.

27. Ageev FT, Makarova GV, Patrusheva IF, Orlova Ia A: [The efficacy and safety of the combination of beta-blocker bisoprolol and if inhibitor I(f) ivabradine in patients with stable angina and chronic obstructive pulmonary disease]. Kardiologiia. 2010; 50(10):22-6.

28. Dal Negro RW, Micheletto C, Tognella S, Trevisan F, Guerriero M: The therapeutic effects of inhaled long-acting beta2-adrenergics (LABA) and corticosteroids (ICS) are not affected by their inhalation sequence in moderate/persistent asthma. Eur Ann Allergy Clin Immunol. 2006; 38(5):153-7.

29. Abraham WT, Cheng ML, Smoluk G, Vasodilation in the Management of Acute Congestive Heart Failure Study G: Clinical and hemodynamic effects of nesiritide (B-type natriuretic peptide) in patients with decompensated heart failure receiving beta blockers. Congest Heart Fail. 2005; 11(2):59-64.

30. Alan S, Ulgen MS, Ozdemir K, Keles T, Toprak N: Reliability and efficacy of metoprolol and diltiazem in patients having mild to moderate mitral stenosis with sinus rhythm. Angiology. 2002; 53(5):575-81.

31. Bulpitt CJ, Connor M, Schulte M, Fletcher AE: Bisoprolol and nifedipine retard in elderly hypertensive patients: effect on quality of life. J Hum Hypertens. 2000; 14(3):205-12.

32. Chen SA, Hsieh MH, Tai CT, Tsai CF, Prakash VS, Yu WC, Hsu TL, Ding YA, Chang MS: Initiation of atrial fibrillation by ectopic beats originating from the pulmonary veins: electrophysiological characteristics, pharmacological responses, and effects of radiofrequency ablation. Circulation. 1999; 100(18):1879-86.

33. Clauzel AM, Jean T, Etienne R, Visier S, Michel F: Effect of long-term treatment with celiprolol on pulmonary function in a group of mild hypertensive asthmatics. J Int Med Res. 1988; 16 Suppl 1:27A-33A.

34. Cheah JS: Comparison between reserpine and propranolol as adjuncts in the treatment of hyperthyroidism. Curr Med Res Opin. 1973; 1(7):407-11.

35. Konstas AG, Papapanos P, Tersis I, Houliara D, Stewart WC: Twenty-four-hour diurnal curve comparison of commercially available latanoprost 0.005% versus the timolol and dorzolamide fixed combination. Ophthalmology. 2003; 110(7):1357-60.

36. Jimenez-Jimenez FJ, Garcia-Ruiz PJ, Cabrera-Valdivia F: Nicardipine versus propranolol in essential tumor. Acta Neurol (Napoli). 1994; 16(4):184-8.

37. Diggory P, Heyworth P, Chau G, McKenzie S, Sharma A: Unsuspected bronchospasm in association with topical timolol--a common problem in elderly people: can we easily identify those affected and do cardioselective agents lead to improvement? Age and ageing. 1994; 23(1):17-21.

38. Diggory P, Heyworth P, Chau G, McKenzie S, Sharma A, Luke I: Improved lung function tests on changing from topical timolol: non-selective beta-blockade impairs lung function tests in elderly patients. Eye (Lond). 1993; 7 ( Pt 5):661-3.

39. James MA, Channer KS, Papouchado M, Rees JR: Improved control of atrial fibrillation with combined pindolol and digoxin therapy. Eur Heart J. 1989; 10(1):83-90.

40. Nifedipine and atenolol singly and combined for treatment of essential hypertension: comparative multicentre study in general practice in the United Kingdom. Nifedipine-Atenolol Study Review Committee. British medical journal (Clinical research ed). 1988; 296(6620):468-72.

41. Dorow P: Bronchoneutral effects in hypertensive asthmatics--celiprolol versus chlorthalidone. J Int Med Res. 1988; 16 Suppl 1:23A-6A.

42. Falliers CJ, Vrchota J, Blasucci DJ, Maloy JW, Medakovic M: The effects of treatments with labetalol and hydrochlorothiazide on ventilatory function of asthmatic hypertensive patients with demonstrated bronchosensitivity to propranolol. J Clin Hypertens. 1985; 1(1):70-9.

43. Koeter GH, Meurs H, de Monchy JG, de Vries K: Protective effect of disodium cromoglycate on propranolol challenge. Allergy. 1982; 37(8):587-90.

44. Eggertsen R, Hansson L: Effects of treatment with nifedipine and metoprolol in essential hypertension. Eur J Clin Pharmacol. 1982; 21(5):389-90.

45. Saggu DK, Narain VS, Dwivedi SK, Sethi R, Chandra S, Puri A, Saran RK: Effect of Ivabradine on Heart Rate and Duration of Exercise in Patients With Mild-to-Moderate Mitral Stenosis: A Randomized Comparison With Metoprolol. J Cardiovasc Pharmacol. 2015; 65(6):552-4.

46. Shah HA, Azam Z, Rauf J, Abid S, Hamid S, Jafri W, Khalid A, Ismail FW, Parkash O, Subhan A *et al*: Carvedilol vs. esophageal variceal band ligation in the primary prophylaxis of variceal hemorrhage: a multicentre randomized controlled trial. J Hepatol. 2014; 60(4):757-64.

47. Thaivalappil S, Bauman N, Saieg A, Movius E, Brown KJ, Preciado D: Propranolol-mediated attenuation of MMP-9 excretion in infants with hemangiomas. JAMA Otolaryngol Head Neck Surg. 2013; 139(10):1026-31.

48. Nagaoka E, Arai H, Tamura K, Makita S, Miyagi N: Prevention of atrial fibrillation with ultra-low dose landiolol after off-pump coronary artery bypass grafting. Ann Thorac Cardiovasc Surg. 2014; 20(2):129-34.

49. Taylor DR, Town GI, Herbison GP, Boothman-Burrell D, Flannery EM, Hancox B, Harre E, Laubscher K, Linscott V, Ramsay CM *et al*: Asthma control during long-term treatment with regular inhaled salbutamol and salmeterol. Thorax. 1998; 53(9):744-52.

50. Mackay TW, Hulks G, Douglas NJ: Non-adrenergic, non-cholinergic function in the human airway. Respir Med. 1998; 92(3):461-6.

51. Quiret JC, Gillet JC, Rey JL, Lombaert M, Bernasconi P: [Propranolol-nifedipine combination in the treatment of exercise-induced asthma]. Arch Mal Coeur Vaiss. 1983; 76(10):1171-7.

52. Adverse reactions to bendrofluazide and propranolol for the treatment of mild hypertension. Report of Medical Research Council Working Party on Mild to Moderate Hypertension. Lancet. 1981; 2(8246):539-43.

53. Kristensen BO, Brons M, Christensen CK, Geday E, Jacobsen FK, Jensen SN, Linde NC: Antihypertensive effect of atenolol (100 mg once a day) and methyldopa (250 mg thrice a day). A double-blind cross-over multicentre study. Acta medica Scandinavica. 1981; 209(4):267-70.

54. Orehek J, Gayrard P, Grimaud C, Charpin J: Effect of beta adrenergic blockage on bronchial sensitivity to inhaled acetylcholine in normal subjects. J Allergy Clin Immunol. 1975; 55(3):164-9.

55. Vonwil A, Landolt M, Flammer J, Bachofen H: [Bronchoconstrictive side effects of timolol eye drops in patients with obstructive lung disease]. Schweiz Med Wochenschr. 1981; 111(19):665-9.

56. Vorburger C: [The anti-hypertensive effect of timolol maleate (blocadren) in gradated combination with a diuretic]. Schweiz Med Wochenschr. 1976; 106(43):1474-81.

57. Tagami T, Yambe Y, Tanaka T, Tanaka T, Ogo A, Yoshizumi H, Kaise K, Higashi K, Tanabe M, Shimazu S *et al*: Short-term effects of beta-adrenergic antagonists and methimazole in new-onset thyrotoxicosis caused by Graves' disease. Internal medicine. 2012; 51(17):2285-90.

58. Sun Y, Yu J, Hu D, Investigator of C: [Status of beta-blocker use and heart rate control in Chinese patients with stable coronary artery disease]. Zhonghua Xin Xue Guan Bing Za Zhi. 2016; 44(1):19-26.

59. Anderson WJ, Short PM, Manoharan A, Lipworth JL, Lipworth BJ: Influence of beta2-adrenoceptor 16 genotype on propranolol-induced bronchoconstriction in patients with persistent asthma. Ann Allergy Asthma Immunol. 2014; 112(5):475-6.

60. Short PM, Williamson PA, Lipworth BJ: Sensitivity of impulse oscillometry and spirometry in beta-blocker induced bronchoconstriction and beta-agonist bronchodilatation in asthma. Ann Allergy Asthma Immunol. 2012; 109(6):412-5.

61. Damman K, Voors AA, Hillege HL, Navis G, Lechat P, van Veldhuisen DJ, Dargie HJ, Investigators C-, Committees: Congestion in chronic systolic heart failure is related to renal dysfunction and increased mortality. Eur J Heart Fail. 2010; 12(9):974-82.

62. Russell SD, Saval MA, Robbins JL, Ellestad MH, Gottlieb SS, Handberg EM, Zhou Y, Chandler B, Investigators H-A: New York Heart Association functional class predicts exercise parameters in the current era. Am Heart J. 2009; 158(4 Suppl):S24-30.

63. Hauber AB, Mohamed AF, Johnson FR, Meddis D, Wagner S, O'Dowd L: Quantifying asthma patient preferences for onset of effect of combination inhaled corticosteroids and long-acting beta2-agonist maintenance medications. Allergy Asthma Proc. 2009; 30(2):139-47.

64. Ekman I, Cleland JG, Swedberg K, Charlesworth A, Metra M, Poole-Wilson PA: Symptoms in patients with heart failure are prognostic predictors: insights from COMET. Journal of cardiac failure. 2005; 11(4):288-92.

65. Witte KK, Thackray SD, Nikitin NP, Cleland JG, Clark AL: The effects of alpha and beta blockade on ventilatory responses to exercise in chronic heart failure. Heart. 2003; 89(10):1169-73.

66. Mafrici A, Mauri F, Maggioni AP, Franzosi MG, Santoro L, De Vita C: [Atenolol i.v. in the acute phase of AMI: the indications, contraindications and interactions with thrombolytic drugs in the GISSI-2 study. The GISSI-2 Researchers. Gruppo Italiano per lo Studio della Streptochinasi nell'Infarto Miocardico]. G Ital Cardiol. 1995; 25(3):353-64.

67. Ruffin RE, Crockett AJ, Alpers JH: The effect of bucindolol on the airway function of asthmatics. Eur J Clin Pharmacol. 1986; 30(5):559-65.

68. Busse WW, O'Byrne PM, Bleecker ER, Lotvall J, Woodcock A, Andersen L, Hicks W, Crawford J, Jacques L, Apoux L *et al*: Safety and tolerability of the novel inhaled corticosteroid fluticasone furoate in combination with the beta2 agonist vilanterol administered once daily for 52 weeks in patients >=12 years old with asthma: a randomised trial. Thorax. 2013; 68(6):513-20.

69. Ahmadiafshar A, Maarefvand M, Taymourzade B, Mazloomzadeh S, Torabi Z: Efficacy of sublingual swallow immunotherapy in children with rye grass pollen allergic rhinitis: a double-blind placebo-controlled study. Iran J Allergy Asthma Immunol. 2012; 11(2):175-81.

70. Avery AJ, Rodgers S, Cantrill JA, Armstrong S, Cresswell K, Eden M, Elliott RA, Howard R, Kendrick D, Morris CJ *et al*: A pharmacist-led information technology intervention for medication errors (PINCER): a multicentre, cluster randomised, controlled trial and cost-effectiveness analysis. Lancet. 2012; 379(9823):1310-9.

71. Bergh CH, Andersson B, Dahlstrom U, Forfang K, Kivikko M, Sarapohja T, Ullman B, Wikstrom G: Intravenous levosimendan vs. dobutamine in acute decompensated heart failure patients on beta-blockers. Eur J Heart Fail. 2010; 12(4):404-10.

72. Blanchet M, Ducharme A, Racine N, Rouleau JL, Tardif JC, Juneau M, Marquis J, Larivee L, Nigam A, Fortier A *et al*: Effects of cold exposure on submaximal exercise performance and adrenergic activation in patients with congestive heart failure and the effects of beta-adrenergic blockade (carvedilol or metoprolol). Am J Cardiol. 2003; 92(5):548-53.

73. de Jong JW, Teengs JP, Postma DS, van der Mark TW, Koeter GH, de Monchy JG: Nedocromil sodium versus albuterol in the management of allergic asthma. Am J Respir Crit Care Med. 1994; 149(1):91-7.

74. Carpentiere G, Castello F, Marino S: Increased responsiveness to histamine after propranolol in subjects with asthma nonresponsive to the bronchoconstrictive effect of propranolol. J Allergy Clin Immunol. 1988; 82(4):595-8.

75. Crimi N, Palermo F, Oliveri R, Palermo B, Vancheri C, Polosa R, Mistretta A: Effect of vasoactive intestinal peptide (VIP) on propranolol-induced bronchoconstriction. J Allergy Clin Immunol. 1988; 82(4):617-21.

76. Buhler FR, Vesanen K, Watters JT, Bolli P: Impact of smoking on heart attacks, strokes, blood pressure control, drug dose, and quality of life aspects in the International Prospective Primary Prevention Study in Hypertension. Am Heart J. 1988; 115(1 Pt 2):282-8.

77. Busse WW, Sharpe G, Smith A, Arbabian M, Borgen L, Ruoho A: The effect of procaterol treatment on beta-adrenergic bronchodilation and polymorphonuclear leukocyte responsiveness. Am Rev Respir Dis. 1985; 132(6):1194-8.

78. Djaladat H, Tajik P, Fard SA, Alehashemi S: The effect of aminophylline on renal colic: a randomized double blind controlled trial. South Med J. 2007; 100(11):1081-4.

79. Faradzheva NA: [Efficiency of a combination of haloaerosols and helium-neon laser in the multimodality treatment of patients with bronchial asthma]. Probl Tuberk Bolezn Legk. 2007(8):50-3.

80. Diaz O, Begin P, Andresen M, Prieto ME, Castillo C, Jorquera J, Lisboa C: Physiological and clinical effects of diurnal noninvasive ventilation in hypercapnic COPD. Eur Respir J. 2005; 26(6):1016-23.

81. Fireman P, Prenner BM, Vincken W, Demedts M, Mol SJ, Cohen RM: Long-term safety and efficacy of a chlorofluorocarbon-free beclomethasone dipropionate extrafine aerosol. Ann Allergy Asthma Immunol. 2001; 86(5):557-65.

82. Fujimura M, Abo M, Kamio Y, Myou S, Ishiura Y, Hashimoto T, Matsuda T: Effect of leukotriene and thromboxane antagonist on propranolol-induced bronchoconstriction. Am J Respir Crit Care Med. 1999; 160(6):2100-3.

83. Grant S, Aitchison T, Henderson E, Christie J, Zare S, McMurray J, Dargie H: A comparison of the reproducibility and the sensitivity to change of visual analogue scales, Borg scales, and Likert scales in normal subjects during submaximal exercise. Chest. 1999; 116(5):1208-17.

84. Drotar DE, Davis EE, Cockcroft DW: Tolerance to the bronchoprotective effect of salmeterol 12 hours after starting twice daily treatment. Ann Allergy Asthma Immunol. 1998; 80(1):31-4.

85. Ganassini A, Rossi A: Short-term regular beta 2-adrenergic agonists treatment is safe in mild asthmatics taking low doses of inhaled steroids. J Asthma. 1997; 34(1):61-6.

86. Foresi A, Chetta A, Pelucchi A, Cavigioli G, Mastropasqua B, Olivieri D: Effect of inhaled disodium cromoglycate and nedocromil sodium on propranolol-induced bronchoconstriction. Ann Allergy. 1993; 70(2):159-63.

87. Glover DR, Wathen CG, Murray RG, Petch MC, Muir AL, Littler WA: Are the clinical benefits of oral prenalterol in ischaemic heart failure due to beta blockade? A six month randomised double blind comparison with placebo. Br Heart J. 1985; 53(2):208-15.

88. Ibero M, Castillo MJ: Significant improvement of specific bronchial hyperreactivity in asthmatic children after 4 months of treatment with a modified extract of dermatophagoides pteronyssinus. J Investig Allergol Clin Immunol. 2006; 16(3):194-202.

89. He WQ, Zheng JP, Ran PX, Liu HZ, Lin H, Cai DM, Xu JM, Zhong NS: [Effects of H1 blocker and inhaled corticosteroids on asthmatic patients with allergic rhinitis]. Zhonghua Jie He He Hu Xi Za Zhi. 2003; 26(3):157-60.

90. Hussaini SH, Henderson T, Morrell AJ, Losowsky MS: Dark adaptation in early primary biliary cirrhosis. Eye (Lond). 1998; 12 ( Pt 3a):419-26.

91. Grove A, McFarlane LC, Lipworth BJ: Expression of the beta 2 adrenoceptor partial agonist/antagonist activity of salbutamol in states of low and high adrenergic tone. Thorax. 1995; 50(2):134-8.

92. Green CP, Price JF: Prevention of exercise induced asthma by inhaled salmeterol xinafoate. Arch Dis Child. 1992; 67(8):1014-7.

93. Hoppe M, Harman E, Hendeles L: The effect of inhaled gallopamil, a potent calcium channel blocker, on the late-phase response in subjects with allergic asthma. J Allergy Clin Immunol. 1992; 89(3):688-95.

94. Latimer KM, Ruffin RE: The effect of inhaled fenoterol and ipratropium bromide on propranolol induced bronchoconstriction in the asthmatic airways. Clinical and experimental pharmacology & physiology. 1990; 17(9):627-35.

95. Ind PW, Dixon CM, Fuller RW, Barnes PJ: Anticholinergic blockade of beta-blocker-induced bronchoconstriction. Am Rev Respir Dis. 1989; 139(6):1390-4.

96. Kraan J, Koeter GH, vd Mark TW, Sluiter HJ, de Vries K: Changes in bronchial hyperreactivity induced by 4 weeks of treatment with antiasthmatic drugs in patients with allergic asthma: a comparison between budesonide and terbutaline. J Allergy Clin Immunol. 1985; 76(4):628-36.

97. Koeter GH, Meurs H, Jonkman JH, Greving J, Leferink J, Sluiter HJ, de Zeeuw RA, de Vries K: Protective effect of oral oxyphenonium bromide, terbutaline and theophylline against the bronchial obstructive effects of inhaled histamine, acetylcholine and propranolol. Eur J Clin Pharmacol. 1984; 26(4):435-41.

98. Morell F, Ojanguren I, Cordovilla R, Urrutia I, Aguero R, Guerra J, Genover T, Ramon MA, Group AS: Two short interventions to reduce health care requirements in asthma patients. A multicentre controlled study (ASTHMACAP II). Med Clin (Barc). 2014; 142(8):348-54.

99. Makela MJ, Malmberg LP, Csonka P, Klemola T, Kajosaari M, Pelkonen AS: Salmeterol and fluticasone in young children with multiple-trigger wheeze. Ann Allergy Asthma Immunol. 2012; 109(1):65-70.

100. Rubin AS, Souza-Machado A, Andradre-Lima M, Ferreira F, Honda A, Matozo TM, Investigators QS: Effect of omalizumab as add-on therapy on asthma-related quality of life in severe allergic asthma: a Brazilian study (QUALITX). J Asthma. 2012; 49(3):288-93.

101. Short PM, Williamson PA, Lipworth BJ: Effects of hydrocortisone on acute beta-adrenoceptor blocker and histamine induced bronchoconstriction. Br J Clin Pharmacol. 2012; 73(5):717-26.

102. Miric M, Plavec D: Risk of acute bronchospasm and bronchial hyperreactivity from inhaled acid aerosol in healthy subjects: randomized, double-blind controlled trial. Croatian medical journal. 2004; 45(6):709-14.

103. Paggiaro PL, Giannini D, Di Franco A, Testi R: Comparison of inhaled salmeterol and individually dose-titrated slow-release theophylline in patients with reversible airway obstruction. European Study Group. Eur Respir J. 1996; 9(8):1689-95.

104. Palmer JB, Stuart AM, Shepherd GL, Viskum K: Inhaled salmeterol in the treatment of patients with moderate to severe reversible obstructive airways disease--a 3-month comparison of the efficacy and safety of twice-daily salmeterol (100 micrograms) with salmeterol (50 micrograms). Respir Med. 1992; 86(5):409-17.

105. Mackay TW, Fitzpatrick MF, Douglas NJ: Non-adrenergic, non-cholinergic nervous system and overnight airway calibre in asthmatic and normal subjects. Lancet. 1991; 338(8778):1289-92.

106. Lofdahl CG, Svedmyr N: Effect of prenalterol in asthmatic patients. Eur J Clin Pharmacol. 1982; 23(4):297-302.

107. Pegelow KO, Strandberg K: Evaluation of the bronchodilating and antiallergic properties of a beta-adrenoceptor stimulant, KWD 2131, in asthmatic patients. Allergy. 1980; 35(6):509-19.

108. Szefler SJ, Murphy K, Harper T, 3rd, Boner A, Laki I, Engel M, El Azzi G, Moroni-Zentgraf P, Finnigan H, Hamelmann E: A phase III randomized controlled trial of tiotropium add-on therapy in children with severe symptomatic asthma. J Allergy Clin Immunol. 2017; 140(5):1277-87.

109. Su N, Lin JT, Yang M, Chen X, He J, He QY, Cao ZL, Chen BY, Xiao Y, Yan XX: [The efficacy and safety of tulobuterol tape in mild and moderate persistent asthma patients]. Zhonghua nei ke za zhi. 2007; 46(1):39-42.

110. Yurdakul AS, Taci N, Eren A, Sipit T: Comparative efficacy of once-daily therapy with inhaled corticosteroid, leukotriene antagonist or sustained-release theophylline in patients with mild persistent asthma. Respir Med. 2003; 97(12):1313-9.

111. Vaquerizo MJ, Casan P, Castillo J, Perpina M, Sanchis J, Sobradillo V, Valencia A, Verea H, Viejo JL, Villasante C *et al*: Effect of montelukast added to inhaled budesonide on control of mild to moderate asthma. Thorax. 2003; 58(3):204-10.

112. Villanueva C, Minana J, Ortiz J, Gallego A, Soriano G, Torras X, Sainz S, Boadas J, Cusso X, Guarner C *et al*: Endoscopic ligation compared with combined treatment with nadolol and isosorbide mononitrate to prevent recurrent variceal bleeding. N Engl J Med. 2001; 345(9):647-55.

113. van der Schans CP, de Jong W, de Vries G, Postma DS, Koeter GH, van der Mark TW: Respiratory muscle activity and pulmonary function during acutely induced airways obstruction. Physiother Res Int. 1997; 2(3):167-77.

114. Svedmyr K, Lofdahl CG, Svedmyr N: Nifedipine--a calcium channel blocker--in asthmatic patients. Interaction with terbutaline. Allergy. 1984; 39(1):17-22.

115. Clark AL, Coats AJS, Krum H, Katus HA, Mohacsi P, Salekin D, Schultz MK, Packer M, Anker SD: Effect of beta-adrenergic blockade with carvedilol on cachexia in severe chronic heart failure: results from the COPERNICUS trial. Journal of cachexia, sarcopenia and muscle. 2017; 8(4):549-56.

116. Chang CL, Mills GD, McLachlan JD, Karalus NC, Hancox RJ: Cardio-selective and non-selective beta-blockers in chronic obstructive pulmonary disease: effects on bronchodilator response and exercise. Intern Med J. 2010; 40(3):193-200.

117. Beloka S, Gujic M, Deboeck G, Niset G, Ciarka A, Argacha JF, Adamopoulos D, Van de Borne P, Naeije R: Beta-adrenergic blockade and metabo-chemoreflex contributions to exercise capacity. Med Sci Sports Exerc. 2008; 40(11):1932-8.

118. Gandolfi SA, Chetta A, Cimino L, Mora P, Sangermani C, Tardini MG: Bronchial reactivity in healthy individuals undergoing long-term topical treatment with beta-blockers. Arch Ophthalmol. 2005; 123(1):35-8.

119. Bauer KG, Brunner-Ferber F, Distlerath LM, Lippa EA, Binkowitz B, Till P, Kaik GA: Assessment of bronchial effects following topical administration of butylamino-phenoxy-propanol-acetate, an oculoselective beta-adrenoceptor blocker in asthmatic subjects. Br J Clin Pharmacol. 1992; 34(2):122-9.

120. Fogari R, Zoppi A, Tettamanti F, Poletti L, Rizzardi G, Fiocchi G: Comparative effects of celiprolol, propranolol, oxprenolol, and atenolol on respiratory function in hypertensive patients with chronic obstructive lung disease. Cardiovasc Drugs Ther. 1990; 4(4):1145-9.

121. Bohm E, Fabel H: [Changes in lung function following administration of eyedrops containing timolol, metipranolol, pindolol and pilocarpine in healthy probands and patients with mild bronchial asthma]. Klinische Wochenschrift. 1987; 65(19):920-4.

122. Bassan MM, Michaeli J, Shalev O: Failure of propranolol to improve exercise tolerance in patients with mitral stenosis in sinus rhythm. Br Heart J. 1987; 58(3):254-8.

123. Falliers CJ, Vincent ME, Medakovic M: Effect of single doses of labetalol, metoprolol, and placebo on ventilatory function in patients with bronchial asthma: interaction with isoproterenol. J Asthma. 1986; 23(5):251-60.

124. Doshan HD, Rosenthal RR, Brown R, Slutsky A, Applin WJ, Caruso FS: Celiprolol, atenolol and propranolol: a comparison of pulmonary effects in asthmatic patients. J Cardiovasc Pharmacol. 1986; 8 Suppl 4:S105-8.

125. Giddens CL, Barron KW, Clark KF, Warde WD: Beta-adrenergic blockade and voice: a double-blind, placebo-controlled trial. J Voice. 2010; 24(4):477-89.

126. Jondeau G, Neuder Y, Eicher JC, Jourdain P, Fauveau E, Galinier M, Jegou A, Bauer F, Trochu JN, Bouzamondo A *et al*: B-CONVINCED: Beta-blocker CONtinuation Vs. INterruption in patients with Congestive heart failure hospitalizED for a decompensation episode. Eur Heart J. 2009; 30(18):2186-92.

127. Hugues FC, Matte JC, Le Jeunne C, Salem A: Effects of beta-adrenoceptors blocking eye drops in patients with chronic bronchitis. Therapie. 1992; 47(3):211-5.

128. Heublein B, Modersohn D, Franz N, Panzner B: Acute haemodynamic profile of celiprolol in patients with coronary heart disease and hypertension: a double-blind comparison with metoprolol. Eur Heart J. 1991; 12(5):617-23.

129. Hedner J, Ullman A, Lemne C, Svedmyr N: Effects of dilevalol, a beta-adrenoceptor antagonist with intrinsic sympathetic activity in asthmatic patients. Pulm Pharmacol. 1989; 2(3):155-9.

130. Lammers JW, Muller ME, Folgering HT, van Herwaarden CL: A comparative study on the ventilatory and haemodynamic effects of xamoterol and atenolol in asthmatic patients. Br J Clin Pharmacol. 1986; 22(5):595-602.

131. Lammers JW, Folgering HT, van Herwaarden CL: Ventilatory effects of beta 1-receptor-selective blockade with bisoprolol and metoprolol in asthmatic patients. Eur J Clin Pharmacol. 1984; 27(2):141-5.

132. Lofdahl CG, Marlin GE, Svedmyr N: Pafenolol, a highly selective beta 1-adrenoceptor-antagonist, in asthmatic patients: interaction with terbutaline. Clin Pharmacol Ther. 1983; 33(1):1-9.

133. Greefhorst AP, van Herwaarden CL: Ventilatory and haemodynamic effects of terbutaline infusion during beta 1-selective blockade with metoprolol and acebutolol in asthmatic patients. Eur J Clin Pharmacol. 1982; 23(3):203-8.

134. Greefhorst AP, van Herwaarden CL: Comparative study of the ventilatory effects of three beta 1-selective blocking agents in asthmatic patients. Eur J Clin Pharmacol. 1981; 20(6):417-21.

135. Matthys H, Giebelhaus V, von Fallois J: [Nebivolol (nebilet) a beta blocker of the third generation--also for patients with obstructive lung diseases?]. Z Kardiol. 2001; 90(10):760-5.

136. Sadiq SA, Fielding K, Vernon SA: The effect of timolol drops on respiratory function. Eye (Lond). 1998; 12 ( Pt 3a):386-9.

137. Riley M, Elborn JS, Khan MM, Stanford CF, Nicholls DP: Comparative effects of epanolol and diltiazem on exercise performance and respiratory gas exchange in angina pectoris. Eur Heart J. 1992; 13(8):1116-22.

138. Ryden L: Efficacy of epanolol versus metoprolol in angina pectoris: report from a Swedish multicentre study of exercise tolerance. Journal of internal medicine. 1992; 231(1):7-11.

139. Lofdahl CG, Dahlof C, Westergren G, Olofsson B, Svedmyr N: Controlled-release metoprolol compared with atenolol in asthmatic patients: interaction with terbutaline. Eur J Clin Pharmacol. 1988; 33 Suppl:S25-32.

140. Richards R, Tattersfield AE: Comparison of the airway response to eye drops of timolol and its isomer L-714,465 in asthmatic subjects. Br J Clin Pharmacol. 1987; 24(4):485-91.

141. Lofdahl CG, Svedmyr N: Selectivity of beta-adrenergic stimulating and blocking agents. Eur J Respir Dis Suppl. 1984; 136:101-13.

142. Lofdahl CG, Marlin GE, Svedmyr N: The effects of pafenolol and metoprolol on ventilatory function and haemodynamics during exercise by asthmatic patients. Eur J Clin Pharmacol. 1983; 24(3):289-95.

143. Ruffin RE, Frith PA, Anderton RC, Kumana CR, Newhouse MT, Hargreave FE: Selectivity of beta adrenoreceptor antagonist drugs assessed by histamine bronchial provocation. Clin Pharmacol Ther. 1979; 25(5 Pt 1):536-40.

144. Maconochie JG, Woodings EP, Richards DA: Effects of labetalol and propranolol on histamine-induced bronchoconstriction in normal subjects. Br J Clin Pharmacol. 1977; 4(2):157-62.

145. Steptoe A, Ronaldson A, Kostich K, Lazzarino AI, Urbanova L, Carvalho LA: The effect of beta-adrenergic blockade on inflammatory and cardiovascular responses to acute mental stress. Brain, behavior, and immunity. 2018; 70:369-75.

146. Statsenko ME, Derevianchenko MV, Chernikov MV, Lopushkova Iu E: [Efficacy and safety of bisoprololal in hypertensive patients with cardiovascular disease and chronic obstructive pulmonary disease]. Kardiologiia. 2014; 54(1):48-54.

147. Short PM, Anderson WJ, Williamson PA, Lipworth BJ: Effects of intravenous and oral beta-blockade in persistent asthmatics controlled on inhaled corticosteroids. Heart. 2014; 100(3):219-23.

148. Short PM, Williamson PA, Anderson WJ, Lipworth BJ: Randomized placebo-controlled trial to evaluate chronic dosing effects of propranolol in asthma. Am J Respir Crit Care Med. 2013; 187(12):1308-14.

149. van der Woude HJ, Zaagsma J, Postma DS, Winter TH, van Hulst M, Aalbers R: Detrimental effects of beta-blockers in COPD: a concern for nonselective beta-blockers. Chest. 2005; 127(3):818-24.

150. Schultze-Werninghaus G, Siekmeier R, Laxy T: [Comparison of the characteristics of methacholine and of propranolol in the assessment of aspecific hyperreactivity of the airways]. Pneumologie. 1991; 45(9):729-33.

151. Vilsvik JS, Schaanning J: Effect of atenolol on ventilatory and cardiac function in asthma. British medical journal. 1976; 2(6033):453-5.

152. Skinner C, Gaddie J, Palmer KN: Comparison of effects of metoprolol and propranolol on asthmatic airway obstruction. British medical journal. 1976; 1(6008):504.

153. Skinner C, Palmer KN: Comparison of the effects of acebutolol (Sectral) and practolol (Eraldin) on airways obstruction in asthmatics. Br J Clin Pharmacol. 1975; 2(5):417-22.

154. Skinner C, Gaddie J, Palmer KN: Comparison of intravenous AH 5158 (ibidomide) and propranolol in asthma. British medical journal. 1975; 2(5962):59-61.

155. Bauer K, Kaik G, Kaik B: Osmotic release oral drug delivery system of metoprolol in hypertensive asthmatic patients. Pharmacodynamic effects on beta 2-adrenergic receptors. Hypertension. 1994; 24(3):339-46.

156. Warren JB, Jennings SJ, Clark TJ: Effect of adrenergic and vagal blockade on the normal human airway response to exercise. Clin Sci (Lond). 1984; 66(1):79-85.

157. Leidy NK, Schmier JK, Jones MK, Lloyd J, Rocchiccioli K: Evaluating symptoms in chronic obstructive pulmonary disease: validation of the Breathlessness, Cough and Sputum Scale. Respir Med. 2003; 97 Suppl A:S59-70.

158. Menezes MD, McCarter R, Greene EA, Bauman NM: Status of propranolol for treatment of infantile hemangioma and description of a randomized clinical trial. Ann Otol Rhinol Laryngol. 2011; 120(10):686-95.

159. Lama PJ: Systemic adverse effects of beta-adrenergic blockers: an evidence-based assessment. Am J Ophthalmol. 2002; 134(5):749-60.

**eTable 5:** Characteristics of the included studies

| Study name | Underline disease | Definition of acute asthma attack | Comparison | Numbers | mean age | female (%) | Treatment duration | Baseline asthma | Study design | Country |
| --- | --- | --- | --- | --- | --- | --- | --- | --- | --- | --- |
| Lainscak, M. (2011)[1] | established CHF (LVEF 40%) and COPD | wheezing, dyspnea | bisoprolol mean 6.4mg carvedilol mean 47 mg | 32 31 | 73.0±9.0 | 19.0 | 4-6 weeks maintenance | exclude | randomized open-label | Slovenia |
| Morgan, T.O. (2001)[2] | aged 65 to 86 years with systolic hypertension and exclude asthma history | Wheeze | atenolol (25 to 50 mg) placebo | 58 44 | 77.3 | 24.3 | 2 months | exclude | balanced randomized crossover design | Australia |
| Evrard, P. (2000)[3] | patients refer to coronary artery bypass grafting without cardiac concomitant procedures | Asthma attack | sotalol 80 mg BID control | 103 103 | 61.0±9.0 | 11.0 | 1 day before surgery | exclude | randomized, open label trial | Belgium |
| Bayliff, C.D. (1999)[4] | Patients undergoing major thoracic operation for pneumonectomy, esophagectomy, or lobectomy | marked symptomatic bronchospasm | propranolol 40 mg placebo | 49 50 | 62.4±10.4 | 38.4 | 6 days | exclude | randomized, double-blind | Canada |
| Sung, R.J. (1995)[5] | patients with supraventricular tachycardias or atrial fibrillation/flutter | wheezing or dyspnea | infusion of sotalol placebo | 64 29 | 52.8 | 33.3 | once before test | exclude | randomized, double-blind | USA |
| Pujet, J.C. (1992)[6] | stable asthma with reversible bronchoconstriction | wheezing, dyspnea | celiprolol 400mg bisoprolol 20mg propranolol 40 mg celiprolol 400mg + propranolol 40 mg | 10 10 10 10 | 31.0±7.6 | 60.0 | once before test | yes | randomized, double-blind, cross-over | France |
| Boulet, L.P. (1990)[7] | mild to moderate hypertension | increased wheeziness or dyspnea | dilevalol 200 mg atenolol 50mg | 9 9 | 44.2±10.0 | 50.0 | 14 weeks | Not specific | randomized, double-blind | Canada |
| Chodosh, S. (1988)[8] | patients with asthma | marked symptomatic bronchospasm | dilevalol 400mg metoprolol 200 mg placebo | 16 16 16 | 39.0 | 50.0 | once before test | yes | randomized, double-blind, crossover | USA |
| Duff, G.R. (1987)[9] | patients with glaucoma or ocular hypertension | wheezing or dyspnea | nadolol 20-40mg placebo | 6 4 | na | na | 10 weeks | exclude | randomized, single-blind, crossover | UK |
| McGill, D. (1986)[10] | chronic stable angina | increased wheeziness or dyspnea | propranolol 120 mg placebo | 23 25 | 61.0 | 8.0 | 4 weeks | Not specific | randomized, double-blind, cross-over | Australia |
| Schindl, R. (1986)[11] | patients with asthma and hypertension | wheezing or dyspnea | atenolol 100mg celiprolol 200mg metoprolol 200 mg | 18 18 18 | 60.0 | 22.2 | once before test | yes | randomized, double-blind, crossover | Australia |
| Sheppard, D. (1986)[12] | mild bronchial asthma | marked symptomatic bronchoconstriction | infusion of esmolol infusion of propranolol placebo | 10 6 10 | 30.8±1.32 | 0.0 | gradually titrate infusion dosage before test | yes | randomized, double-blind, cross-over | USA |
| Wilcox, P.G. (1986)[13] | patients with asthma | wheezing or dyspnea | metoprolol 187.5 mg bevantolol 281.25mg | 15 16 | 42.4±15.3 | 18.8 | gradually titrate oral dosage before test | yes | randomized, double-blind, crossover | Canada |
| Clague, H.W. (1984)[14] | asthma (reversible airways obstruction) | wheezing or dyspnea | propranolol 10-40 mg metoprolol 50-100 mg | 8 8 | na | na | gradually titrate oral dosage before test | yes | randomized, single-blind, crossover | Canada |
| Simon, H. (1983)[15] | Patients with angina | wheezing or dyspnea | carteolol pindolol | 20 20 | na | na | 4 weeks | Not specific | randomized, double-blind | Germany |
| Cannon, R.E. (1982)[16] | patients with asthma | marked symptomatic bronchospasm | pindolol 2.5-20mg placebo | 9 6 | na | na | once before test | yes | randomized, double-blind | USA |
| Larsson, K. (1982)[17] | patients with asthma | marked symptomatic bronchoconstriction | infusion of propranolol infusion of propranolol and labetalol infusion of practolol labetalol 400 mg (oral) placebo | 14 14 14 14 28 | na | na | once before test | yes | randomized, placebo-control, crossover | Sweden |
| Lawrence, D.S. (1982) study 2[18] | reversible airways obstruction and co-existent hypertension | Asthma exacerbation | atenolol 100 mg metoprolol 200 mg placebo | 14 13 14 | 55.7±10.6 | 14.3 | 3 weeks | yes | randomized, single blind crossover study | UK |
| Ellis, M.E. (1981)[19] | asthmatic patients with reversible airways obstruction continuing use disodium cromoglycate or steroids | increased wheeziness or dyspnea | atenolol 50-200mg propranolol 40 mg placebo | 10 10 10 | 43.0 | 30.0 | once 1.5-2 hour before test | yes | randomized, double-blind, cross-over | UK |
| Benson, M.K. (1978)[20] | patients (age 17-67) history of episodic dyspnoea and wheeze with spontaneous variability of airways obstruction of at least 15%, but at the time of the study were in clinical remission | increased wheeziness or shortness of breath (FEV1 decreased 20-70%) | propranolol 100 mg pindolol 5 mg atenolol 100 mg acebutolol 300 mg | 13 13 12 13 | 32.2±16.8 | 7.1 | once 1-2 hour before test | yes | single-blind, randomized, crossover | UK |
| Decalmer, P.B. (1978)[21] | asthmatic patients with reversible airways obstruction continuing use disodium cromoglycate or steroids | Wheeze | atenolol 100mg metoprolol 100 mg acebutoiol 300 mg propranolol 100mg oxprenolol 100 mg timolol 10mg pindolol 5 mg placebo | 10 9 5 10 8 4 5 10 | 41.3±19.8 | 10.0 | once 1 hour before test | yes | single-blind randomized, crossover | UK |
| Amsterdam, E.A. (1976)[22] | patients with supraventricular tachyarrhythmias or ventricular arrhythmias | wheezing or dyspnea | infusion of tolamolol placebo | 27 27 | 64.0 | 35.7 | once before test | Not specific | randomized, double-blind, cross-over | USA |
| Leary, W.P. (1973)[23] | patients with asthma | marked symptomatic bronchospasm | acebutolol 100-200mg practolol 100-200mg placebo | 15 15 15 | 22.0±4.0 | 0.0 | once before test | yes | randomized, single-blind, crossover | South Africa |
| Bonn, J.A. (1972)[24] | patients with most prominent symptoms attributable to anxiety with/without asthma history | Asthma exacerbation | practolol 200 mg BID placebo | 13 13 | na | na | 2 weeks | Not specific | randomized, double-blind, cross-over | UK |

Abbreviation: na: not available; UK: United Kingdom; USA: United States of America

**References:**

1. Lainscak M, Podbregar M, Kovacic D, Rozman J, von Haehling S: Differences between bisoprolol and carvedilol in patients with chronic heart failure and chronic obstructive pulmonary disease: a randomized trial. Respir Med. 2011; 105 Suppl 1:S44-9.

2. Morgan TO, Anderson AI, MacInnis RJ: ACE inhibitors, beta-blockers, calcium blockers, and diuretics for the control of systolic hypertension. Am J Hypertens. 2001; 14(3):241-7.

3. Evrard P, Gonzalez M, Jamart J, Malhomme B, Blommaert D, Eucher P, Installe E: Prophylaxis of supraventricular and ventricular arrhythmias after coronary artery bypass grafting with low-dose sotalol. Ann Thorac Surg. 2000; 70(1):151-6.

4. Bayliff CD, Massel DR, Inculet RI, Malthaner RA, Quinton SD, Powell FS, Kennedy RS: Propranolol for the prevention of postoperative arrhythmias in general thoracic surgery. Ann Thorac Surg. 1999; 67(1):182-6.

5. Sung RJ, Tan HL, Karagounis L, Hanyok JJ, Falk R, Platia E, Das G, Hardy SA: Intravenous sotalol for the termination of supraventricular tachycardia and atrial fibrillation and flutter: a multicenter, randomized, double-blind, placebo-controlled study. Sotalol Multicenter Study Group. Am Heart J. 1995; 129(4):739-48.

6. Pujet JC, Dubreuil C, Fleury B, Provendier O, Abella ML: Effects of celiprolol, a cardioselective beta-blocker, on respiratory function in asthmatic patients. Eur Respir J. 1992; 5(2):196-200.

7. Boulet LP, Lacourciere Y, Milot J, Lampron N: Comparative effects of dilevalol and atenolol on lung function and airway response to methacholine in hypertensive subjects. Br J Clin Pharmacol. 1990; 29(6):725-31.

8. Chodosh S, Tuck J, Blasucci DJ: The effects of dilevalol, metoprolol, and placebo on ventilatory function in asthmatics. J Cardiovasc Pharmacol. 1988; 11 Suppl 2:S18-24.

9. Duff GR: The effect of twice daily nadolol on intraocular pressure. Am J Ophthalmol. 1987; 104(4):343-5.

10. McGill D, McKenzie W, McCredie M: Comparison of nicardipine and propranolol for chronic stable angina pectoris. Am J Cardiol. 1986; 57(1):39-43.

11. Schindl R, Wurtz J, Hoffmann H: The effect of the cardioselective beta blocker celiprolol on pulmonary function in asthmatic patients. J Cardiovasc Pharmacol. 1986; 8 Suppl 4:S99-101.

12. Sheppard D, DiStefano S, Byrd RC, Eschenbacher WL, Bell V, Steck J, Laddu A: Effects of esmolol on airway function in patients with asthma. Journal of clinical pharmacology. 1986; 26(3):169-74.

13. Wilcox PG, Ahmad D, Darke AC, Parsons J, Carruthers SG: Respiratory and cardiac effects of metoprolol and bevantolol in patients with asthma. Clin Pharmacol Ther. 1986; 39(1):29-34.

14. Clague HW, Ahmad D, Carruthers SG: Influence of cardioselectivity and respiratory disease on pulmonary responsiveness to beta-blockade. Eur J Clin Pharmacol. 1984; 27(5):517-23.

15. Simon H, Schuppan U: The treatment of angina pectoris with the new beta-receptor blocker carteolol. Results of a controlled trial in comparison with pindolol. Arzneimittelforschung. 1983; 33(2a):318-21.

16. Cannon RE, Slavin RG, Gonasun LM: The effect on asthma of a new beta blocker, pindolol. Am Heart J. 1982; 104(2 Pt 2):438-42.

17. Larsson K: Influence of labetalol, propranolol and practolol in patients with asthma. Eur J Respir Dis. 1982; 63(3):221-30.

18. Lawrence DS, Sahay JN, Chatterjee SS, Cruickshank JM: Asthma and beta-blockers. Eur J Clin Pharmacol. 1982; 22(6):501-9.

19. Ellis ME, Sahay JN, Chatterjee SS, Cruickshank JM, Ellis SH: Cardioselectivity of atenolol in asthmatic patients. Eur J Clin Pharmacol. 1981; 21(3):173-6.

20. Benson MK, Berrill WT, Cruickshank JM, Sterling GS: A comparison of four beta-adrenoceptor antagonists in patients with asthma. Br J Clin Pharmacol. 1978; 5(5):415-9.

21. Decalmer PB, Chatterjee SS, Cruickshank JM, Benson MK, Sterling GM: Beta-blockers and asthma. Br Heart J. 1978; 40(2):184-9.

22. Amsterdam EA, Lee G, Morrison S, Tonkin MJ, DeMaria AN, Mason DT: Efficacy of cardioselective beta adrenergic blockade with intravenously administered tolamolol in the treatment of cardiac arrhythmias. Am J Cardiol. 1976; 38(2):195-9.

23. Leary WP, Coleman AJ, Asmal AC: Respiratory effects of acebutolol hydrochloride. S Afr Med J. 1973; 47(28):1245-8.

24. Bonn JA, Turner P, Hicks DC: Beta-adrenergic-receptor blockade with practolol in treatment of anxiety. Lancet. 1972; 1(7755):814-5.

**eTable 6A:** SUCRA of the incidence of asthma after beta-blocking agents

| Treatment | SUCRA |
| --- | --- |
| atenolol | 18.3 |
| celiprolol | 19.0 |
| bisoprolol | 25.3 |
| celiprolol + propranolol | 27.9 |
| placebo/control | 34.8 |
| infusion of practolol | 34.8 |
| infusion of sotalol | 37.9 |
| pindolol | 39.2 |
| acebutolol | 41.2 |
| carteolol | 42.2 |
| practolol | 43.2 |
| carvedilol | 47.2 |
| propranolol | 47.2 |
| metoprolol | 48.0 |
| nadolol | 55.8 |
| bevantolol | 61.7 |
| infusion of tolamolol | 62.6 |
| oxprenolol | 65.6 |
| infusion of esmolol | 66.8 |
| labetalol | 71.2 |
| timolol | 71.9 |
| infusion of propranolol and labetalol | 75.3 |
| sotalol | 75.7 |
| infusion of propranolol | 87.0 |

Sorted by efficacy order (the former, the higher incidence of asthma after beta-blocking agents)

Abbreviation: SUCRA: surface under the cumulative ranking curve

**eTable 6B:** SUCRA of the incidence of asthma after beta-blocking agents in patients with baseline asthma diagnosis

| Treatment | SUCRA |
| --- | --- |
| placebo/control | 22.1 |
| celiprolol | 23.8 |
| practolol | 25.9 |
| infusion of practolol | 27.1 |
| atenolol | 27.9 |
| bisoprolol | 33.0 |
| celiprolol + propranolol | 33.6 |
| pindolol | 34.8 |
| acebutolol | 41.7 |
| metoprolol | 56.0 |
| propranolol | 59.2 |
| infusion of esmolol | 60.9 |
| infusion of propranolol and labetalol | 69.2 |
| bevantolol | 69.3 |
| oxprenolol | 74.0 |
| labetalol | 74.4 |
| timolol | 81.4 |
| infusion of propranolol | 85.5 |

Sorted by efficacy order (the former, the higher incidence of asthma after beta-blocking agents)

Abbreviation: SUCRA: surface under the cumulative ranking curve

**eTable 6C:** SUCRA of the incidence of asthma after beta-blocking agents: sensitivity test of removal of zero event

| Treatment | SUCRA |
| --- | --- |
| atenolol | 12.0 |
| acebutolol | 24.7 |
| metoprolol | 43.7 |
| propranolol | 51.6 |
| carteolol | 52.1 |
| infusion of sotalol | 53.2 |
| pindolol | 56.0 |
| placebo/control | 61.0 |
| bevantolol | 66.4 |
| oxprenolol | 79.3 |

Sorted by efficacy order (the former, the higher incidence of asthma after beta-blocking agents)

Abbreviation: SUCRA: surface under the cumulative ranking curve

**eTable 7: Heterogeneity of the primary outcome**

| Test(s) of heterogeneity | | | | | | | | | |
| --- | --- | --- | --- | --- | --- | --- | --- | --- | --- |
| Comparison | | | Heterogeneity statistic | degrees of freedom | p value | I-squared | Tau-squared | Treatments used | |
| C | - | B | 0.02 | 1 | 0.882 | 0.00% | 0 | A: | Pla |
| D | - | B | 2.87 | 2 | 0.238 | 30.30% | 0.9735 | B: | Pro |
| E | - | B | 0.02 | 1 | 0.882 | 0.00% | 0 | C: | Pin |
| D | - | C | 0.09 | 1 | 0.759 | 0.00% | 0 | D: | Ate |
| E | - | C | 0 | 1 | 1.000 | 0.00% | 0 | E: | Ace |
| E | - | D | 0.09 | 1 | 0.759 | 0.00% | 0 | F: | Sot |
| D | - | A | 5.34 | 3 | 0.148 | 43.90% | 0.9472 | G: | Met |
| G | - | A | 1.27 | 2 | 0.530 | 0.00% | 0 | H: | Pra |
| G | - | D | 1.3 | 2 | 0.521 | 0.00% | 0 | I: | Oxp |
| H | - | A | 0.19 | 1 | 0.664 | 0.00% | 0 | J: | Tim |
| B | - | A | 6.41 | 3 | 0.093 | 53.20% | 1.3609 | K: | Nad |
| C | - | A | 0.76 | 1 | 0.382 | 0.00% | 0 | L: | iSot |
| E | - | A | 0.07 | 1 | 0.785 | 0.00% | 0 | M: | Lab |
| I | - | A | 0 | 0 | . | .% | 0 | N: | Bis |
| J | - | A | 0 | 0 | . | .% | 0 | O: | Car |
| G | - | B | 0.11 | 1 | 0.737 | 0.00% | 0 | P: | Cel |
| I | - | B | 0 | 0 | . | .% | 0 | Q: | iEsm |
| J | - | B | 0 | 0 | . | .% | 0 | R: | iPro |
| G | - | C | 0 | 0 | . | .% | 0 | S: | iTol |
| I | - | C | 0 | 0 | . | .% | 0 | T: | Cat |
| J | - | C | 0 | 0 | . | .% | 0 | U: | iPLab |
| I | - | D | 0 | 0 | . | .% | 0 | V: | iPac |
| J | - | D | 0 | 0 | . | .% | 0 | W: | CPro |
| G | - | E | 0 | 0 | . | .% | 0 | X: | Bev |
| I | - | E | 0 | 0 | . | .% | 0 |  |  |
| J | - | E | 0 | 0 | . | .% | 0 |  |  |
| I | - | G | 0 | 0 | . | .% | 0 |  |  |
| J | - | G | 0 | 0 | . | .% | 0 |  |  |
| J | - | I | 0 | 0 | . | .% | 0 |  |  |
| M | - | D | 0 | 0 | . | .% | 0 |  |  |
| O | - | N | 0 | 0 | . | .% | 0 |  |  |
| N | - | B | 0 | 0 | . | .% | 0 |  |  |
| P | - | B | 0 | 0 | . | .% | 0 |  |  |
| W | - | B | 0 | 0 | . | .% | 0 |  |  |
| P | - | N | 0 | 0 | . | .% | 0 |  |  |
| W | - | N | 0 | 0 | . | .% | 0 |  |  |
| W | - | P | 0 | 0 | . | .% | 0 |  |  |
| Q | - | A | 0 | 0 | . | .% | 0 |  |  |
| R | - | A | 0.06 | 1 | 0.806 | 0.00% | 0 |  |  |
| R | - | Q | 0 | 0 | . | .% | 0 |  |  |
| S | - | A | 0 | 0 | . | .% | 0 |  |  |
| U | - | A | 0 | 0 | . | .% | 0 |  |  |
| V | - | A | 0 | 0 | . | .% | 0 |  |  |
| U | - | R | 0 | 0 | . | .% | 0 |  |  |
| V | - | R | 0 | 0 | . | .% | 0 |  |  |
| V | - | U | 0 | 0 | . | .% | 0 |  |  |
| M | - | A | 0.26 | 1 | 0.610 | 0.00% | 0 |  |  |
| L | - | A | 0 | 0 | . | .% | 0 |  |  |
| F | - | A | 0 | 0 | . | .% | 0 |  |  |
| X | - | G | 0 | 0 | . | .% | 0 |  |  |
| H | - | E | 0 | 0 | . | .% | 0 |  |  |
| P | - | D | 0 | 0 | . | .% | 0 |  |  |
| P | - | G | 0 | 0 | . | .% | 0 |  |  |
| K | - | A | 0 | 0 | . | .% | 0 |  |  |
| M | - | G | 0 | 0 | . | .% | 0 |  |  |
| T | - | C | 0 | 0 | . | .% | 0 |  |  |

Abbreviation: Ace: oral acebutolol; Ate: oral atenolol; Bev: oral bevantolol; Bis: oral bisoprolol; Car: oral carvedilol; Cat: oral carteolol; Cel: oral celiprolol; CI: confidence interval; CPro: oral celiprolol and propranolol; ES: effect size; iEsm: infusion of esmolol; iPac: infusion of practolol; iPLab: infusion of propranolol and labetalol; iPro: infusion of propranolol; iSot: infusion of sotalol; iTol: infusion of tolamolol; Lab: oral dilevalol or oral labetalol; Met: oral metoprolol; Nad: oral nadolol; NMA: network meta-analysis; OR: odds ratio; Oxp: oral oxprenolol; Pin: oral pindolol; Pla: Placebo/Control; Pra: oral practolol; Pro: oral propranolol; Sot: oral sotalol; SUCRA: surface under the cumulative ranking curve; Tim: oral timolol

**eTable 8: Inconsistency of different intervention**

**Part 1: design-by-treatment and loop inconsistency model**

| Inconsistency model | chi^2^ | Prob>chi^2^ |
| --- | --- | --- |
| Overall subjects | | |
| design-by-treatment | 4.61 | 0.9997 |
| loop inconsistency | 0.93 | 0.9959 |
| Participants with baseline asthma diagnosis | | |
| design-by-treatment | 6.69 | 0.9656 |
| loop inconsistency | 0.81 | 0.9918 |
| Sensitivity test | | |
| design-by-treatment | 2.54 | 0.7708 |
| loop inconsistency | 2.39 | 0.3030 |

**Part 2: side-splitting inconsistency model:**

Part of overall subjects

| Side | Direct | | Indirect | | Difference | | P>z | tau | Treatments used | |
| --- | --- | --- | --- | --- | --- | --- | --- | --- | --- | --- |
|  | Coef. | Std. Err. | Coef. | Std. Err. | Coef. | Std. Err. |  |  |  |  |
| A B * | 0.312581 | 0.460522 | 0.794478 | 0.729561 | -0.4819 | 0.704978 | 0.494 | 0.395101 | A (reference): | Pla |
| A C * | 0.159715 | 0.516922 | 0.038571 | 1.063813 | 0.121144 | 1.119602 | 0.914 | 0.399508 | B: | Pro |
| A D * | -0.65743 | 0.501597 | -0.661 | 0.998214 | 0.00357 | 1.025436 | 0.997 | 0.387348 | C: | Pin |
| A E * | 0.292161 | 0.802749 | 0.021752 | 1.025307 | 0.270409 | 1.194355 | 0.821 | 0.399621 | D: | Ate |
| A F | . | . | . | . | . | . | . | . | E: | Ace |
| A G * | 0.507567 | 0.578146 | 0.273862 | 0.862016 | 0.233705 | 0.857806 | 0.785 | 0.416544 | F: | Sot |
| A H | . | . | . | . | . | . | . | . | G: | Met |
| A I | . | . | . | . | . | . | . | . | H: | Pra |
| A J | . | . | . | . | . | . | . | . | I: | Oxp |
| A K | . | . | . | . | . | . | . | . | J: | Tim |
| A L | . | . | . | . | . | . | . | . | K: | Nad |
| A M | 1.682669 | 0.988088 | 0.364783 | 1.678697 | 1.317885 | 1.926374 | 0.494 | 0.357133 | L: | iSot |
| A Q | . | . | . | . | . | . | . | . | M: | Lab |
| A R | . | . | . | . | . | . | . | . | N: | Bis |
| A S | . | . | . | . | . | . | . | . | O: | Car |
| A U | . | . | . | . | . | . | . | . | P: | Cel |
| A V | . | . | . | . | . | . | . | . | Q: | iEsm |
| B C * | -0.4188 | 0.639175 | -0.03641 | 0.819384 | -0.38239 | 1.041207 | 0.713 | 0.368733 | R: | iPro |
| B D * | -0.94623 | 0.779898 | -1.09624 | 0.545708 | 0.15001 | 0.883177 | 0.865 | 0.395463 | S: | iTol |
| B E * | -0.42568 | 0.614385 | 1.170978 | 1.494051 | -1.59666 | 1.600173 | 0.318 | 0.29485 | T: | Cat |
| B G * | 0.036895 | 0.488585 | 0.048787 | 0.73082 | -0.01189 | 0.845344 | 0.989 | 0.428182 | U: | iPLab |
| B I | . | . | . | . | . | . | . | . | V: | iPac |
| B J | . | . | . | . | . | . | . | . | W: | CPro |
| B N * | -1.14802 | 1.592226 | -1.07663 | 177.4801 | -0.07139 | 177.4872 | 1 | 0.361541 | X: | Bev |
| B P * | -1.0986 | 1.621027 | -1.49234 | 1.585357 | 0.393739 | 2.267395 | 0.862 | 0.378051 |  |  |
| B W | . | . | . | . | . | . | . | . |  |  |
| C D * | -1.54194 | 1.10459 | -0.64609 | 0.613957 | -0.89585 | 1.122078 | 0.425 | 0.362264 |  |  |
| C E * | -0.22145 | 0.722136 | 1.375211 | 1.492832 | -1.59666 | 1.600173 | 0.318 | 0.29485 |  |  |
| C G * | 0.492157 | 0.719686 | 0.14514 | 0.707992 | 0.347016 | 0.790668 | 0.661 | 0.43883 |  |  |
| C I | . | . | . | . | . | . | . | . |  |  |
| C J | . | . | . | . | . | . | . | . |  |  |
| C T | . | . | . | . | . | . | . | . |  |  |
| D E * | 0.583963 | 0.704564 | 2.180623 | 1.49834 | -1.59666 | 1.600173 | 0.318 | 0.29485 |  |  |
| D G * | 1.180856 | 0.497238 | 0.619903 | 0.895824 | 0.560953 | 0.910848 | 0.538 | 0.422964 |  |  |
| D I | . | . | . | . | . | . | . | . |  |  |
| D J | . | . | . | . | . | . | . | . |  |  |
| D M | 1.098621 | 1.610522 | 2.416506 | 1.056983 | -1.31789 | 1.926374 | 0.494 | 0.357133 |  |  |
| D P | -0.43722 | 1.584889 | -0.04348 | 1.688489 | -0.39374 | 2.267395 | 0.862 | 0.378051 |  |  |
| E G * | 0.398405 | 0.749116 | 0.051389 | 0.788326 | 0.347016 | 0.790668 | 0.661 | 0.43883 |  |  |
| E H | -0.8366 | 1.586724 | 1.052063 | 1.761262 | -1.88867 | 2.324275 | 0.416 | 0.338853 |  |  |
| E I | . | . | . | . | . | . | . | . |  |  |
| E J | . | . | . | . | . | . | . | . |  |  |
| G I | . | . | . | . | . | . | . | . |  |  |
| G J | . | . | . | . | . | . | . | . |  |  |
| G M | 0.888243 | 1.325964 | 0.964853 | 1.184195 | -0.07661 | 1.715095 | 0.964 | 0.379827 |  |  |
| G P | -1.51873 | 1.540028 | -1.12499 | 1.671989 | -0.39374 | 2.267395 | 0.862 | 0.378051 |  |  |
| G X | . | . | . | . | . | . | . | . |  |  |
| I J | . | . | . | . | . | . | . | . |  |  |
| N O | . | . | . | . | . | . | . | . |  |  |
| N P * | -2.24E-07 | 1.990221 | -0.39374 | 2.267348 | 0.393739 | 2.267395 | 0.862 | 0.378051 |  |  |
| N W | . | . | . | . | . | . | . | . |  |  |
| P W | . | . | . | . | . | . | . | . |  |  |
| Q R * | 0.962811 | 0.988153 | 1.466337 | 2.189167 | -0.50353 | 2.115145 | 0.812 | 0.378875 |  |  |
| R U | . | . | . | . | . | . | . | . |  |  |
| R V | . | . | . | . | . | . | . | . |  |  |
| U V | . | . | . | . | . | . | . | . |  |  |

Part of participants with baseline asthma diagnosis

| Side | Direct | | Indirect | | Difference | | P>z | tau | Treatments used | |
| --- | --- | --- | --- | --- | --- | --- | --- | --- | --- | --- |
|  | Coef. | Std. Err. | Coef. | Std. Err. | Coef. | Std. Err. |  |  |  |  |
| A B * | 1.162034 | 0.57187 | 1.298397 | 0.680064 | -0.13636 | 0.657467 | 0.836 | 4.99E-10 | A (reference): | Pla |
| A C * | 0.395981 | 0.423109 | 0.820603 | 1.034551 | -0.42462 | 1.04937 | 0.686 | 2.72E-09 | B: | Pro |
| A D * | 0.315501 | 0.611572 | -0.44344 | 1.132668 | 0.758937 | 1.143966 | 0.507 | 2.23E-10 | C: | Pin |
| A E * | 0.663852 | 0.766694 | 0.666427 | 0.980897 | -0.00258 | 1.118758 | 0.998 | 2.51E-10 | D: | Ate |
| A G * | 1.117052 | 0.532486 | 1.046586 | 0.830461 | 0.070467 | 0.753798 | 0.926 | 4.35E-10 | E: | Ace |
| A H | . | . | . | . | . | . | . | . | F: | CPro |
| A I | . | . | . | . | . | . | . | . | G: | Met |
| A J | . | . | . | . | . | . | . | . | H: | Pra |
| A L | . | . | . | . | . | . | . | . | I: | Oxp |
| A M | . | . | . | . | . | . | . | . | J: | Tim |
| A O | . | . | . | . | . | . | . | . | K: | Bev |
| A Q | . | . | . | . | . | . | . | . | L: | iPac |
| A R | . | . | . | . | . | . | . | . | M: | Lab |
| B C * | -0.57117 | 0.590207 | -1.19255 | 0.821796 | 0.621385 | 0.963426 | 0.519 | 5.92E-09 | N: | Bis |
| B D * | -1.35776 | 0.760473 | -0.78123 | 0.628157 | -0.57653 | 0.93772 | 0.539 | 4.24E-10 | O: | iPLab |
| B E * | -0.59334 | 0.589524 | -0.07012 | 1.683976 | -0.52322 | 1.750763 | 0.765 | 2.61E-10 | P: | Cel |
| B F | . | . | . | . | . | . | . | . | Q: | iEsm |
| B G * | -0.1061 | 0.387153 | -0.08215 | 0.756166 | -0.02394 | 0.808608 | 0.976 | 4.03E-08 | R: | iPro |
| B I | . | . | . | . | . | . | . | . |  |  |
| B J | . | . | . | . | . | . | . | . |  |  |
| B N | . | . | . | . | . | . | . | . |  |  |
| B P * | -1.09863 | 1.576333 | -1.61871 | 1.539445 | 0.520076 | 2.20334 | 0.813 | 3.76E-10 |  |  |
| C D * | -1.22679 | 1.074192 | 0.022622 | 0.641988 | -1.24941 | 1.126382 | 0.267 | 4.56E-08 |  |  |
| C E * | 0.158276 | 0.685698 | 0.681495 | 1.647687 | -0.52322 | 1.750763 | 0.765 | 2.61E-10 |  |  |
| C G * | 0.765235 | 0.593667 | 0.550626 | 0.636069 | 0.214609 | 0.665853 | 0.747 | 2.58E-09 |  |  |
| C I | . | . | . | . | . | . | . | . |  |  |
| C J | . | . | . | . | . | . | . | . |  |  |
| D E * | 0.407861 | 0.709168 | 0.93108 | 1.701959 | -0.52322 | 1.750763 | 0.765 | 2.61E-10 |  |  |
| D G * | 0.940953 | 0.438372 | 0.472348 | 0.860795 | 0.468605 | 0.800301 | 0.558 | 2.82E-10 |  |  |
| D I | . | . | . | . | . | . | . | . |  |  |
| D J | . | . | . | . | . | . | . | . |  |  |
| D P | -0.59595 | 1.542134 | -0.07587 | 1.656837 | -0.52008 | 2.20334 | 0.813 | 1.86E-10 |  |  |
| E G * | 0.520054 | 0.642813 | 0.305445 | 0.731497 | 0.214609 | 0.665853 | 0.747 | 2.58E-09 |  |  |
| E H | . | . | . | . | . | . | . | . |  |  |
| E I | . | . | . | . | . | . | . | . |  |  |
| E J | . | . | . | . | . | . | . | . |  |  |
| F N | . | . | . | . | . | . | . | . |  |  |
| F P * | -2.33E-12 | 1.954017 | -0.52008 | 2.20334 | 0.520076 | 2.20334 | 0.813 | 3.76E-10 |  |  |
| G I | . | . | . | . | . | . | . | . |  |  |
| G J | . | . | . | . | . | . | . | . |  |  |
| G K | . | . | . | . | . | . | . | . |  |  |
| G M | 0.555409 | 1.283632 | 1.110819 | 1.538184 | -0.55541 | 1.933029 | 0.774 | 4.93E-09 |  |  |
| G P | -1.50314 | 1.498825 | -0.98307 | 1.617873 | -0.52008 | 2.20334 | 0.813 | 1.86E-10 |  |  |
| I J | . | . | . | . | . | . | . | . |  |  |
| L O | . | . | . | . | . | . | . | . |  |  |
| L R * | 2.564949 | 1.421447 | 2.061423 | 2.456196 | 0.503526 | 2.046154 | 0.806 | 1.21E-08 |  |  |
| N P * | 2.74E-12 | 1.954017 | -0.52008 | 2.20334 | 0.520076 | 2.20334 | 0.813 | 3.76E-10 |  |  |
| O R * | 0.619039 | 0.553378 | 0.115513 | 2.078128 | 0.503526 | 2.046154 | 0.806 | 1.21E-08 |  |  |
| Q R * | 0.962811 | 0.912634 | 1.466337 | 2.122584 | -0.50353 | 2.046154 | 0.806 | 9.17E-11 |  |  |

Part of sensitivity test

| Side | Direct | | Indirect | | Difference | | P>z | tau | Treatments used | |
| --- | --- | --- | --- | --- | --- | --- | --- | --- | --- | --- |
|  | Coef. | Std. Err. | Coef. | Std. Err. | Coef. | Std. Err. |  |  |  |  |
| A B | -0.26748 | 0.324692 | 0.469024 | 0.634629 | -0.7365 | 0.712867 | 0.302 | 2.53E-08 | A (reference): | Pla |
| A C | 0.287682 | 0.471405 | -1.11985 | 0.779737 | 1.407534 | 0.91116 | 0.122 | 4.58E-10 | B: | Pro |
| A D | -1.18219 | 0.502248 | -0.97295 | 0.772088 | -0.20925 | 0.921071 | 0.82 | 7.55E-05 | C: | Pin |
| A J | . | . | . | . | . | . | . | . | D: | Ate |
| B C * | -0.85737 | 0.725484 | 0.550199 | 0.561188 | -1.40757 | 0.911169 | 0.122 | 5.47E-10 | E: | Ace |
| B E | . | . | . | . | . | . | . | . | F: | Bev |
| B G * | -0.11247 | 0.404189 | -0.32144 | 0.834368 | 0.208966 | 0.921013 | 0.821 | 0.000364 | G: | Met |
| B I | . | . | . | . | . | . | . | . | H: | Cat |
| C E | . | . | . | . | . | . | . | . | I: | Oxp |
| C G * | -0.02065 | 0.615423 | -0.38696 | 0.676403 | 0.366317 | 0.732899 | 0.617 | 1.27E-08 | J: | iSot |
| C H | . | . | . | . | . | . | . | . |  |  |
| C I | . | . | . | . | . | . | . | . |  |  |
| D G | 0.767352 | 0.592971 | 0.976316 | 0.704806 | -0.20896 | 0.921032 | 0.821 | 0.000416 |  |  |
| E G * | 0.667751 | 0.797257 | 0.301434 | 0.909077 | 0.366317 | 0.732899 | 0.617 | 1.27E-08 |  |  |
| E I | . | . | . | . | . | . | . | . |  |  |
| F G * | -0.62861 | 1.170826 | -0.52195 | 96.61159 | -0.10666 | 96.61416 | 0.999 | 9.71E-10 |  |  |
| G I | . | . | . | . | . | . | . | . |  |  |

Abbreviation: Ace: oral acebutolol; Ate: oral atenolol; Bev: oral bevantolol; Bis: oral bisoprolol; Car: oral carvedilol; Cat: oral carteolol; Cel: oral celiprolol; CI: confidence interval; CPro: oral celiprolol and propranolol; ES: effect size; iEsm: infusion of esmolol; iPac: infusion of practolol; iPLab: infusion of propranolol and labetalol; iPro: infusion of propranolol; iSot: infusion of sotalol; iTol: infusion of tolamolol; Lab: oral dilevalol or oral labetalol; Met: oral metoprolol; Nad: oral nadolol; NMA: network meta-analysis; OR: odds ratio; Oxp: oral oxprenolol; Pin: oral pindolol; Pla: Placebo/Control; Pra: oral practolol; Pro: oral propranolol; Sot: oral sotalol; SUCRA: surface under the cumulative ranking curve; Tim: oral timolol

**eTable 9: Estimated between-studies standard deviation of different outcome**

| Outcome | Estimated between-studies standard deviation |
| --- | --- |
| Overall subjects | 0.36153861 |
| Participants with baseline asthma diagnosis | 3.179e-08 |
| Sensitivity test | 5.238e-09 |

**eTable 10: Quality of evidence for primary outcome: changes of severity of acrophobia**

| Comparisons | GRADE | | |
| --- | --- | --- | --- |
|  | Direct | Indirect | Network meta-analysis |
|  | Risk ratio (95% CI) and the final rating of direct evidence | Co-efficiency (Standard error) and the final rating of indirect evidence | Risk ratio (95% CI) and overall quality of evidence |
| Ate vs Cel | 1.00 (0.02,47.85) ⨁◯◯◯ very low | -0.04 (1.69) ⨁⨁◯◯ low | 1.29 (0.13,12.95) ⨁⨁⨁◯ medium |
| Ate vs Bis |  |  | 1.11 (0.04,28.19) ⨁◯◯◯ very low |
| Ate vs CPro |  |  | 1.11 (0.04,28.20) ⨁◯◯◯ very low |
| Ate vs Pla | 0.77 (0.18,3.29) ⨁◯◯◯ very low | -0.66 (1.00) ⨁⨁◯◯ low | 0.51 (0.20,1.28) ⨁⨁⨁◯ medium |
| Ate vs iPac |  |  | 0.64 (0.02,22.67) ⨁◯◯◯ very low |
| Ate vs iSot |  |  | 0.56 (0.04,7.81) ⨁◯◯◯ very low |
| Ate vs Pin | 0.25 (0.03,2.23) ⨁◯◯◯ very low | -0.65 (0.61) ⨁⨁◯◯ low | 0.45 (0.15,1.38) ⨁⨁⨁◯ medium |
| Ate vs Ace | 0.25 (0.03,2.23) ⨁◯◯◯ very low | 2.18 (1.50) ⨁⨁◯◯ low | 0.43 (0.11,1.62) ⨁⨁⨁◯ medium |
| Ate vs Cat |  |  | 0.45 (0.02,9.14) ⨁◯◯◯ very low |
| Ate vs Pra |  |  | 0.43 (0.04,4.69) ⨁◯◯◯ very low |
| Ate vs Car |  |  | 0.36 (0.00,34.97) ⨁◯◯◯ very low |
| Ate vs Pro | 0.36 (0.05,2.71) ⨁◯◯◯ very low | -1.10 (0.55) ⨁⨁◯◯ low | ***0.35 (0.14,0.88)** ⨁⨁⨁⨁ high |
| Ate vs Met | ***0.38 (0.15,0.98)** ⨁⨁⨁◯ medium | 0.62 (0.90) ⨁⨁◯◯ low | ***0.34 (0.14,0.82)** ⨁⨁⨁⨁ high |
| Ate vs Nad |  |  | 0.24 (0.01,5.87) ⨁◯◯◯ very low |
| Ate vs Bev |  |  | 0.18 (0.01,2.34) ⨁◯◯◯ very low |
| Ate vs iTol |  |  | 0.17 (0.01,4.91) ⨁◯◯◯ very low |
| Ate vs Oxp | ***0.06 (0.00,0.97)** ⨁⨁⨁◯ medium |  | ***0.19 (0.06,0.59)** ⨁⨁⨁◯ medium |
| Ate vs iEsm |  |  | 0.14 (0.01,2.12) ⨁◯◯◯ very low |
| Ate vs Lab | 0.33 (0.02,7.25) ⨁◯◯◯ very low | 2.42 (1.06) ⨁⨁◯◯ low | ***0.13 (0.02,0.75)** ⨁⨁⨁⨁ high |
| Ate vs Tim | ***0.05 (0.00,0.77)** ⨁⨁⨁◯ medium |  | ***0.15 (0.05,0.46)** ⨁⨁⨁◯ medium |
| Ate vs iPLab |  |  | 0.09 (0.01,1.09) ⨁⨁◯◯ low |
| Ate vs Sot |  |  | 0.07 (0.00,1.74) ⨁⨁◯◯ low |
| Ate vs iPro |  |  | ***0.05 (0.01,0.48)** ⨁⨁◯◯ low |
| Cel vs Bis | 1.00 (0.02,46.05) ⨁◯◯◯ very low | -0.39 (2.27) ⨁◯◯◯ very low | 0.86 (0.03,28.40) ⨁⨁⨁◯ medium |
| Cel vs CPro | 1.00 (0.02,46.05) ⨁◯◯◯ very low |  | 0.86 (0.03,28.41) ⨁⨁◯◯ low |
| Cel vs Pla |  |  | 0.39 (0.04,4.11) ⨁◯◯◯ very low |
| Cel vs iPac |  |  | 0.49 (0.01,31.93) ⨁◯◯◯ very low |
| Cel vs iSot |  |  | 0.43 (0.01,13.05) ⨁◯◯◯ very low |
| Cel vs Pin |  |  | 0.35 (0.03,3.80) ⨁◯◯◯ very low |
| Cel vs Ace |  |  | 0.33 (0.03,3.88) ⨁◯◯◯ very low |
| Cel vs Cat |  |  | 0.35 (0.01,13.74) ⨁◯◯◯ very low |
| Cel vs Pra |  |  | 0.33 (0.01,8.12) ⨁◯◯◯ very low |
| Cel vs Car |  |  | 0.28 (0.00,32.72) ⨁◯◯◯ very low |
| Cel vs Pro | 0.33 (0.02,7.32) ⨁◯◯◯ very low | -1.49 (1.59) ⨁⨁◯◯ low | 0.27 (0.03,2.50) ⨁⨁⨁◯ medium |
| Cel vs Met | 0.20 (0.01,3.90) ⨁◯◯◯ very low | -1.13 (1.67) ⨁⨁◯◯ low | 0.26 (0.03,2.42) ⨁⨁⨁◯ medium |
| Cel vs Nad |  |  | 0.18 (0.00,8.76) ⨁◯◯◯ very low |
| Cel vs Bev |  |  | 0.14 (0.01,3.68) ⨁◯◯◯ very low |
| Cel vs iTol |  |  | 0.13 (0.00,7.14) ⨁◯◯◯ very low |
| Cel vs Oxp |  |  | 0.15 (0.01,1.51) ⨁⨁◯◯ low |
| Cel vs iEsm |  |  | 0.10 (0.00,3.45) ⨁◯◯◯ very low |
| Cel vs Lab |  |  | 0.10 (0.01,1.65) ⨁⨁◯◯ low |
| Cel vs Tim |  |  | 0.12 (0.01,1.19) ⨁⨁◯◯ low |
| Cel vs iPLab |  |  | 0.07 (0.00,1.89) ⨁⨁◯◯ low |
| Cel vs Sot |  |  | 0.06 (0.00,2.61) ⨁◯◯◯ very low |
| Cel vs iPro |  |  | ***0.04 (0.00,0.88)** ⨁⨁◯◯ low |
| Bis vs CPro | 1.00 (0.02,46.05) ⨁◯◯◯ very low |  | 1.00 (0.02,49.14) ⨁⨁◯◯ low |
| Bis vs Pla |  |  | 0.46 (0.02,11.65) ⨁◯◯◯ very low |
| Bis vs iPac |  |  | 0.57 (0.01,64.96) ⨁◯◯◯ very low |
| Bis vs iSot |  |  | 0.51 (0.01,29.52) ⨁◯◯◯ very low |
| Bis vs Pin |  |  | 0.41 (0.02,10.65) ⨁◯◯◯ very low |
| Bis vs Ace |  |  | 0.39 (0.01,10.72) ⨁◯◯◯ very low |
| Bis vs Cat |  |  | 0.41 (0.01,29.85) ⨁◯◯◯ very low |
| Bis vs Pra |  |  | 0.39 (0.01,19.03) ⨁◯◯◯ very low |
| Bis vs Car | 0.32 (0.01,7.63) ⨁◯◯◯ very low |  | 0.32 (0.01,8.27) ⨁⨁◯◯ low |
| Bis vs Pro | 0.33 (0.02,7.32) ⨁◯◯◯ very low | -1.08 (1760.01) ⨁◯◯◯ very low | 0.32 (0.01,7.19) ⨁⨁⨁◯ medium |
| Bis vs Met |  |  | 0.31 (0.01,7.45) ⨁◯◯◯ very low |
| Bis vs Nad |  |  | 0.21 (0.00,18.51) ⨁◯◯◯ very low |
| Bis vs Bev |  |  | 0.16 (0.00,8.87) ⨁◯◯◯ very low |
| Bis vs iTol |  |  | 0.15 (0.00,14.84) ⨁◯◯◯ very low |
| Bis vs Oxp |  |  | 0.17 (0.01,4.34) ⨁◯◯◯ very low |
| Bis vs iEsm |  |  | 0.12 (0.00,7.70) ⨁◯◯◯ very low |
| Bis vs Lab |  |  | 0.12 (0.00,4.27) ⨁◯◯◯ very low |
| Bis vs Tim |  |  | 0.14 (0.01,3.44) ⨁◯◯◯ very low |
| Bis vs iPLab |  |  | 0.08 (0.00,4.35) ⨁◯◯◯ very low |
| Bis vs Sot |  |  | 0.07 (0.00,5.53) ⨁◯◯◯ very low |
| Bis vs iPro |  |  | 0.04 (0.00,2.09) ⨁◯◯◯ very low |
| CPro vs Pla |  |  | 0.46 (0.02,11.65) ⨁◯◯◯ very low |
| CPro vs iPac |  |  | 0.57 (0.01,64.96) ⨁◯◯◯ very low |
| CPro vs iSot |  |  | 0.51 (0.01,29.52) ⨁◯◯◯ very low |
| CPro vs Pin |  |  | 0.41 (0.02,10.65) ⨁◯◯◯ very low |
| CPro vs Ace |  |  | 0.39 (0.01,10.72) ⨁◯◯◯ very low |
| CPro vs Cat |  |  | 0.41 (0.01,29.85) ⨁◯◯◯ very low |
| CPro vs Pra |  |  | 0.39 (0.01,19.03) ⨁◯◯◯ very low |
| CPro vs Car |  |  | 0.32 (0.00,51.31) ⨁◯◯◯ very low |
| CPro vs Pro | 0.33 (0.02,7.32) ⨁◯◯◯ very low |  | 0.32 (0.01,7.19) ⨁⨁◯◯ low |
| CPro vs Met |  |  | 0.31 (0.01,7.45) ⨁◯◯◯ very low |
| CPro vs Nad |  |  | 0.21 (0.00,18.51) ⨁◯◯◯ very low |
| CPro vs Bev |  |  | 0.16 (0.00,8.87) ⨁◯◯◯ very low |
| CPro vs iTol |  |  | 0.15 (0.00,14.84) ⨁◯◯◯ very low |
| CPro vs Oxp |  |  | 0.17 (0.01,4.34) ⨁◯◯◯ very low |
| CPro vs iEsm |  |  | 0.12 (0.00,7.70) ⨁◯◯◯ very low |
| CPro vs Lab |  |  | 0.12 (0.00,4.28) ⨁◯◯◯ very low |
| CPro vs Tim |  |  | 0.14 (0.01,3.44) ⨁◯◯◯ very low |
| CPro vs iPLab |  |  | 0.08 (0.00,4.35) ⨁◯◯◯ very low |
| CPro vs Sot |  |  | 0.07 (0.00,5.53) ⨁◯◯◯ very low |
| CPro vs iPro |  |  | 0.04 (0.00,2.09) ⨁◯◯◯ very low |
| Pla vs iPac | 1.00 (0.02,47.18) ⨁◯◯◯ very low |  | 1.25 (0.04,39.52) ⨁⨁◯◯ low |
| Pla vs iSot | 0.91 (0.09,9.60) ⨁◯◯◯ very low |  | 1.10 (0.09,12.97) ⨁⨁◯◯ low |
| Pla vs Pin | 0.67 (0.27,1.61) ⨁⨁◯◯ low | 0.04 (1.06) ⨁⨁◯◯ low | 0.88 (0.35,2.22) ⨁⨁⨁◯ medium |
| Pla vs Ace | 0.24 (0.03,2.16) ⨁◯◯◯ very low | 0.02 (1.03) ⨁⨁◯◯ low | 0.85 (0.23,3.16) ⨁⨁⨁◯ medium |
| Pla vs Cat |  |  | 0.88 (0.05,16.73) ⨁◯◯◯ very low |
| Pla vs Pra | 0.51 (0.05,5.81) ⨁◯◯◯ very low |  | 0.84 (0.09,8.24) ⨁⨁◯◯ low |
| Pla vs Car |  |  | 0.70 (0.01,68.71) ⨁◯◯◯ very low |
| Pla vs Pro | 0.56 (0.11,2.72) ⨁◯◯◯ very low | 0.79 (0.73) ⨁⨁◯◯ low | 0.69 (0.29,1.68) ⨁⨁⨁⨁ high |
| Pla vs Met | ***0.14 (0.02,0.82)** ⨁⨁⨁◯ medium | 0.27 (0.86) ⨁⨁◯◯ low | 0.67 (0.24,1.87) ⨁⨁⨁⨁ high |
| Pla vs Nad | 0.47 (0.02,9.26) ⨁◯◯◯ very low |  | 0.47 (0.02,10.06) ⨁⨁◯◯ low |
| Pla vs Bev |  |  | 0.36 (0.03,4.86) ⨁◯◯◯ very low |
| Pla vs iTol | 0.33 (0.01,7.81) ⨁◯◯◯ very low |  | 0.33 (0.01,8.48) ⨁⨁◯◯ low |
| Pla vs Oxp | ***0.06 (0.00,0.97)** ⨁⨁⨁◯ medium |  | 0.37 (0.11,1.24) ⨁⨁⨁◯ medium |
| Pla vs iEsm | 0.33 (0.02,7.30) ⨁◯◯◯ very low |  | 0.27 (0.02,3.53) ⨁⨁◯◯ low |
| Pla vs Lab | 0.18 (0.02,1.49) ⨁⨁◯◯ low | 0.36 (1.68) ⨁⨁◯◯ low | 0.26 (0.05,1.41) ⨁⨁⨁⨁ high |
| Pla vs Tim | ***0.05 (0.00,0.77)** ⨁⨁⨁◯ medium |  | ***0.30 (0.09,0.96)** ⨁⨁⨁⨁ high |
| Pla vs iPLab | 0.14 (0.01,2.53) ⨁◯◯◯ very low |  | 0.18 (0.02,1.80) ⨁⨁⨁◯ medium |
| Pla vs Sot | 0.14 (0.01,2.73) ⨁◯◯◯ very low |  | 0.14 (0.01,2.97) ⨁⨁◯◯ low |
| Pla vs iPro | ***0.10 (0.01,0.73)** ⨁⨁⨁◯ medium |  | ***0.10 (0.01,0.77)** ⨁⨁⨁⨁ high |
| iPac vs iSot |  |  | 0.88 (0.01,61.08) ⨁◯◯◯ very low |
| iPac vs Pin |  |  | 0.71 (0.02,25.03) ⨁◯◯◯ very low |
| iPac vs Ace |  |  | 0.67 (0.02,27.03) ⨁◯◯◯ very low |
| iPac vs Cat |  |  | 0.71 (0.01,65.56) ⨁◯◯◯ very low |
| iPac vs Pra |  |  | 0.67 (0.01,41.93) ⨁◯◯◯ very low |
| iPac vs Car |  |  | 0.56 (0.00,173.48) ⨁◯◯◯ very low |
| iPac vs Pro |  |  | 0.55 (0.02,19.38) ⨁◯◯◯ very low |
| iPac vs Met |  |  | 0.53 (0.01,19.42) ⨁◯◯◯ very low |
| iPac vs Nad |  |  | 0.37 (0.00,37.74) ⨁◯◯◯ very low |
| iPac vs Bev |  |  | 0.28 (0.00,21.45) ⨁◯◯◯ very low |
| iPac vs iTol |  |  | 0.27 (0.00,30.13) ⨁◯◯◯ very low |
| iPac vs Oxp |  |  | 0.30 (0.01,11.42) ⨁◯◯◯ very low |
| iPac vs iEsm |  |  | 0.21 (0.01,6.57) ⨁◯◯◯ very low |
| iPac vs Lab |  |  | 0.21 (0.00,9.65) ⨁◯◯◯ very low |
| iPac vs Tim |  |  | 0.24 (0.01,9.06) ⨁◯◯◯ very low |
| iPac vs iPLab | 0.14 (0.01,2.53) ⨁◯◯◯ very low |  | 0.14 (0.01,2.76) ⨁⨁◯◯ low |
| iPac vs Sot |  |  | 0.11 (0.00,11.28) ⨁◯◯◯ very low |
| iPac vs iPro | 0.08 (0.01,1.25) ⨁⨁◯◯ low |  | 0.08 (0.00,1.38) ⨁⨁◯◯ low |
| iSot vs Pin |  |  | 0.80 (0.06,11.12) ⨁◯◯◯ very low |
| iSot vs Ace |  |  | 0.77 (0.05,12.53) ⨁◯◯◯ very low |
| iSot vs Cat |  |  | 0.80 (0.02,37.14) ⨁◯◯◯ very low |
| iSot vs Pra |  |  | 0.76 (0.03,21.91) ⨁◯◯◯ very low |
| iSot vs Car |  |  | 0.64 (0.00,115.86) ⨁◯◯◯ very low |
| iSot vs Pro |  |  | 0.63 (0.05,8.60) ⨁◯◯◯ very low |
| iSot vs Met |  |  | 0.60 (0.04,8.74) ⨁◯◯◯ very low |
| iSot vs Nad |  |  | 0.42 (0.01,21.68) ⨁◯◯◯ very low |
| iSot vs Bev |  |  | 0.32 (0.01,11.70) ⨁◯◯◯ very low |
| iSot vs iTol |  |  | 0.30 (0.01,17.64) ⨁◯◯◯ very low |
| iSot vs Oxp |  |  | 0.34 (0.02,5.23) ⨁◯◯◯ very low |
| iSot vs iEsm |  |  | 0.24 (0.01,8.59) ⨁◯◯◯ very low |
| iSot vs Lab |  |  | 0.24 (0.01,4.67) ⨁◯◯◯ very low |
| iSot vs Tim |  |  | 0.27 (0.02,4.14) ⨁◯◯◯ very low |
| iSot vs iPLab |  |  | 0.16 (0.01,4.75) ⨁◯◯◯ very low |
| iSot vs Sot |  |  | 0.13 (0.00,6.45) ⨁◯◯◯ very low |
| iSot vs iPro |  |  | 0.09 (0.00,2.22) ⨁◯◯◯ very low |
| Pin vs Ace | 1.00 (0.24,4.24) ⨁◯◯◯ very low | 1.38 (1.49) ⨁⨁◯◯ low | 0.96 (0.25,3.66) ⨁⨁⨁◯ medium |
| Pin vs Cat | 1.00 (0.07,14.90) ⨁◯◯◯ very low |  | 1.00 (0.06,16.33) ⨁⨁◯◯ low |
| Pin vs Pra |  |  | 0.95 (0.09,10.46) ⨁◯◯◯ very low |
| Pin vs Car |  |  | 0.80 (0.01,79.57) ⨁◯◯◯ very low |
| Pin vs Pro | 0.54 (0.17,1.74) ⨁⨁◯◯ low | -0.04 (0.82) ⨁⨁◯◯ low | 0.78 (0.29,2.12) ⨁⨁⨁◯ medium |
| Pin vs Met | 0.56 (0.12,2.60) ⨁◯◯◯ very low | 0.15 (0.71) ⨁⨁◯◯ low | 0.75 (0.25,2.31) ⨁⨁⨁◯ medium |
| Pin vs Nad |  |  | 0.53 (0.02,13.02) ⨁◯◯◯ very low |
| Pin vs Bev |  |  | 0.40 (0.03,5.68) ⨁◯◯◯ very low |
| Pin vs iTol |  |  | 0.38 (0.01,10.90) ⨁◯◯◯ very low |
| Pin vs Oxp | 0.35 (0.08,1.47) ⨁⨁◯◯ low |  | 0.42 (0.12,1.41) ⨁⨁◯◯ low |
| Pin vs iEsm |  |  | 0.30 (0.02,4.69) ⨁◯◯◯ very low |
| Pin vs Lab |  |  | 0.29 (0.05,1.88) ⨁⨁◯◯ low |
| Pin vs Tim | 0.28 (0.07,1.15) ⨁⨁◯◯ low |  | 0.34 (0.10,1.10) ⨁⨁◯◯ low |
| Pin vs iPLab |  |  | 0.20 (0.02,2.42) ⨁◯◯◯ very low |
| Pin vs Sot |  |  | 0.16 (0.01,3.85) ⨁◯◯◯ very low |
| Pin vs iPro |  |  | 0.11 (0.01,1.07) ⨁⨁◯◯ low |
| Ace vs Cat |  |  | 1.04 (0.05,23.15) ⨁◯◯◯ very low |
| Ace vs Pra | 0.33 (0.02,7.58) ⨁◯◯◯ very low | 1.05 (1.76) ⨁⨁◯◯ low | 0.99 (0.09,10.58) ⨁⨁⨁◯ medium |
| Ace vs Car |  |  | 0.83 (0.01,86.16) ⨁◯◯◯ very low |
| Ace vs Pro | 0.54 (0.17,1.74) ⨁⨁◯◯ low | 1.17 (1.49) ⨁⨁◯◯ low | 0.82 (0.26,2.58) ⨁⨁⨁◯ medium |
| Ace vs Met | 0.56 (0.12,2.60) ⨁◯◯◯ very low | 0.05 (0.79) ⨁⨁◯◯ low | 0.79 (0.23,2.74) ⨁⨁⨁◯ medium |
| Ace vs Nad |  |  | 0.55 (0.02,15.57) ⨁◯◯◯ very low |
| Ace vs Bev |  |  | 0.42 (0.03,6.28) ⨁◯◯◯ very low |
| Ace vs iTol |  |  | 0.39 (0.01,12.96) ⨁◯◯◯ very low |
| Ace vs Oxp | 0.35 (0.08,1.47) ⨁⨁◯◯ low |  | 0.44 (0.12,1.58) ⨁⨁◯◯ low |
| Ace vs iEsm |  |  | 0.31 (0.02,5.75) ⨁◯◯◯ very low |
| Ace vs Lab |  |  | 0.31 (0.04,2.33) ⨁◯◯◯ very low |
| Ace vs Tim | 0.28 (0.07,1.15) ⨁⨁◯◯ low |  | 0.35 (0.10,1.23) ⨁⨁◯◯ low |
| Ace vs iPLab |  |  | 0.21 (0.01,3.00) ⨁◯◯◯ very low |
| Ace vs Sot |  |  | 0.17 (0.01,4.61) ⨁◯◯◯ very low |
| Ace vs iPro |  |  | 0.12 (0.01,1.34) ⨁⨁◯◯ low |
| Cat vs Pra |  |  | 0.95 (0.02,37.73) ⨁◯◯◯ very low |
| Cat vs Car |  |  | 0.80 (0.00,173.76) ⨁◯◯◯ very low |
| Cat vs Pro |  |  | 0.78 (0.04,15.19) ⨁◯◯◯ very low |
| Cat vs Met |  |  | 0.75 (0.04,15.27) ⨁◯◯◯ very low |
| Cat vs Nad |  |  | 0.53 (0.01,37.06) ⨁◯◯◯ very low |
| Cat vs Bev |  |  | 0.40 (0.01,18.88) ⨁◯◯◯ very low |
| Cat vs iTol |  |  | 0.38 (0.00,29.87) ⨁◯◯◯ very low |
| Cat vs Oxp |  |  | 0.42 (0.02,8.83) ⨁◯◯◯ very low |
| Cat vs iEsm |  |  | 0.30 (0.01,15.14) ⨁◯◯◯ very low |
| Cat vs Lab |  |  | 0.29 (0.01,8.41) ⨁◯◯◯ very low |
| Cat vs Tim |  |  | 0.34 (0.02,6.99) ⨁◯◯◯ very low |
| Cat vs iPLab |  |  | 0.20 (0.00,8.49) ⨁◯◯◯ very low |
| Cat vs Sot |  |  | 0.16 (0.00,11.05) ⨁◯◯◯ very low |
| Cat vs iPro |  |  | 0.11 (0.00,4.04) ⨁◯◯◯ very low |
| Pra vs Car |  |  | 0.84 (0.01,133.70) ⨁◯◯◯ very low |
| Pra vs Pro |  |  | 0.82 (0.08,8.64) ⨁◯◯◯ very low |
| Pra vs Met |  |  | 0.79 (0.07,8.79) ⨁◯◯◯ very low |
| Pra vs Nad |  |  | 0.56 (0.01,25.49) ⨁◯◯◯ very low |
| Pra vs Bev |  |  | 0.42 (0.01,12.67) ⨁◯◯◯ very low |
| Pra vs iTol |  |  | 0.40 (0.01,20.82) ⨁◯◯◯ very low |
| Pra vs Oxp |  |  | 0.44 (0.04,5.18) ⨁◯◯◯ very low |
| Pra vs iEsm |  |  | 0.32 (0.01,9.99) ⨁◯◯◯ very low |
| Pra vs Lab |  |  | 0.31 (0.02,5.12) ⨁◯◯◯ very low |
| Pra vs Tim |  |  | 0.35 (0.03,4.09) ⨁◯◯◯ very low |
| Pra vs iPLab |  |  | 0.21 (0.01,5.47) ⨁◯◯◯ very low |
| Pra vs Sot |  |  | 0.17 (0.00,7.58) ⨁◯◯◯ very low |
| Pra vs iPro |  |  | 0.12 (0.01,2.54) ⨁◯◯◯ very low |
| Car vs Pro |  |  | 0.98 (0.01,88.32) ⨁◯◯◯ very low |
| Car vs Met |  |  | 0.95 (0.01,89.54) ⨁◯◯◯ very low |
| Car vs Nad |  |  | 0.66 (0.00,164.21) ⨁◯◯◯ very low |
| Car vs Bev |  |  | 0.50 (0.00,86.57) ⨁◯◯◯ very low |
| Car vs iTol |  |  | 0.47 (0.00,128.82) ⨁◯◯◯ very low |
| Car vs Oxp |  |  | 0.53 (0.01,51.47) ⨁◯◯◯ very low |
| Car vs iEsm |  |  | 0.38 (0.00,72.84) ⨁◯◯◯ very low |
| Car vs Lab |  |  | 0.37 (0.00,46.14) ⨁◯◯◯ very low |
| Car vs Tim |  |  | 0.42 (0.00,40.95) ⨁◯◯◯ very low |
| Car vs iPLab |  |  | 0.25 (0.00,42.75) ⨁◯◯◯ very low |
| Car vs Sot |  |  | 0.20 (0.00,49.27) ⨁◯◯◯ very low |
| Car vs iPro |  |  | 0.14 (0.00,21.16) ⨁◯◯◯ very low |
| Pro vs Met | 1.20 (0.55,2.61) ⨁◯◯◯ very low | 0.05 (0.73) ⨁⨁◯◯ low | 0.96 (0.44,2.10) ⨁⨁⨁◯ medium |
| Pro vs Nad |  |  | 0.67 (0.03,16.47) ⨁◯◯◯ very low |
| Pro vs Bev |  |  | 0.51 (0.04,6.42) ⨁◯◯◯ very low |
| Pro vs iTol |  |  | 0.48 (0.02,13.80) ⨁◯◯◯ very low |
| Pro vs Oxp | 0.69 (0.34,1.42) ⨁⨁◯◯ low |  | 0.54 (0.22,1.32) ⨁⨁◯◯ low |
| Pro vs iEsm |  |  | 0.39 (0.02,5.94) ⨁◯◯◯ very low |
| Pro vs Lab |  |  | 0.38 (0.06,2.23) ⨁◯◯◯ very low |
| Pro vs Tim | 0.56 (0.29,1.07) ⨁⨁◯◯ low |  | 0.43 (0.18,1.01) ⨁⨁◯◯ low |
| Pro vs iPLab |  |  | 0.26 (0.02,3.05) ⨁◯◯◯ very low |
| Pro vs Sot |  |  | 0.21 (0.01,4.87) ⨁◯◯◯ very low |
| Pro vs iPro |  |  | 0.14 (0.01,1.34) ⨁⨁◯◯ low |
| Met vs Nad |  |  | 0.70 (0.03,17.88) ⨁◯◯◯ very low |
| Met vs Bev | 0.53 (0.05,5.29) ⨁◯◯◯ very low |  | 0.53 (0.05,5.89) ⨁⨁◯◯ low |
| Met vs iTol |  |  | 0.50 (0.02,14.94) ⨁◯◯◯ very low |
| Met vs Oxp | 0.62 (0.28,1.38) ⨁⨁◯◯ low |  | 0.56 (0.22,1.44) ⨁⨁◯◯ low |
| Met vs iEsm |  |  | 0.40 (0.02,6.50) ⨁◯◯◯ very low |
| Met vs Lab | 0.33 (0.01,7.63) ⨁◯◯◯ very low | 0.96 (1.18) ⨁⨁◯◯ low | 0.39 (0.07,2.32) ⨁⨁⨁◯ medium |
| Met vs Tim | 0.50 (0.24,1.05) ⨁⨁◯◯ low |  | 0.45 (0.18,1.11) ⨁⨁◯◯ low |
| Met vs iPLab |  |  | 0.27 (0.02,3.35) ⨁◯◯◯ very low |
| Met vs Sot |  |  | 0.21 (0.01,5.29) ⨁◯◯◯ very low |
| Met vs iPro |  |  | 0.15 (0.01,1.48) ⨁⨁◯◯ low |
| Nad vs Bev |  |  | 0.76 (0.01,42.98) ⨁◯◯◯ very low |
| Nad vs iTol |  |  | 0.71 (0.01,61.84) ⨁◯◯◯ very low |
| Nad vs Oxp |  |  | 0.80 (0.03,21.57) ⨁◯◯◯ very low |
| Nad vs iEsm |  |  | 0.57 (0.01,31.62) ⨁◯◯◯ very low |
| Nad vs Lab |  |  | 0.56 (0.02,18.52) ⨁◯◯◯ very low |
| Nad vs Tim |  |  | 0.64 (0.02,17.10) ⨁◯◯◯ very low |
| Nad vs iPLab |  |  | 0.38 (0.01,17.87) ⨁◯◯◯ very low |
| Nad vs Sot |  |  | 0.31 (0.00,22.95) ⨁◯◯◯ very low |
| Nad vs iPro |  |  | 0.21 (0.01,8.51) ⨁◯◯◯ very low |
| Bev vs iTol |  |  | 0.94 (0.01,60.10) ⨁◯◯◯ very low |
| Bev vs Oxp |  |  | 1.05 (0.08,13.82) ⨁◯◯◯ very low |
| Bev vs iEsm |  |  | 0.75 (0.02,29.73) ⨁◯◯◯ very low |
| Bev vs Lab |  |  | 0.73 (0.04,14.55) ⨁◯◯◯ very low |
| Bev vs Tim |  |  | 0.84 (0.06,10.93) ⨁◯◯◯ very low |
| Bev vs iPLab |  |  | 0.50 (0.02,16.41) ⨁◯◯◯ very low |
| Bev vs Sot |  |  | 0.40 (0.01,22.07) ⨁◯◯◯ very low |
| Bev vs iPro |  |  | 0.28 (0.01,7.73) ⨁◯◯◯ very low |
| iTol vs Oxp |  |  | 1.11 (0.04,35.24) ⨁◯◯◯ very low |
| iTol vs iEsm |  |  | 0.80 (0.01,50.30) ⨁◯◯◯ very low |
| iTol vs Lab |  |  | 0.78 (0.02,30.00) ⨁◯◯◯ very low |
| iTol vs Tim |  |  | 0.89 (0.03,27.96) ⨁◯◯◯ very low |
| iTol vs iPLab |  |  | 0.54 (0.01,28.59) ⨁◯◯◯ very low |
| iTol vs Sot |  |  | 0.43 (0.01,36.20) ⨁◯◯◯ very low |
| iTol vs iPro |  |  | 0.29 (0.01,13.69) ⨁◯◯◯ very low |
| Oxp vs iEsm |  |  | 0.72 (0.04,12.50) ⨁◯◯◯ very low |
| Oxp vs Lab |  |  | 0.70 (0.10,4.79) ⨁◯◯◯ very low |
| Oxp vs Tim | 0.80 (0.49,1.32) ⨁⨁◯◯ low |  | 0.80 (0.34,1.91) ⨁⨁◯◯ low |
| Oxp vs iPLab |  |  | 0.48 (0.04,6.48) ⨁◯◯◯ very low |
| Oxp vs Sot |  |  | 0.38 (0.01,10.07) ⨁◯◯◯ very low |
| Oxp vs iPro |  |  | 0.26 (0.02,2.89) ⨁◯◯◯ very low |
| iEsm vs Lab |  |  | 0.98 (0.04,21.41) ⨁◯◯◯ very low |
| iEsm vs Tim |  |  | 1.12 (0.07,19.21) ⨁◯◯◯ very low |
| iEsm vs iPLab |  |  | 0.67 (0.07,6.55) ⨁◯◯◯ very low |
| iEsm vs Sot |  |  | 0.54 (0.01,28.86) ⨁◯◯◯ very low |
| iEsm vs iPro | 0.38 (0.06,2.28) ⨁◯◯◯ very low | 1.47 (2.19) ⨁◯◯◯ very low | 0.37 (0.06,2.46) ⨁⨁⨁◯ medium |
| Lab vs Tim |  |  | 1.15 (0.17,7.70) ⨁◯◯◯ very low |
| Lab vs iPLab |  |  | 0.69 (0.04,12.00) ⨁◯◯◯ very low |
| Lab vs Sot |  |  | 0.55 (0.02,17.71) ⨁◯◯◯ very low |
| Lab vs iPro |  |  | 0.38 (0.03,5.44) ⨁◯◯◯ very low |
| Tim vs iPLab |  |  | 0.60 (0.05,7.95) ⨁◯◯◯ very low |
| Tim vs Sot |  |  | 0.48 (0.02,12.40) ⨁◯◯◯ very low |
| Tim vs iPro |  |  | 0.33 (0.03,3.54) ⨁◯◯◯ very low |
| iPLab vs Sot |  |  | 0.80 (0.02,36.07) ⨁◯◯◯ very low |
| iPLab vs iPro | 0.54 (0.18,1.59) ⨁⨁◯◯ low |  | 0.55 (0.15,1.99) ⨁⨁◯◯ low |
| Sot vs iPro |  |  | 0.69 (0.02,26.99) ⨁◯◯◯ very low |

We examined the quality assessment according to the article published in BMJ (Puhan et al., 2014) and another article published in Lancet (Cipriani et al., 2018).

**References:**

Cipriani, A., Furukawa, T.A., Salanti, G., Chaimani, A., Atkinson, L.Z., Ogawa, Y., Leucht, S., Ruhe, H.G., Turner, E.H., Higgins, J.P.T., Egger, M., Takeshima, N., Hayasaka, Y., Imai, H., Shinohara, K., Tajika, A., Ioannidis, J.P.A., Geddes, J.R., 2018. Comparative efficacy and acceptability of 21 antidepressant drugs for the acute treatment of adults with major depressive disorder: a systematic review and network meta-analysis. Lancet 391, 1357-1366.

Puhan, M.A., Schunemann, H.J., Murad, M.H., Li, T., Brignardello-Petersen, R., Singh, J.A., Kessels, A.G., Guyatt, G.H., Group, G.W., 2014. A GRADE Working Group approach for rating the quality of treatment effect estimates from network meta-analysis. BMJ 349, g5630.

Abbreviation: Ace: oral acebutolol; Ate: oral atenolol; Bev: oral bevantolol; Bis: oral bisoprolol; Car: oral carvedilol; Cat: oral carteolol; Cel: oral celiprolol; CI: confidence interval; CPro: oral celiprolol and propranolol; ES: effect size; iEsm: infusion of esmolol; iPac: infusion of practolol; iPLab: infusion of propranolol and labetalol; iPro: infusion of propranolol; iSot: infusion of sotalol; iTol: infusion of tolamolol; Lab: oral dilevalol or oral labetalol; Met: oral metoprolol; Nad: oral nadolol; NMA: network meta-analysis; OR: odds ratio; Oxp: oral oxprenolol; Pin: oral pindolol; Pla: Placebo/Control; Pra: oral practolol; Pro: oral propranolol; Sot: oral sotalol; SUCRA: surface under the cumulative ranking curve; Tim: oral timolol.
